# Endoplasmic Reticulum Stress-Related Four-Biomarker Risk Classifier for Survival Evaluation in Esophageal Cancer

**DOI:** 10.1155/2022/5860671

**Published:** 2022-03-18

**Authors:** Xiang Xu, Yeqing Tang, Jiashan Zhu, Jinhua Luo

**Affiliations:** ^1^Department of Thoracic Surgery, The First Affiliated Hospital of Nanjing Medical University, Nanjing, Jiangsu, China; ^2^Department of Thoracic Surgery, Taixing People's Hospital, Taixing, Jiangsu, China

## Abstract

**Purpose:**

Esophageal cancer (EC) is a lethal digestive tumor worldwide with a dismal clinical outcome. Endoplasmic reticulum (ER) stress poses essential implications for a variety of tumor malignant behaviors. Here, we set up an ER stress-based risk classifier for assessing patient outcome and exploiting robust targets for medical decision-making of EC cases.

**Methods:**

340 EC cases with transcriptome and survival data from two independent public datasets (TCGA and GEO) were recruited for this project. Cox regression analyses were employed to create a risk classifier based on ER stress-related genes (ERGs) which were strongly linked to EC cases' outcomes. Then, we detected and confirmed the predictive ability of our proposed classifier via a host of statistical methods, including survival analysis and ROC method. In addition, immune-associated algorithm was implemented to analyze the immune activity of EC samples.

**Results:**

Four EGRs (BCAP31, HSPD1, PDHA1, and UBE2D1) were selected to build an EGR-related classifier (ERC). This classifier could distinguish the patients into different risky subgroups. The remarkable differences in patient outcome between the two groups were observed, and similar results were also confirmed in GEO cohort. In terms of the immune analysis, the ERC could forecast the infiltration level of immunocytes, such as Tregs and NK cells.

**Conclusion:**

We created a four-ERG risk classifier which displays the powerful capability of survival evaluation for EC cases.

## 1. Introduction

Esophageal cancer (EC) is a malignant neoplasm originating from the digestive system in humans with a growing incidence rate [1]. Since the early symptom of EC is not obvious, many cases are diagnosed at a late stage, missing the optimal opportunity for treatment and leading to a dismal prognosis [2]. With the evolution of precision medicine, molecular targeted therapy has become an increasingly valuable research direction for tumor management. Therefore, it is urgent to develop favorable biomarkers for prognosis prediction in EC.

Protein metabolism is the basic process of biological activities. The endoplasmic reticulum is the “largest processing plant,” which can precisely control the whole process of protein transport and process cellular signals [3]. Numerous abnormal cellular states, such as glucose starvation, intracellular calcium abnormalities, disrupted glycosylation modifications, and redox disorders, can disrupt ER function and induce endoplasmic reticulum stress (ER stress) [4]. It could evoke three different effects, namely, the unfolded protein response (UPR), the endoplasmic reticulum-overload response (EOR), and the sterol regulatory cascade response. Both the UPR and EOR can be triggered through the aggregation of unfolded proteins [5]. Moreover, EOR can also attribute to the abnormal accumulation of normal proteins. The sterol cascade regulatory response is caused by depletion of cholesterol synthesized on the surface of the endoplasmic reticulum [6].

Under nonstressed state of ER, GRP78 on the ER membrane binds to three transmembrane proteins, including ATF6, PERK, and IRE1. In response to abnormal environmental stimuli that induce ER stress in cells, GRP78 separates from these transmembrane proteins and activates the corresponding target genes, resulting in a series of pathological responses [7]. During ER stress, PERK could detach from BIP/GRP78 and interact with eIF2, which in turn enhances the phosphorylation of eIF2*α* [8]. Phosphorylated eIF2*α* promotes the expression of ATF4 mRNA and upregulates CHOP at the protein level [9]. Also, IRE1*α* activates caspase4, caspase12, ASK1, and JNK to induce cell apoptosis [10].

With ER stress, cancer cells produce a heat-resistant cytokine that affects the immune cells infiltrating the tumor, which in turn modify the local immune properties and facilitate tumor viability [11]. Mounting studies have shown that the regulation of ER stress is closely bound up with the growth, metastasis, and recurrence of various tumors [12]. Furthermore, ER stress-related genes (ERGs) are proved to display a central part role in the aggravation of cancer. In lung cancer, USP35 could regulate apoptosis triggered by ER stress through stabilization of RRBP1 [13]. DDX5, also named P68, is required for EC suppression in an ER stress relevant way [14]. In addition, Li et al. reported that RSK2 could inhibit the ER stress resistance of breast cancer by blocking cellular autophagy [15]. Despite a growing number of studies suggesting the crucial effect of ER stress in malignancy development, it has not been adequately studied in EC.

Currently, prognostic model based on multiple biomarkers in EC has gained growing interest owing to their robust forecasting reliability [16–18]. Nevertheless, prognostic classifier according to ERGs has not been developed in EC. In this project, we applied gene matrix of EC samples to create a risk classifier to forecast outcome of EC case. In the future, personalized treatment for the EC patients will benefit from our proposed classifier tool.

## 2. Methods

### 2.1. Data Acquisition

The gene matrix and clinical features of two EC sets (TCGA-ESCA and GSE53625) were collected from the public websites: TCGA and GEO. Each sample in the two EC cohorts with follow-up time < 30 days was eliminated. Then, we included 1350 ERGs with relevance score > 5 from GeneCards (Supplementary Table S1). Differentially expressed genes (DEGs) in EC tissues and normal controls were analyzed by the R software “limma” package (∣log_2_ (FC) | = 0.5 and *p* < 0.05) [19]. Next, differentially expressed ERGs (DEERGs) were obtained by interacting with the list of DEGs.

### 2.2. Development of ER Stress-Related Classifier

Firstly, TCGA cohort was selected as the discovery set to determine prognostic ERSRGs via univariate analysis. Next, candidate model genes were enrolled into LASSO regression designed to minimize the overfitting impact of the signature. Finally, we employed multivariate analysis to create the ER stress-related classifier (ERC). The ERC equation was as follows: risk factor = ∑_*i*=1_^*n*^(Coef_*i*_ × ERSRG_*i*_). In this formula, Coef is the coefficient of the ERC generated by Cox analyses.

### 2.3. Survival Analysis and Determination of a Nomogram

The outcome of EC cases was compared between two groups by the Kaplan-Meier (KM) method. The area under the curve (AUC) generated by ROC analysis was applied to detect the accuracy of the ERC. In addition, univariate and multivariate methods were applied to determine the independence of the ERC. Furthermore, we set up a nomogram to reinforce the predictive capacity of the ERC based on various clinical traits. Verification of the nomogram was assessed via calibration curve.

### 2.4. Verification of ERC Markers by the HPA Database

Human Protein Atlas (HPA) is a database which could offer various proteomics data in clinical specimens by immunohistochemistry (IHC) [20]. We confirmed the expression of model genes at protein level between EC and normal control.

### 2.5. Gene Ontology (GO) and Gene Set Enrichment Analysis (GSEA)

GO annotation was instrumental in exploring the biological function of ERSRGs by package “clusterProfiler.” We employed GSEA to identify the tumor hallmarks between two subgroups (*p* < 0.05 and FDR < 0.25). Hallmark gene set (h.all.v7.5.symbols.gmt) was collected from the MSigDB website [21].

### 2.6. Tumor Immune Microenvironment Analysis

To characterize the immune landscape of the EC samples, we conducted single sample gene set enrichment analysis (ssGSEA). It is an algorithm determining the immunocyte infiltration and immune function activity via the normalized enrichment score (NES).

### 2.7. Exploration of the Clinical Potency of the Prognostic Classifier

The mRNAsi is a measure of stem cell properties of tumor calculated from mRNA matrix data and has a positive correlation with stem cell properties. Additionally, we also detect the correlation between microsatellite instability (MSI) and two groups.

## 3. Results

### 3.1. Characterization of Differentially Expressed ERSRGs

**Figure 1:**
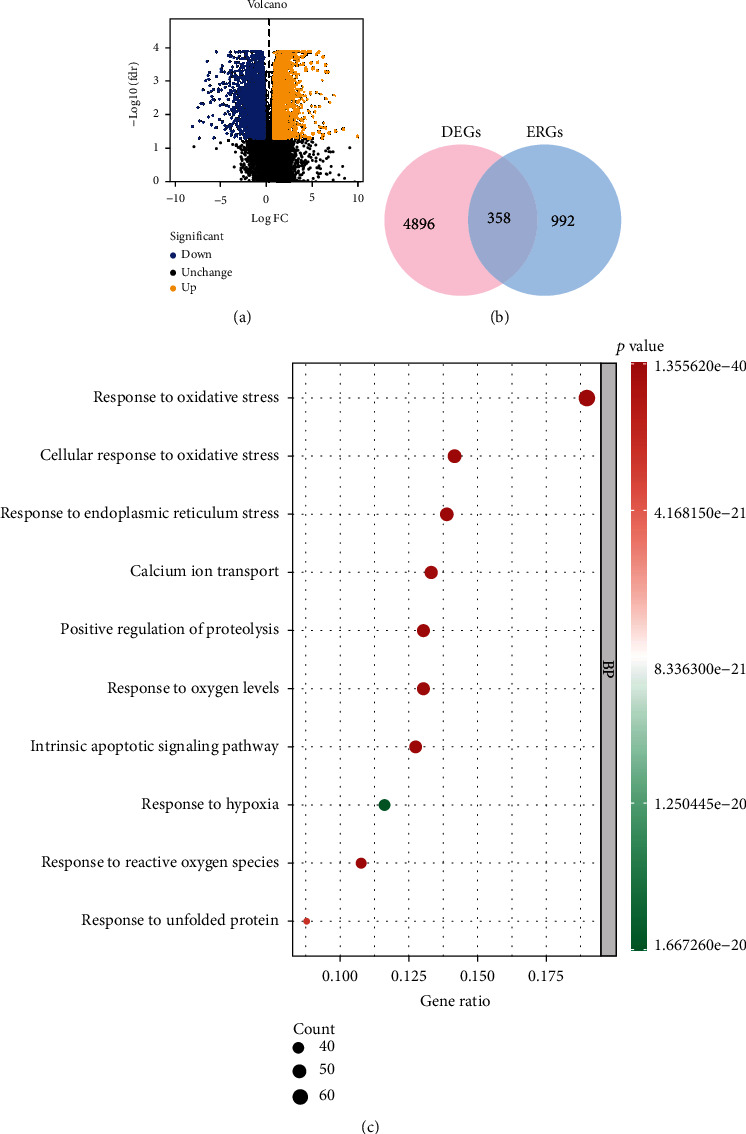
Characterization of ERGs in EC. (a) Volcano plot of DEGs in EC. (b) Venn plot of DEGs and ERGs. (c) GO enrichment analysis.

Performing analysis of the EC dataset by differential expression method, a total of 5254 DEGs were obtained in EC specimen compared with normal counterparts (Figure 1(a)). Next, we screened 358 DEERGs by interacting with 5254 DEGs. Moreover, the function of 358 ERGs was annotated based on GO terms. We found that these genes were associated with oxidative stress, endoplasmic reticulum stress, and calcium ion transport (Figure 1(c)).

### 3.2. Construction and Verification of the ERC

**Figure 2:**
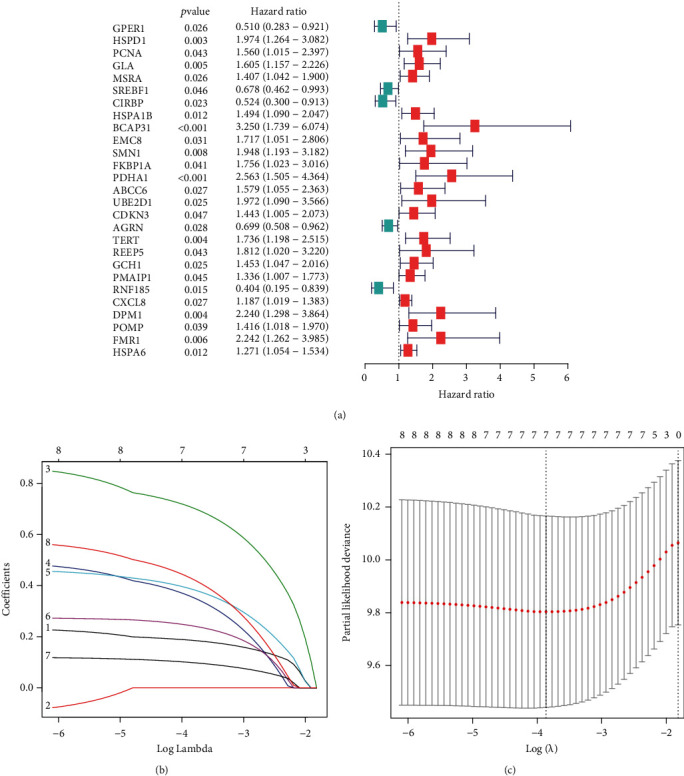
Development of risk classifier based on ERGs. (a) Univariate Cox proportional hazard regression. (b, c) LASSO regression method according to patients' survival.

We first applied the univariate model in the discovery cohort to determine 27 candidate ERGs greatly associated with patient outcome (Figure 2(a)). Next, LASSO analysis was conducted to remove overfitting genes of the model (Figures 2(b) and 2(c)). Untimely, we identified four hub ERGs (BCAP31, HSPD1, PDHA1, and UBE2D1) to build the ERC (Table 1). An ERC-based equation was generated as follows: risk score = (0.8036 × BCAP31) + (0.5864 × HSPD1) + (0.7123 × PDHA1) + (0.5768 × UBE2D1). Selecting the median risk score as the threshold, all the EC cases were classified into high- and low-risk groups.

**Figure 3:**
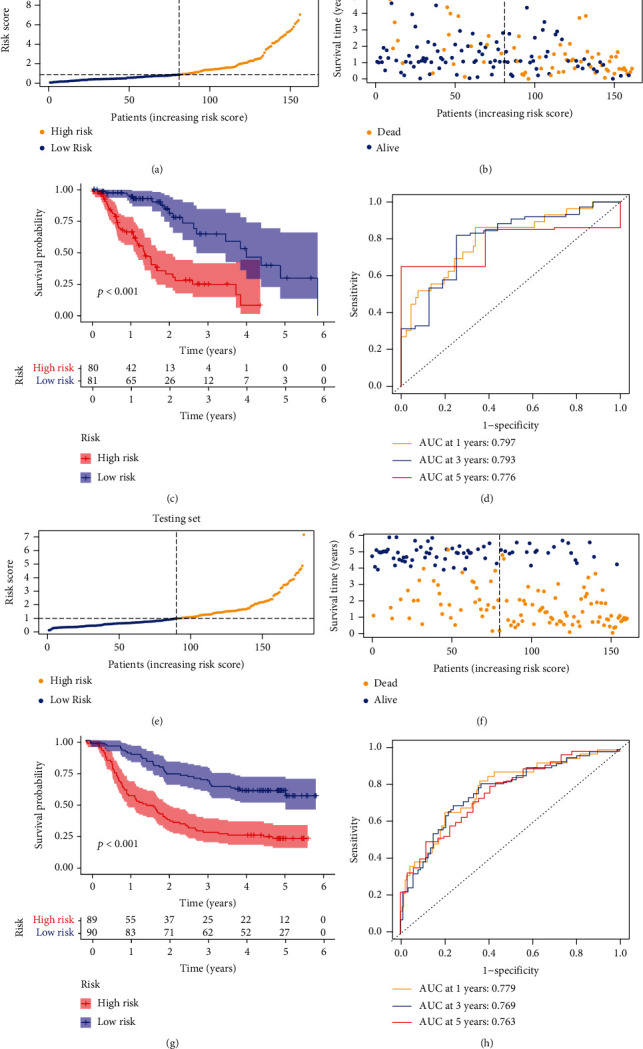
Performance of ERG-based classifier (ERC). (a, b) Layout of risk scores and survival status in TCGA set. (c) Survival analysis of the ERC in TCGA set. (d) ROC analysis of the ERC in TCGA set. (e–h) Similar results verified in GSE53625 external set.

Figure 3 suggests the predictive value of the ERC in terms of clinical outcome in EC cases. In discovery cohort, survival curves unearthed patients with low risk presented a favorable outcome (Figure 3(c)). Further ROC analysis indicated the high AUC for 1-, 3-, and 5-year survival rate (0.797, 0.793, and 0.776, respectively, Figure 3(d)). Meanwhile, we observed similar findings in verification cohort (GSE53625) by the same analyses (Figures 3(e)–3(h)).

### 3.3. Exploration of the Prognostic Values and Protein Expressions of the ERC

**Figure 4:**
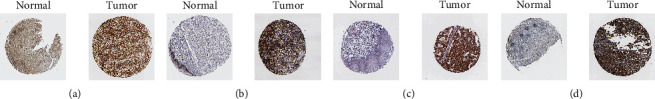
Exploration of the protein expressions of the ERC: (a) BCAP31; (b) HSPD1; (c) PDHA1; (d) UBE2D1.

On the basis of the HPA online tool, the expression patterns of ERGs were confirmed via IHC. We observed that all model genes were highly expressed in EC specimens (Figures 4(a)–4(d)).

### 3.4. Development of a Prognostic Nomogram

**Figure 5:**
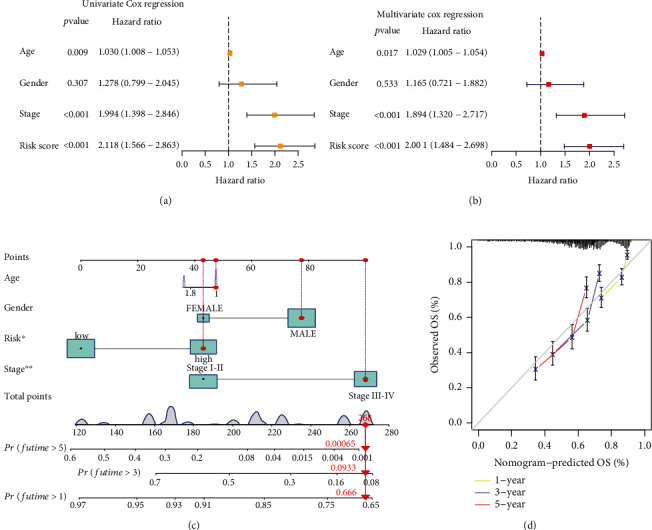
Development of a prognostic nomogram. (a, b) Independent prognosis analysis of the ERC. (c) Construction of a nomogram (^∗^*p* < 0.05; ^∗∗^*p* < 0.01; ^∗∗∗^*p* < 0.001). (d) Calibration analysis presenting robust reliability of the nomogram.

Following the univariate and multivariate methods, we found the risk score of the ERC had a favorable independence of prognosis. In the univariate regression, high ERC score was greatly associated with dismal outcome of cases with EC (Figure 5(a)). Multivariate method revealed that ERC could be an independent indicator for forecasting survival in EC (Figure 5(b)). Moreover, we selected the clinical variables and ERC to set up a nomogram which in turn generated a value for each case. The lower the case value is, the better the patient outcome (Figure 5(c)). The calibration curves uncover a satisfying reliability of the classifier-based nomogram (Figure 5(d)).

### 3.5. GSEA Determines ERC-Associated Hallmarks

**Figure 6:**
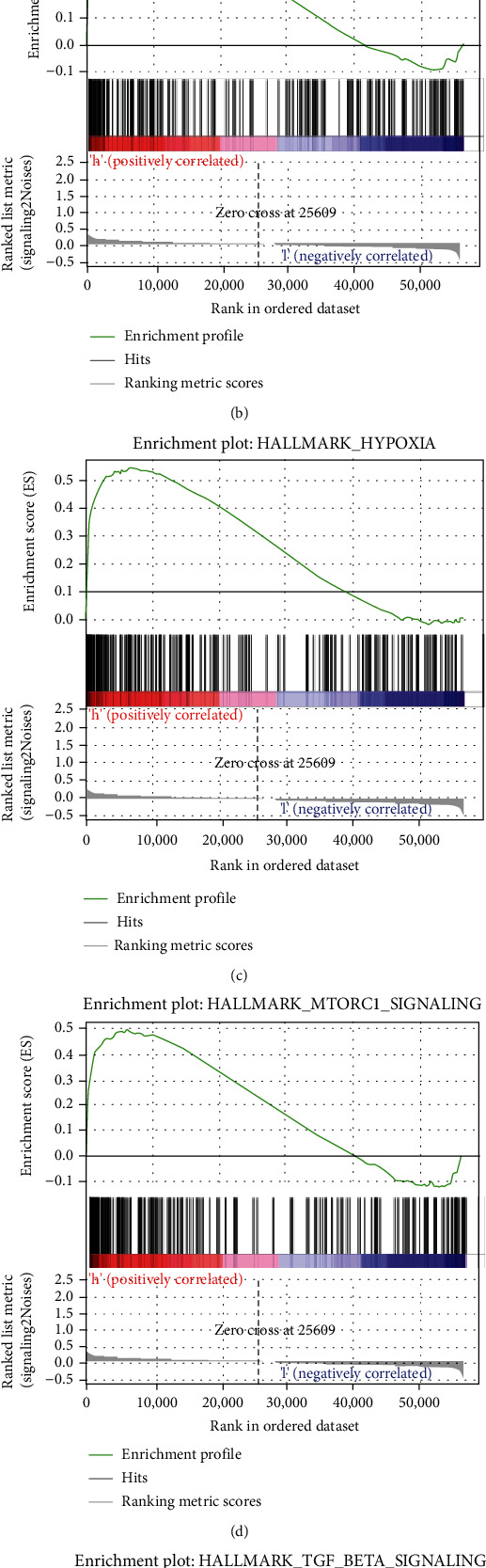
GSEA determines ERC-associated hallmarks: (a) epithelial-mesenchymal transition; (b) glycolysis; (c) hypoxia; (d) MTORC1 pathway; (e) TNF-beta signaling.

The results of GSEA presented five specific hallmarks of ERC, including “epithelial-mesenchymal transition,” “glycolysis,” “hypoxia,” “MTOR pathway,” and “TNF-beta signaling” (Figure 6).

### 3.6. Immune Activity Analysis

**Figure 7:**
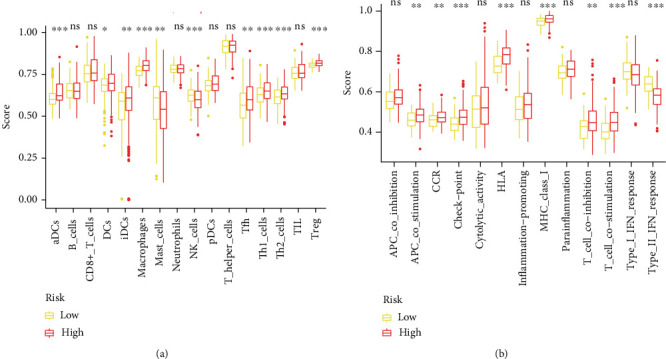
Immune activity analysis. (a) The immunocyte infiltration differences between two groups. (b) The immune function differences between two groups (^∗^*p* < 0.05; ^∗∗^*p* < 0.01; ^∗∗∗^*p* < 0.001).

In order to mirror the immune status of two groups, we estimated enrichment value of different immunocytes and immune function. As shown in Figure 7(a), dendritic cells (DCs), macrophages, and Treg were mainly enriched in ERC-high cohort, while mast cells and NK cells were downregulated in ERC-low cohort. In addition, there were eight immune functions dramatically enriched and only IFN-II response had higher activity in ERC-low cohort (Figure 7(b)).

### 3.7. Exploration of the Clinical Potency of the Prognostic Classifier

**Figure 8:**
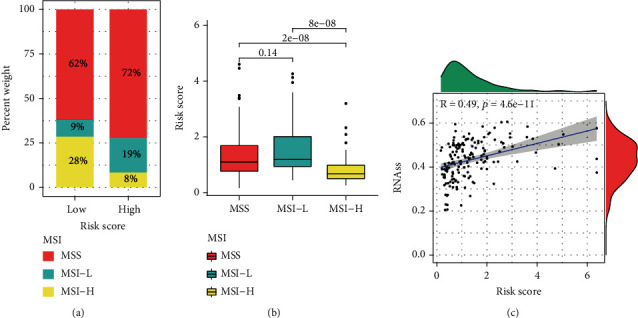
Exploration of the clinical potency of the ERC. (a, b) The MSI infiltration differences between two groups. (c) Cancer stemness feature analysis.

Given the importance of MSI in predicting prognosis for patients with EC, we observed MSS had a higher proportion in ERC-high cohort, suggesting patients with high risk tend to have dismal clinical outcome (Figures 8(a) and 8(b)). In addition, the mRNAsi score tends to grow as risk score increases, pointing out that ERC-high cases might have higher cancer stemness.

## 4. Discussion

EC is a type of uncontrollable tumor originating in the digestive system with dismal patient outcome [1]. Consequently, exploiting novel prognostic indicators and therapeutic approaches is particularly crucial. Numerous researchers have made efforts on biomarker-based classifier and obtained encouraging achievements in assessing the survival outcome of EC [22, 23]. Nevertheless, these prognostic models are more or less flawed and we need to discover more powerful signature for prognosis prediction. Accumulating evidence reveals that ER stress is connected to EC malignant behaviors and treatment failure. As uncovered by Liu et al., IFI6 could affect aggressive cell phenotype of EC by mediating ROC accumulation in an ER stress way [24]. Also, Pang et al. indicated that ER stress triggered by tunicamycin boosts the radiosensitivity of EC [25]. However, the underlying effects of ER stress in EC need more comprehensive analyses. In our project, we tried to develop an ERG-based classifier to enhance risk prediction for EC patients.

In the current research, we successfully set up a risk classifier according to four ERGs (BCAP31, HSPD1, PDHA1, and UBE2D1) for survival outcome evaluation in EC. Our proposed ERC was proved to show promising independence in terms of the prognosis of patients. Furthermore, KM curves illustrated that ERC can accurately categorize the patient's risk classification in EC. At the same time, verification set (GSE53625) was applied to test the features of the ERC. Then, we unearthed a nomogram to exploit the forecasting potential of the classifier through combination of risk score and a host of clinical variables. The potency of our established nomogram was confirmed by calibration plots.

Our nominated ERC was composed of four ERGs which were all risky indicators for prognosis in EC. After checking the previous literatures, we found both four model genes are tightly bound up with various malignancies.

BCAP31, a member of the BCAP family, is shown to be correlated with ER membrane and exerts a crucial role in enhanced cellular adhesion. Accumulating experimental data indicates the oncogenic effects of BCAP31 in numerous tumors. In breast cancer, BCAP31 could foster BC cell aggressive behaviors by binding with EGFR and in turn promoting downstream pathways [26]. Dang et al. reported that inhibiting BCAP31 could block the cell viability and metastasis, suggesting the potential carcinogenic role in cervical cancer [27]. As a target mRNA of miR-451a, BCAP31 may be a barrier to colorectal cancer development through activation of ER stress [28]. PDHA1 is the E1 subunit of PDHc which mediates glycolysis and the tricarboxylic acid cycle [29]. Li et al. observed that PGC1*α* could boost cholangiocarcinoma migration through regulation of PDHA1 expression [30]. In EC, silencing PDHA1 could facilitate cell growth and migration via metabolic reprogramming effect [31]. In addition, Liu and his colleagues suggested that PDHA1 participates in the activation of glycolysis and fosters cancer cell viability by binding with miR-21-5p [32]. HSPD1, also named Heat Shock Protein Family D (HSP60), might play a central part in innate immune system. Fan and his team detected the expression pattern of HSPD1 in head and neck cancer specimens and observed that its downregulation was related to dismal patient outcome [33]. Also, HSPD1 was proved to be highly expressed in lung cancer and displays worse patients' outcome. Depletion of HSPD1 could block cancer cell viability in an oxidative phosphorylation manner [34]. Moreover, the poor prognostic value of HSPD1 was confirmed in Kang et al.'s cohort. At the same time, they found that silencing HSPD1 could suppress metastasis of oral carcinoma via upregulation of E-cadherin expression [35]. UBE2D1, also known as UBCH5, belongs to the E2 ubiquitin-conjugating enzyme family. It mainly serves in the ubiquitination of p53 and HIF1alpha by binding with two ubiquitinating enzymes. Zhou et al. demonstrated that higher UBE2D1 expression could drive liver cancer aggravation through inhibition of p53-related ubiquitination [36]. In addition, Li and his team showed that lncRNA HCG11 could reverse the chemotherapy resistance of stomach cancer via the miR-144-3p/UBE2D1 pathway [37].

Enhanced glycolysis is an adaptation of malignant tumor cells to hypoxic microenvironment. Its antiapoptotic effect may render tumors tolerant to radiotherapy and chemotherapy [38]. Hypoxia could induce the increased expression of HK-II, GLUT1, and LDHA at both mRNA and protein levels in EC cells. The disturbance of HIF-1 *α* could block the expression of these three glycolytic enzymes, which may not be corrected by hypoxia [39]. Sawayama et al. demonstrated that silencing GLUT1 could inhibit cell growth and confer cisplatin resistance to EC via phospho-ERK1/2 [40]. Epithelial tumors can acquire the ability of migration and invasion through epithelial mesenchymal transformation (EMT). After EMT, epithelial cells lost their polarity and obtained mesenchymal phenotype [41]. The study showed that downregulation of ASPP2 could significantly promote the EMT process of EC cells, fostering the metastatic ability of esophageal cancer cells [42]. TGF-*β*, an important regulator of EMT, could promote the process of cellular EMT, contributing to the hypermetastases of various tumors [43]. It was reported that TGF-*β* induced LMO1 via the Smad-dependent signaling pathway, which in turn mediates TGF-*β*-induced EMT and plays a central part in controlling the metastasis of cancer [44]. MTOR signaling is generally enriched in numerous tumors, which could regulate cell proliferation and survival. Li and his team found that miR-195 could suppress mediate the viability and apoptosis of EC cells by binding with HMGA2 and weakening the mTOR signaling pathway [45].

According to the immune landscape analysis, we observed that Treg was greatly enriched in the ERC-high group. Treg, an immunosuppressive cell, could block cellular messages stemming from effector T cells by secretion of cytokines [46]. Additionally, Treg suppresses the activity of B cell through the activation of programmed cell death [47]. In effect, the presence of Treg has been shown to forecast dismal prognosis of cases with EC [48]. Moreover, patients in the ERC-high group showed a lower proportion of NK cell. Previous research has reported that immune compromise is the primary cause of high mortality in tumor patients [49]. NK cells depend on the microenvironment of the bone marrow for their maturation and development [50]. NK cell is considered to be the first line of defense in immune surveillance as it kills tumors without antigen sensitization. It has been suggested that the tumor microenvironment is more reflective of the body's immune function than peripheral blood, and therefore, the detection of NK cell infiltration in tumor tissue should be an ideal approach to determine the immune status of the host [51].

Tumor immune escape (TIE) is the major driver of tumor development. Immune checkpoints are pivotal molecules involved in TIE, turning out to be a hot topic of basic and clinical research recently. Immune checkpoints, through receptor-ligand interactions, transmit inhibitory signals to immunosuppressive cells, thereby inhibiting the latter from performing antitumor effects [52]. Studies show that patients with high expression of PD-L1 are a superior population for immunotherapy [53]. Combined with our previous analysis, we speculated that the high-risk group tends to benefit from immunotherapy.

Heterogeneity is the effect of a subpopulation of cells with cancer stem cells (CSC) on the grade structure of tumor. CSC possesses infinite proliferative capacity, involving both symmetric and asymmetric divisions. The former refers to the ability to self-renew, while the latter is spectral limitation which is attributed to the generation of heterogeneous cells [54]. CSC accounts for approximately 0.1% of overall tumor data and is considered a key source of tumor initiation, progression, metastasis, and treatment resistance [55]. Cancer stemness analysis according to our classifier indicated that the mRNAsi score tends to grow as risk score increases, revealing that patients have a higher probability of tumor metastasis and recurrence.

MSI is an increase or loss of repetitive sequences in the genome due to gene duplication errors, resulting in changes in DNA length [56]. MSI was first identified in hereditary nonpolyposis colon cancer and was subsequently found to be expressed to varying degrees in gastric cancer, liver cancer, and EC [57]. Several projects have concluded that the outcome of EC patients with MSI is better than that of those without MSI. The reason may be that MSI occurs under the stimulation of tumor invasion, which mobilizes the body to produce the corresponding resistance mechanism and subsequently has an optimal clinical outcome [58]. In agreement with these findings, our results suggest that MSS had a higher proportion in the ERC-high cohort.

In summary, our project uncovered a four ERG-based risk classifier which might not only exert an essential role in prognosis evaluation for EC cases but also be utilized to assess the immune status and offer promising reference in therapeutic decision-making of patients with EC.

## Figures and Tables

**Table 1:** Multivariate analysis of the four prognostic ERGs in EC.

Gene	Coefficient	Hazard ratio (95% CI)	*p* value
BCAP31	0.8036	2.23 (1.11-4.51)	0.002
HSPD1	0.5864	1.80 (1.07-3.01)	0.002
PDHA1	0.7123	2.04 (1.18-3.53)	0.011
UBE2D1	0.5768	1.78 (0.91-3.46)	0.089

## Data Availability

Public data were analyzed in this project. All data can be collected from TCGA (https://portal.gdc.cancer.gov/) and GEO (https://www.ncbi.nlm.nih.gov/geo/) databases.

## Supplementary Materials

Supplementary Materials Supplementary Table 1 The list of endoplasmic reticulum stress-related genes.
