# Supplementary material for: Endoplasmic Reticulum Stress-Related Four-Biomarker Risk Classifier for Survival Evaluation in Esophageal Cancer
Source: J Oncol. 2022 Mar 18;2022:5860671. doi: 10.1155/2022/5860671 (PMC8956413; doi:10.1155/2022/5860671)
Supplement: Supplementary Materials — Supplementary Table 1 The list of endoplasmic reticulum stress-related genes. [file 5860671.f1.zip › 5860671.f1/Supplementary Table 1.docx]

Supplementary Table 1 The list of endoplasmic reticulum stress-related genes.

| Gene Symbol | Description | Score |
| --- | --- | --- |
| ERN1 | Endoplasmic Reticulum To Nucleus Signaling 1 | 61.67589 |
| HSPA5 | Heat Shock Protein Family A (Hsp70) Member 5 | 61.20959 |
| ATP2A2 | ATPase Sarcoplasmic/Endoplasmic Reticulum Ca2+ Transporting 2 | 52.67031 |
| ATP2A1 | ATPase Sarcoplasmic/Endoplasmic Reticulum Ca2+ Transporting 1 | 50.40529 |
| SERP1 | Stress Associated Endoplasmic Reticulum Protein 1 | 46.38565 |
| ATP2A3 | ATPase Sarcoplasmic/Endoplasmic Reticulum Ca2+ Transporting 3 | 45.22342 |
| XBP1 | X-Box Binding Protein 1 | 44.63555 |
| EIF2AK3 | Eukaryotic Translation Initiation Factor 2 Alpha Kinase 3 | 43.61681 |
| ERP29 | Endoplasmic Reticulum Protein 29 | 43.32138 |
| SERP2 | Stress Associated Endoplasmic Reticulum Protein Family Member 2 | 42.57033 |
| ERP44 | Endoplasmic Reticulum Protein 44 | 42.19085 |
| KDELR1 | KDEL Endoplasmic Reticulum Protein Retention Receptor 1 | 41.6874 |
| OS9 | OS9 Endoplasmic Reticulum Lectin | 40.63673 |
| ERAP1 | Endoplasmic Reticulum Aminopeptidase 1 | 40.45379 |
| ERO1A | Endoplasmic Reticulum Oxidoreductase 1 Alpha | 40.39962 |
| KDELR2 | KDEL Endoplasmic Reticulum Protein Retention Receptor 2 | 39.99511 |
| HERPUD1 | Homocysteine Inducible ER Protein With Ubiquitin Like Domain 1 | 39.4991 |
| ERLEC1 | Endoplasmic Reticulum Lectin 1 | 37.66689 |
| VCP | Valosin Containing Protein | 37.63148 |
| ERAP2 | Endoplasmic Reticulum Aminopeptidase 2 | 37.29198 |
| KDELR3 | KDEL Endoplasmic Reticulum Protein Retention Receptor 3 | 36.65843 |
| SEC16A | SEC16 Homolog A, Endoplasmic Reticulum Export Factor | 35.70557 |
| ERN2 | Endoplasmic Reticulum To Nucleus Signaling 2 | 35.27291 |
| ATF6 | Activating Transcription Factor 6 | 35.14064 |
| ERP27 | Endoplasmic Reticulum Protein 27 | 34.71851 |
| RER1 | Retention In Endoplasmic Reticulum Sorting Receptor 1 | 34.15969 |
| ERO1B | Endoplasmic Reticulum Oxidoreductase 1 Beta | 33.47313 |
| DDIT3 | DNA Damage Inducible Transcript 3 | 32.97501 |
| CALR | Calreticulin | 32.22462 |
| CHERP | Calcium Homeostasis Endoplasmic Reticulum Protein | 31.32324 |
| TP53 | Tumor Protein P53 | 30.86179 |
| RYR2 | Ryanodine Receptor 2 | 30.58464 |
| HSP90B1 | Heat Shock Protein 90 Beta Family Member 1 | 30.13174 |
| ERMP1 | Endoplasmic Reticulum Metallopeptidase 1 | 29.4426 |
| CANX | Calnexin | 29.07183 |
| EIF2S1 | Eukaryotic Translation Initiation Factor 2 Subunit Alpha | 28.43548 |
| ATF4 | Activating Transcription Factor 4 | 28.40055 |
| RYR1 | Ryanodine Receptor 1 | 27.90683 |
| CPT2 | Carnitine Palmitoyltransferase 2 | 27.30067 |
| TRDN | Triadin | 26.14801 |
| SYVN1 | Synoviolin 1 | 25.99647 |
| PDIA3 | Protein Disulfide Isomerase Family A Member 3 | 25.82389 |
| CASQ2 | Calsequestrin 2 | 25.25162 |
| NFE2L1 | NFE2 Like BZIP Transcription Factor 1 | 25.09475 |
| MAPK8 | Mitogen-Activated Protein Kinase 8 | 24.22639 |
| DERL2 | Derlin 2 | 23.47571 |
| NFE2L2 | NFE2 Like BZIP Transcription Factor 2 | 23.25203 |
| DERL1 | Derlin 1 | 22.58221 |
| LMAN1 | Lectin, Mannose Binding 1 | 22.41786 |
| DNAJC10 | DnaJ Heat Shock Protein Family (Hsp40) Member C10 | 22.37406 |
| TXNDC12 | Thioredoxin Domain Containing 12 | 22.31909 |
| CASQ1 | Calsequestrin 1 | 22.09608 |
| HMOX1 | Heme Oxygenase 1 | 21.96422 |
| APP | Amyloid Beta Precursor Protein | 21.93855 |
| SOD1 | Superoxide Dismutase 1 | 21.8755 |
| CALM1 | Calmodulin 1 | 21.63652 |
| P4HB | Prolyl 4-Hydroxylase Subunit Beta | 21.49491 |
| PSEN1 | Presenilin 1 | 21.47169 |
| HYOU1 | Hypoxia Up-Regulated 1 | 21.25714 |
| MAPK14 | Mitogen-Activated Protein Kinase 14 | 21.14348 |
| CASP3 | Caspase 3 | 20.54427 |
| ITPR1 | Inositol 1,4,5-Trisphosphate Receptor Type 1 | 20.40857 |
| SIL1 | SIL1 Nucleotide Exchange Factor | 20.35003 |
| CASP4 | Caspase 4 | 20.32967 |
| DNAJB9 | DnaJ Heat Shock Protein Family (Hsp40) Member B9 | 20.32507 |
| AMFR | Autocrine Motility Factor Receptor | 20.20701 |
| PRKN | Parkin RBR E3 Ubiquitin Protein Ligase | 20.15544 |
| CAT | Catalase | 20.08779 |
| MAP3K5 | Mitogen-Activated Protein Kinase Kinase Kinase 5 | 20.07761 |
| MAPK1 | Mitogen-Activated Protein Kinase 1 | 20.02571 |
| BCAP31 | B Cell Receptor Associated Protein 31 | 20.01274 |
| BCL2 | BCL2 Apoptosis Regulator | 19.78052 |
| SEC23A | SEC23 Homolog A, COPII Coat Complex Component | 19.77968 |
| DNAJC3 | DnaJ Heat Shock Protein Family (Hsp40) Member C3 | 19.6843 |
| H6PD | Hexose-6-Phosphate Dehydrogenase/Glucose 1-Dehydrogenase | 19.64792 |
| STIM1 | Stromal Interaction Molecule 1 | 19.45386 |
| TNF | Tumor Necrosis Factor | 19.39718 |
| PDIA4 | Protein Disulfide Isomerase Family A Member 4 | 19.37948 |
| CLU | Clusterin | 19.292 |
| G3BP1 | G3BP Stress Granule Assembly Factor 1 | 19.26591 |
| CREB3 | CAMP Responsive Element Binding Protein 3 | 19.1912 |
| SLC6A4 | Solute Carrier Family 6 Member 4 | 19.05468 |
| TXNDC5 | Thioredoxin Domain Containing 5 | 19.00186 |
| PPP1R15A | Protein Phosphatase 1 Regulatory Subunit 15A | 18.8567 |
| JSRP1 | Junctional Sarcoplasmic Reticulum Protein 1 | 18.78548 |
| DERL3 | Derlin 3 | 18.66446 |
| STIP1 | Stress Induced Phosphoprotein 1 | 18.60493 |
| BAX | BCL2 Associated X, Apoptosis Regulator | 18.5895 |
| INS | Insulin | 18.30263 |
| MAN1B1 | Mannosidase Alpha Class 1B Member 1 | 18.2039 |
| MANF | Mesencephalic Astrocyte Derived Neurotrophic Factor | 17.943 |
| ATF6B | Activating Transcription Factor 6 Beta | 17.93627 |
| LNPK | Lunapark, ER Junction Formation Factor | 17.91348 |
| OXSR1 | Oxidative Stress Responsive Kinase 1 | 17.83235 |
| RTN4 | Reticulon 4 | 17.46556 |
| CRH | Corticotropin Releasing Hormone | 17.39136 |
| EPM2A | EPM2A Glucan Phosphatase, Laforin | 17.21646 |
| CFTR | CF Transmembrane Conductance Regulator | 17.16868 |
| DNAJB11 | DnaJ Heat Shock Protein Family (Hsp40) Member B11 | 17.11733 |
| WFS1 | Wolframin ER Transmembrane Glycoprotein | 17.11353 |
| STING1 | Stimulator Of Interferon Response CGAMP Interactor 1 | 17.09591 |
| FKBP14 | FKBP Prolyl Isomerase 14 | 17.04955 |
| SEL1L | SEL1L Adaptor Subunit Of ERAD E3 Ubiquitin Ligase | 16.94469 |
| CREB3L1 | CAMP Responsive Element Binding Protein 3 Like 1 | 16.71351 |
| HSPA1A | Heat Shock Protein Family A (Hsp70) Member 1A | 16.69065 |
| FOS | Fos Proto-Oncogene, AP-1 Transcription Factor Subunit | 16.63699 |
| PARK7 | Parkinsonism Associated Deglycase | 16.40114 |
| MTOR | Mechanistic Target Of Rapamycin Kinase | 16.28128 |
| ATP13A1 | ATPase 13A1 | 16.22049 |
| HSPA4 | Heat Shock Protein Family A (Hsp70) Member 4 | 16.21702 |
| SELENOS | Selenoprotein S | 16.14998 |
| IL6 | Interleukin 6 | 16.02559 |
| DDRGK1 | DDRGK Domain Containing 1 | 15.97115 |
| MIA2 | MIA SH3 Domain ER Export Factor 2 | 15.94477 |
| LMNA | Lamin A/C | 15.94096 |
| KCNQ1 | Potassium Voltage-Gated Channel Subfamily Q Member 1 | 15.88764 |
| KCNH2 | Potassium Voltage-Gated Channel Subfamily H Member 2 | 15.87576 |
| HSF1 | Heat Shock Transcription Factor 1 | 15.86728 |
| SIRT1 | Sirtuin 1 | 15.81215 |
| UBC | Ubiquitin C | 15.58262 |
| ERGIC3 | ERGIC And Golgi 3 | 15.49415 |
| BDNF | Brain Derived Neurotrophic Factor | 15.33601 |
| SEC61A1 | SEC61 Translocon Subunit Alpha 1 | 15.28462 |
| SREBF1 | Sterol Regulatory Element Binding Transcription Factor 1 | 15.26151 |
| SIGMAR1 | Sigma Non-Opioid Intracellular Receptor 1 | 15.21339 |
| G3BP2 | G3BP Stress Granule Assembly Factor 2 | 15.11395 |
| RPN1 | Ribophorin I | 14.9468 |
| SELENON | Selenoprotein N | 14.94155 |
| HSPA8 | Heat Shock Protein Family A (Hsp70) Member 8 | 14.92215 |
| PDIA2 | Protein Disulfide Isomerase Family A Member 2 | 14.88027 |
| IL1B | Interleukin 1 Beta | 14.84518 |
| PTPN1 | Protein Tyrosine Phosphatase Non-Receptor Type 1 | 14.81349 |
| CYCS | Cytochrome C, Somatic | 14.75115 |
| HSP90AA1 | Heat Shock Protein 90 Alpha Family Class A Member 1 | 14.74063 |
| ERLIN2 | ER Lipid Raft Associated 2 | 14.64168 |
| BAG6 | BAG Cochaperone 6 | 14.62383 |
| SURF4 | Surfeit 4 | 14.54184 |
| VAPB | VAMP Associated Protein B And C | 14.52928 |
| CXCL8 | C-X-C Motif Chemokine Ligand 8 | 14.45609 |
| INSIG1 | Insulin Induced Gene 1 | 14.43716 |
| GSR | Glutathione-Disulfide Reductase | 14.3992 |
| NHLRC1 | NHL Repeat Containing E3 Ubiquitin Protein Ligase 1 | 14.35578 |
| NOS3 | Nitric Oxide Synthase 3 | 14.32792 |
| NR3C1 | Nuclear Receptor Subfamily 3 Group C Member 1 | 14.2975 |
| PKP2 | Plakophilin 2 | 14.28161 |
| SREBF2 | Sterol Regulatory Element Binding Transcription Factor 2 | 14.24426 |
| HSPB1 | Heat Shock Protein Family B (Small) Member 1 | 14.16895 |
| CREB3L2 | CAMP Responsive Element Binding Protein 3 Like 2 | 14.16421 |
| SELENOK | Selenoprotein K | 14.08746 |
| APOE | Apolipoprotein E | 14.05471 |
| ANK2 | Ankyrin 2 | 13.99543 |
| DDX3X | DEAD-Box Helicase 3 X-Linked | 13.88916 |
| JUN | Jun Proto-Oncogene, AP-1 Transcription Factor Subunit | 13.79473 |
| SEC31A | SEC31 Homolog A, COPII Coat Complex Component | 13.76338 |
| TMED4 | Transmembrane P24 Trafficking Protein 4 | 13.7511 |
| SCAP | SREBF Chaperone | 13.74349 |
| SEC24A | SEC24 Homolog A, COPII Coat Complex Component | 13.70224 |
| SOD2 | Superoxide Dismutase 2 | 13.69118 |
| MIA3 | MIA SH3 Domain ER Export Factor 3 | 13.67494 |
| HMGCR | 3-Hydroxy-3-Methylglutaryl-CoA Reductase | 13.62906 |
| TGFB1 | Transforming Growth Factor Beta 1 | 13.60651 |
| SNCA | Synuclein Alpha | 13.58748 |
| ADIPOQ | Adiponectin, C1Q And Collagen Domain Containing | 13.56961 |
| RTN3 | Reticulon 3 | 13.54299 |
| TXN | Thioredoxin | 13.51265 |
| SCN5A | Sodium Voltage-Gated Channel Alpha Subunit 5 | 13.50983 |
| EDEM1 | ER Degradation Enhancing Alpha-Mannosidase Like Protein 1 | 13.4877 |
| EIF2AK2 | Eukaryotic Translation Initiation Factor 2 Alpha Kinase 2 | 13.46035 |
| ASPH | Aspartate Beta-Hydroxylase | 13.40934 |
| NOTCH3 | Notch Receptor 3 | 13.36577 |
| RPS27A | Ribosomal Protein S27a | 13.34644 |
| LMAN2 | Lectin, Mannose Binding 2 | 13.3444 |
| BSCL2 | BSCL2 Lipid Droplet Biogenesis Associated, Seipin | 13.32844 |
| CREB3L3 | CAMP Responsive Element Binding Protein 3 Like 3 | 13.31832 |
| SEC13 | SEC13 Homolog, Nuclear Pore And COPII Coat Complex Component | 13.31024 |
| RAB1A | RAB1A, Member RAS Oncogene Family | 13.28846 |
| CKAP4 | Cytoskeleton Associated Protein 4 | 13.27768 |
| PSEN2 | Presenilin 2 | 13.26591 |
| PARP1 | Poly(ADP-Ribose) Polymerase 1 | 13.21008 |
| TRAM1 | Translocation Associated Membrane Protein 1 | 13.20531 |
| PTGS2 | Prostaglandin-Endoperoxide Synthase 2 | 13.19218 |
| ERLIN1 | ER Lipid Raft Associated 1 | 13.17359 |
| TAPBP | TAP Binding Protein | 13.16527 |
| MAPK10 | Mitogen-Activated Protein Kinase 10 | 13.13984 |
| RRBP1 | Ribosome Binding Protein 1 | 13.13486 |
| SQSTM1 | Sequestosome 1 | 13.11823 |
| RAB1B | RAB1B, Member RAS Oncogene Family | 13.11014 |
| ERGIC2 | ERGIC And Golgi 2 | 13.09624 |
| SEC24B | SEC24 Homolog B, COPII Coat Complex Component | 13.0681 |
| CRHR1 | Corticotropin Releasing Hormone Receptor 1 | 13.04762 |
| ESR1 | Estrogen Receptor 1 | 13.0175 |
| CASP9 | Caspase 9 | 13.01397 |
| CLN3 | CLN3 Lysosomal/Endosomal Transmembrane Protein, Battenin | 12.9609 |
| CALM3 | Calmodulin 3 | 12.85714 |
| DMD | Dystrophin | 12.83013 |
| VWF | Von Willebrand Factor | 12.78849 |
| ARL6IP1 | ADP Ribosylation Factor Like GTPase 6 Interacting Protein 1 | 12.77665 |
| TOR1A | Torsin Family 1 Member A | 12.75956 |
| SERPINA1 | Serpin Family A Member 1 | 12.75667 |
| CASP8 | Caspase 8 | 12.75503 |
| UFL1 | UFM1 Specific Ligase 1 | 12.74301 |
| STIM2 | Stromal Interaction Molecule 2 | 12.72384 |
| YIPF5 | Yip1 Domain Family Member 5 | 12.70669 |
| BNIP1 | BCL2 Interacting Protein 1 | 12.69318 |
| P4HTM | Prolyl 4-Hydroxylase, Transmembrane | 12.66548 |
| ALG1 | ALG1 Chitobiosyldiphosphodolichol Beta-Mannosyltransferase | 12.63918 |
| CRP | C-Reactive Protein | 12.61874 |
| ATF3 | Activating Transcription Factor 3 | 12.6033 |
| CAV3 | Caveolin 3 | 12.5933 |
| ATL3 | Atlastin GTPase 3 | 12.5618 |
| MPO | Myeloperoxidase | 12.55774 |
| SESN2 | Sestrin 2 | 12.45641 |
| PPIB | Peptidylprolyl Isomerase B | 12.44719 |
| CISD2 | CDGSH Iron Sulfur Domain 2 | 12.42839 |
| LRRK2 | Leucine Rich Repeat Kinase 2 | 12.40189 |
| TAP1 | Transporter 1, ATP Binding Cassette Subfamily B Member | 12.39668 |
| KEAP1 | Kelch Like ECH Associated Protein 1 | 12.34749 |
| POMC | Proopiomelanocortin | 12.33138 |
| TMED2 | Transmembrane P24 Trafficking Protein 2 | 12.32648 |
| VEGFA | Vascular Endothelial Growth Factor A | 12.25063 |
| AKT1 | AKT Serine/Threonine Kinase 1 | 12.23633 |
| INSIG2 | Insulin Induced Gene 2 | 12.23219 |
| CD4 | CD4 Molecule | 12.1997 |
| PLN | Phospholamban | 12.19842 |
| UGGT1 | UDP-Glucose Glycoprotein Glucosyltransferase 1 | 12.19679 |
| HSPA9 | Heat Shock Protein Family A (Hsp70) Member 9 | 12.14654 |
| JPH2 | Junctophilin 2 | 12.14545 |
| NOX4 | NADPH Oxidase 4 | 12.12688 |
| EIF2AK1 | Eukaryotic Translation Initiation Factor 2 Alpha Kinase 1 | 12.11481 |
| ABL1 | ABL Proto-Oncogene 1, Non-Receptor Tyrosine Kinase | 12.06154 |
| RSAD2 | Radical S-Adenosyl Methionine Domain Containing 2 | 12.02475 |
| APOB | Apolipoprotein B | 12.01772 |
| SAR1B | Secretion Associated Ras Related GTPase 1B | 11.98537 |
| COPB1 | COPI Coat Complex Subunit Beta 1 | 11.95759 |
| SLN | Sarcolipin | 11.9542 |
| CCL2 | C-C Motif Chemokine Ligand 2 | 11.9479 |
| HERPUD2 | HERPUD Family Member 2 | 11.89472 |
| GJA1 | Gap Junction Protein Alpha 1 | 11.89176 |
| SPAST | Spastin | 11.86323 |
| SGK1 | Serum/Glucocorticoid Regulated Kinase 1 | 11.80528 |
| STX17 | Syntaxin 17 | 11.78632 |
| SEC62 | SEC62 Homolog, Preprotein Translocation Factor | 11.75638 |
| CALM2 | Calmodulin 2 | 11.75332 |
| GET3 | Guided Entry Of Tail-Anchored Proteins Factor 3, ATPase | 11.71967 |
| TRIM13 | Tripartite Motif Containing 13 | 11.71597 |
| SEC24C | SEC24 Homolog C, COPII Coat Complex Component | 11.7069 |
| HLA-B | Major Histocompatibility Complex, Class I, B | 11.69621 |
| CACNA1C | Calcium Voltage-Gated Channel Subunit Alpha1 C | 11.69508 |
| ORAI1 | ORAI Calcium Release-Activated Calcium Modulator 1 | 11.67704 |
| DDOST | Dolichyl-Diphosphooligosaccharide--Protein Glycosyltransferase Non-Catalytic Subunit | 11.66921 |
| TMBIM6 | Transmembrane BAX Inhibitor Motif Containing 6 | 11.62822 |
| PRNP | Prion Protein | 11.6281 |
| PDIA6 | Protein Disulfide Isomerase Family A Member 6 | 11.62073 |
| EIF4G1 | Eukaryotic Translation Initiation Factor 4 Gamma 1 | 11.60152 |
| BCL2L1 | BCL2 Like 1 | 11.58755 |
| PRKCD | Protein Kinase C Delta | 11.57875 |
| EGFR | Epidermal Growth Factor Receptor | 11.53666 |
| GBF1 | Golgi Brefeldin A Resistant Guanine Nucleotide Exchange Factor 1 | 11.52339 |
| SLC37A4 | Solute Carrier Family 37 Member 4 | 11.52257 |
| UBE2J1 | Ubiquitin Conjugating Enzyme E2 J1 | 11.51135 |
| PDCD6 | Programmed Cell Death 6 | 11.48717 |
| SEC63 | SEC63 Homolog, Protein Translocation Regulator | 11.48514 |
| SEC61B | SEC61 Translocon Subunit Beta | 11.47974 |
| EDEM2 | ER Degradation Enhancing Alpha-Mannosidase Like Protein 2 | 11.47885 |
| ALB | Albumin | 11.47502 |
| ESYT1 | Extended Synaptotagmin 1 | 11.46611 |
| VIM | Vimentin | 11.45302 |
| CAPN3 | Calpain 3 | 11.43749 |
| TARDBP | TAR DNA Binding Protein | 11.42282 |
| DNAH8 | Dynein Axonemal Heavy Chain 8 | 11.4035 |
| ATL1 | Atlastin GTPase 1 | 11.37472 |
| MYOC | Myocilin | 11.37471 |
| MAPK9 | Mitogen-Activated Protein Kinase 9 | 11.35633 |
| XDH | Xanthine Dehydrogenase | 11.35188 |
| CANT1 | Calcium Activated Nucleotidase 1 | 11.34012 |
| RNF139 | Ring Finger Protein 139 | 11.33491 |
| SERPINH1 | Serpin Family H Member 1 | 11.33261 |
| HTRA2 | HtrA Serine Peptidase 2 | 11.31307 |
| PREB | Prolactin Regulatory Element Binding | 11.30108 |
| OSBPL8 | Oxysterol Binding Protein Like 8 | 11.21776 |
| CAV1 | Caveolin 1 | 11.18445 |
| TAP2 | Transporter 2, ATP Binding Cassette Subfamily B Member | 11.15477 |
| TMED10 | Transmembrane P24 Trafficking Protein 10 | 11.15173 |
| BAK1 | BCL2 Antagonist/Killer 1 | 11.10286 |
| PRDX4 | Peroxiredoxin 4 | 11.0893 |
| TMEM33 | Transmembrane Protein 33 | 11.05001 |
| TFG | Trafficking From ER To Golgi Regulator | 11.04788 |
| SOAT1 | Sterol O-Acyltransferase 1 | 11.04239 |
| SAR1A | Secretion Associated Ras Related GTPase 1A | 11.0314 |
| HSD17B10 | Hydroxysteroid 17-Beta Dehydrogenase 10 | 11.02224 |
| STUB1 | STIP1 Homology And U-Box Containing Protein 1 | 11.01499 |
| EDEM3 | ER Degradation Enhancing Alpha-Mannosidase Like Protein 3 | 11.00724 |
| FOXO1 | Forkhead Box O1 | 11.00432 |
| PINK1 | PTEN Induced Kinase 1 | 10.99677 |
| RYR3 | Ryanodine Receptor 3 | 10.98407 |
| RTN1 | Reticulon 1 | 10.9587 |
| VHL | Von Hippel-Lindau Tumor Suppressor | 10.93574 |
| PRKAA1 | Protein Kinase AMP-Activated Catalytic Subunit Alpha 1 | 10.92647 |
| TEX264 | Testis Expressed 264, ER-Phagy Receptor | 10.91878 |
| VAPA | VAMP Associated Protein A | 10.91279 |
| SEC24D | SEC24 Homolog D, COPII Coat Complex Component | 10.91228 |
| RPN2 | Ribophorin II | 10.91054 |
| CYBA | Cytochrome B-245 Alpha Chain | 10.87929 |
| TRIP11 | Thyroid Hormone Receptor Interactor 11 | 10.87178 |
| BACE1 | Beta-Secretase 1 | 10.84985 |
| TOR1B | Torsin Family 1 Member B | 10.84638 |
| FOXO3 | Forkhead Box O3 | 10.83612 |
| TLR4 | Toll Like Receptor 4 | 10.81847 |
| AUP1 | AUP1 Lipid Droplet Regulating VLDL Assembly Factor | 10.77417 |
| RNF185 | Ring Finger Protein 185 | 10.76441 |
| TECRL | Trans-2,3-Enoyl-CoA Reductase Like | 10.74385 |
| TMED9 | Transmembrane P24 Trafficking Protein 9 | 10.73923 |
| GSK3B | Glycogen Synthase Kinase 3 Beta | 10.73245 |
| HSPA1B | Heat Shock Protein Family A (Hsp70) Member 1B | 10.72249 |
| NFKB1 | Nuclear Factor Kappa B Subunit 1 | 10.71609 |
| UBQLN1 | Ubiquilin 1 | 10.71093 |
| RCN2 | Reticulocalbin 2 | 10.703 |
| TRAF2 | TNF Receptor Associated Factor 2 | 10.67353 |
| MOGS | Mannosyl-Oligosaccharide Glucosidase | 10.66786 |
| ATM | ATM Serine/Threonine Kinase | 10.64906 |
| DHCR24 | 24-Dehydrocholesterol Reductase | 10.64183 |
| PCSK9 | Proprotein Convertase Subtilisin/Kexin Type 9 | 10.63497 |
| NOS2 | Nitric Oxide Synthase 2 | 10.62834 |
| STX18 | Syntaxin 18 | 10.60463 |
| FMR1 | FMRP Translational Regulator 1 | 10.59731 |
| PMM2 | Phosphomannomutase 2 | 10.56867 |
| UGGT2 | UDP-Glucose Glycoprotein Glucosyltransferase 2 | 10.56759 |
| STX5 | Syntaxin 5 | 10.50949 |
| FAF2 | Fas Associated Factor Family Member 2 | 10.50076 |
| AHCYL1 | Adenosylhomocysteinase Like 1 | 10.48951 |
| FKBP5 | FKBP Prolyl Isomerase 5 | 10.48365 |
| TMEM208 | Transmembrane Protein 208 | 10.48354 |
| KCNE1 | Potassium Voltage-Gated Channel Subfamily E Regulatory Subunit 1 | 10.4642 |
| G6PC1 | Glucose-6-Phosphatase Catalytic Subunit 1 | 10.45968 |
| CEBPB | CCAAT Enhancer Binding Protein Beta | 10.45587 |
| MAPK3 | Mitogen-Activated Protein Kinase 3 | 10.45374 |
| UBA52 | Ubiquitin A-52 Residue Ribosomal Protein Fusion Product 1 | 10.44676 |
| TNFRSF10B | TNF Receptor Superfamily Member 10b | 10.44447 |
| CCDC47 | Coiled-Coil Domain Containing 47 | 10.43917 |
| SLC8A1 | Solute Carrier Family 8 Member A1 | 10.43395 |
| TXNIP | Thioredoxin Interacting Protein | 10.42945 |
| IER3IP1 | Immediate Early Response 3 Interacting Protein 1 | 10.38209 |
| CCDC88B | Coiled-Coil Domain Containing 88B | 10.37848 |
| KCNJ5 | Potassium Inwardly Rectifying Channel Subfamily J Member 5 | 10.36464 |
| QRICH1 | Glutamine Rich 1 | 10.36217 |
| EGF | Epidermal Growth Factor | 10.35225 |
| CYP2E1 | Cytochrome P450 Family 2 Subfamily E Member 1 | 10.32307 |
| COMP | Cartilage Oligomeric Matrix Protein | 10.30769 |
| FOXRED2 | FAD Dependent Oxidoreductase Domain Containing 2 | 10.30243 |
| PIEZO1 | Piezo Type Mechanosensitive Ion Channel Component 1 | 10.30134 |
| COMT | Catechol-O-Methyltransferase | 10.28566 |
| BBC3 | BCL2 Binding Component 3 | 10.28216 |
| MAPT | Microtubule Associated Protein Tau | 10.27945 |
| NQO1 | NAD(P)H Quinone Dehydrogenase 1 | 10.25786 |
| RAB10 | RAB10, Member RAS Oncogene Family | 10.2107 |
| G6PD | Glucose-6-Phosphate Dehydrogenase | 10.20141 |
| RAB6A | RAB6A, Member RAS Oncogene Family | 10.19674 |
| NLRP3 | NLR Family Pyrin Domain Containing 3 | 10.19188 |
| PON1 | Paraoxonase 1 | 10.17631 |
| COL7A1 | Collagen Type VII Alpha 1 Chain | 10.16891 |
| HLA-A | Major Histocompatibility Complex, Class I, A | 10.15979 |
| STARD3 | StAR Related Lipid Transfer Domain Containing 3 | 10.15604 |
| BECN1 | Beclin 1 | 10.14536 |
| CYP1A2 | Cytochrome P450 Family 1 Subfamily A Member 2 | 10.14344 |
| LOC110806262 | Solute Carrier Family 6 Member 4 Gene Promoter | 10.14253 |
| EMC1 | ER Membrane Protein Complex Subunit 1 | 10.13597 |
| MAPKAPK2 | MAPK Activated Protein Kinase 2 | 10.11254 |
| COPA | COPI Coat Complex Subunit Alpha | 10.10142 |
| TG | Thyroglobulin | 10.09978 |
| CDKN1A | Cyclin Dependent Kinase Inhibitor 1A | 10.08722 |
| TOR1AIP2 | Torsin 1A Interacting Protein 2 | 10.08272 |
| SEC23IP | SEC23 Interacting Protein | 10.07786 |
| ESYT2 | Extended Synaptotagmin 2 | 10.06518 |
| AQP11 | Aquaporin 11 | 10.05941 |
| SRPRA | SRP Receptor Subunit Alpha | 10.01753 |
| DSP | Desmoplakin | 10.01209 |
| EIF2AK4 | Eukaryotic Translation Initiation Factor 2 Alpha Kinase 4 | 9.963552 |
| KTN1 | Kinectin 1 | 9.935626 |
| FKRP | Fukutin Related Protein | 9.918791 |
| POGLUT2 | Protein O-Glucosyltransferase 2 | 9.914181 |
| GPR37 | G Protein-Coupled Receptor 37 | 9.908205 |
| PRKAA2 | Protein Kinase AMP-Activated Catalytic Subunit Alpha 2 | 9.907925 |
| TMX3 | Thioredoxin Related Transmembrane Protein 3 | 9.863596 |
| GAPDH | Glyceraldehyde-3-Phosphate Dehydrogenase | 9.855058 |
| CYBB | Cytochrome B-245 Beta Chain | 9.835807 |
| THBS1 | Thrombospondin 1 | 9.831228 |
| DNM1L | Dynamin 1 Like | 9.827184 |
| VKORC1 | Vitamin K Epoxide Reductase Complex Subunit 1 | 9.8061 |
| F2 | Coagulation Factor II, Thrombin | 9.805059 |
| SLC2A1 | Solute Carrier Family 2 Member 1 | 9.747637 |
| UBB | Ubiquitin B | 9.731034 |
| HSPD1 | Heat Shock Protein Family D (Hsp60) Member 1 | 9.720814 |
| TRPV4 | Transient Receptor Potential Cation Channel Subfamily V Member 4 | 9.714215 |
| CYB5R3 | Cytochrome B5 Reductase 3 | 9.707078 |
| APEX1 | Apurinic/Apyrimidinic Endodeoxyribonuclease 1 | 9.679354 |
| BCL2L11 | BCL2 Like 11 | 9.677199 |
| MZB1 | Marginal Zone B And B1 Cell Specific Protein | 9.673955 |
| SEC22B | SEC22 Homolog B, Vesicle Trafficking Protein | 9.670108 |
| SHH | Sonic Hedgehog Signaling Molecule | 9.624571 |
| MAP2K6 | Mitogen-Activated Protein Kinase Kinase 6 | 9.623247 |
| CRYAB | Crystallin Alpha B | 9.608627 |
| ATL2 | Atlastin GTPase 2 | 9.607834 |
| CYP1A1 | Cytochrome P450 Family 1 Subfamily A Member 1 | 9.566708 |
| SEC23B | SEC23 Homolog B, COPII Coat Complex Component | 9.552957 |
| KCNE2 | Potassium Voltage-Gated Channel Subfamily E Regulatory Subunit 2 | 9.540857 |
| ZDHHC6 | Zinc Finger DHHC-Type Palmitoyltransferase 6 | 9.530926 |
| DHCR7 | 7-Dehydrocholesterol Reductase | 9.513714 |
| USO1 | USO1 Vesicle Transport Factor | 9.487221 |
| TMX2 | Thioredoxin Related Transmembrane Protein 2 | 9.459623 |
| DMPK | DM1 Protein Kinase | 9.441179 |
| SMPD1 | Sphingomyelin Phosphodiesterase 1 | 9.419294 |
| SRP68 | Signal Recognition Particle 68 | 9.409531 |
| HTT | Huntingtin | 9.403862 |
| SRC | SRC Proto-Oncogene, Non-Receptor Tyrosine Kinase | 9.367157 |
| GPX1 | Glutathione Peroxidase 1 | 9.351853 |
| UBE2G2 | Ubiquitin Conjugating Enzyme E2 G2 | 9.347162 |
| ITPR3 | Inositol 1,4,5-Trisphosphate Receptor Type 3 | 9.343953 |
| HIF1A | Hypoxia Inducible Factor 1 Subunit Alpha | 9.341242 |
| MAP1LC3A | Microtubule Associated Protein 1 Light Chain 3 Alpha | 9.331038 |
| CLN6 | CLN6 Transmembrane ER Protein | 9.330304 |
| GABARAPL1 | GABA Type A Receptor Associated Protein Like 1 | 9.317569 |
| OXT | Oxytocin/Neurophysin I Prepropeptide | 9.307431 |
| OSBP | Oxysterol Binding Protein | 9.29965 |
| MAPK13 | Mitogen-Activated Protein Kinase 13 | 9.298482 |
| GPER1 | G Protein-Coupled Estrogen Receptor 1 | 9.297143 |
| NOTCH1 | Notch Receptor 1 | 9.289087 |
| P3H4 | Prolyl 3-Hydroxylase Family Member 4 (Inactive) | 9.27723 |
| MDM2 | MDM2 Proto-Oncogene | 9.253346 |
| NPC1 | NPC Intracellular Cholesterol Transporter 1 | 9.251797 |
| GBA | Glucosylceramidase Beta | 9.251139 |
| DPAGT1 | Dolichyl-Phosphate N-Acetylglucosaminephosphotransferase 1 | 9.248029 |
| MAP2K7 | Mitogen-Activated Protein Kinase Kinase 7 | 9.23126 |
| UGT1A1 | UDP Glucuronosyltransferase Family 1 Member A1 | 9.218047 |
| NUPR1 | Nuclear Protein 1, Transcriptional Regulator | 9.215899 |
| SCD | Stearoyl-CoA Desaturase | 9.210697 |
| PKD2 | Polycystin 2, Transient Receptor Potential Cation Channel | 9.20929 |
| SCAPER | S-Phase Cyclin A Associated Protein In The ER | 9.193541 |
| ICMT | Isoprenylcysteine Carboxyl Methyltransferase | 9.189874 |
| HRC | Histidine Rich Calcium Binding Protein | 9.180876 |
| LDLR | Low Density Lipoprotein Receptor | 9.178143 |
| F9 | Coagulation Factor IX | 9.156574 |
| BET1 | Bet1 Golgi Vesicular Membrane Trafficking Protein | 9.148979 |
| TMX1 | Thioredoxin Related Transmembrane Protein 1 | 9.141653 |
| CACNA1S | Calcium Voltage-Gated Channel Subunit Alpha1 S | 9.131956 |
| MBTPS1 | Membrane Bound Transcription Factor Peptidase, Site 1 | 9.126532 |
| AGR2 | Anterior Gradient 2, Protein Disulphide Isomerase Family Member | 9.119012 |
| CNIH4 | Cornichon Family AMPA Receptor Auxiliary Protein 4 | 9.111598 |
| S100A1 | S100 Calcium Binding Protein A1 | 9.078587 |
| JPH4 | Junctophilin 4 | 9.068914 |
| CASP7 | Caspase 7 | 9.048899 |
| PPARG | Peroxisome Proliferator Activated Receptor Gamma | 9.030825 |
| PCSK6 | Proprotein Convertase Subtilisin/Kexin Type 6 | 9.01847 |
| BOK | BCL2 Family Apoptosis Regulator BOK | 8.994361 |
| SMPD4 | Sphingomyelin Phosphodiesterase 4 | 8.985379 |
| DRD2 | Dopamine Receptor D2 | 8.970654 |
| UBXN8 | UBX Domain Protein 8 | 8.957863 |
| UGT1A6 | UDP Glucuronosyltransferase Family 1 Member A6 | 8.94973 |
| TMEM214 | Transmembrane Protein 214 | 8.943626 |
| ALG13 | ALG13 UDP-N-Acetylglucosaminyltransferase Subunit | 8.943331 |
| SEC61G | SEC61 Translocon Subunit Gamma | 8.941509 |
| RHOA | Ras Homolog Family Member A | 8.939493 |
| RNFT1 | Ring Finger Protein, Transmembrane 1 | 8.930634 |
| PITPNB | Phosphatidylinositol Transfer Protein Beta | 8.930323 |
| PRDX2 | Peroxiredoxin 2 | 8.927347 |
| STT3A | STT3 Oligosaccharyltransferase Complex Catalytic Subunit A | 8.923197 |
| ARL6IP5 | ADP Ribosylation Factor Like GTPase 6 Interacting Protein 5 | 8.922329 |
| EMC10 | ER Membrane Protein Complex Subunit 10 | 8.897445 |
| CREB1 | CAMP Responsive Element Binding Protein 1 | 8.886888 |
| AKAP9 | A-Kinase Anchoring Protein 9 | 8.878514 |
| PLEKHF2 | Pleckstrin Homology And FYVE Domain Containing 2 | 8.873873 |
| VCAM1 | Vascular Cell Adhesion Molecule 1 | 8.872223 |
| PRDX1 | Peroxiredoxin 1 | 8.87097 |
| VMP1 | Vacuole Membrane Protein 1 | 8.844719 |
| MAOA | Monoamine Oxidase A | 8.833247 |
| PIGN | Phosphatidylinositol Glycan Anchor Biosynthesis Class N | 8.829769 |
| MTTP | Microsomal Triglyceride Transfer Protein | 8.82287 |
| MAP1LC3B | Microtubule Associated Protein 1 Light Chain 3 Beta | 8.819851 |
| EPO | Erythropoietin | 8.80982 |
| BRSK2 | BR Serine/Threonine Kinase 2 | 8.806129 |
| JAK2 | Janus Kinase 2 | 8.800504 |
| STAU1 | Staufen Double-Stranded RNA Binding Protein 1 | 8.794632 |
| MAP2K4 | Mitogen-Activated Protein Kinase Kinase 4 | 8.788831 |
| GABARAPL2 | GABA Type A Receptor Associated Protein Like 2 | 8.78162 |
| INPP5K | Inositol Polyphosphate-5-Phosphatase K | 8.780443 |
| PIK3R1 | Phosphoinositide-3-Kinase Regulatory Subunit 1 | 8.777287 |
| HM13 | Histocompatibility Minor 13 | 8.767171 |
| TMCC1 | Transmembrane And Coiled-Coil Domain Family 1 | 8.762245 |
| ZW10 | Zw10 Kinetochore Protein | 8.760718 |
| DNAJB12 | DnaJ Heat Shock Protein Family (Hsp40) Member B12 | 8.751414 |
| CASP12 | Caspase 12 (Gene/Pseudogene) | 8.749605 |
| RINT1 | RAD50 Interactor 1 | 8.733054 |
| RNF186 | Ring Finger Protein 186 | 8.723569 |
| TRIB3 | Tribbles Pseudokinase 3 | 8.722757 |
| SDHA | Succinate Dehydrogenase Complex Flavoprotein Subunit A | 8.706469 |
| FICD | FIC Domain Protein Adenylyltransferase | 8.703594 |
| UBE2J2 | Ubiquitin Conjugating Enzyme E2 J2 | 8.699154 |
| RAB2A | RAB2A, Member RAS Oncogene Family | 8.690569 |
| LONP1 | Lon Peptidase 1, Mitochondrial | 8.689078 |
| BRCA1 | BRCA1 DNA Repair Associated | 8.68313 |
| SNTA1 | Syntrophin Alpha 1 | 8.681916 |
| HFE | Homeostatic Iron Regulator | 8.678569 |
| MUC1 | Mucin 1, Cell Surface Associated | 8.676291 |
| HSP90AB1 | Heat Shock Protein 90 Alpha Family Class B Member 1 | 8.67391 |
| IL10 | Interleukin 10 | 8.664721 |
| PON2 | Paraoxonase 2 | 8.658606 |
| SDF2L1 | Stromal Cell Derived Factor 2 Like 1 | 8.648069 |
| AIFM1 | Apoptosis Inducing Factor Mitochondria Associated 1 | 8.64011 |
| CAMK2G | Calcium/Calmodulin Dependent Protein Kinase II Gamma | 8.624817 |
| TMED7 | Transmembrane P24 Trafficking Protein 7 | 8.616282 |
| EP300 | E1A Binding Protein P300 | 8.611076 |
| PRDX6 | Peroxiredoxin 6 | 8.604853 |
| OSBPL3 | Oxysterol Binding Protein Like 3 | 8.602255 |
| TUSC3 | Tumor Suppressor Candidate 3 | 8.567719 |
| EDN1 | Endothelin 1 | 8.564335 |
| JPH1 | Junctophilin 1 | 8.550062 |
| MICB | MHC Class I Polypeptide-Related Sequence B | 8.547023 |
| ORMDL3 | ORMDL Sphingolipid Biosynthesis Regulator 3 | 8.533163 |
| C9orf72 | C9orf72-SMCR8 Complex Subunit | 8.510822 |
| TMED1 | Transmembrane P24 Trafficking Protein 1 | 8.500257 |
| ELN | Elastin | 8.493933 |
| GOLGA2 | Golgin A2 | 8.493762 |
| TMTC3 | Transmembrane O-Mannosyltransferase Targeting Cadherins 3 | 8.491985 |
| SHISA5 | Shisa Family Member 5 | 8.49105 |
| TF | Transferrin | 8.484453 |
| EBP | EBP Cholestenol Delta-Isomerase | 8.479836 |
| SLC39A14 | Solute Carrier Family 39 Member 14 | 8.470048 |
| PTEN | Phosphatase And Tensin Homolog | 8.465764 |
| CTNNB1 | Catenin Beta 1 | 8.455572 |
| GSTM1 | Glutathione S-Transferase Mu 1 | 8.452892 |
| UBA5 | Ubiquitin Like Modifier Activating Enzyme 5 | 8.44866 |
| HMGB1 | High Mobility Group Box 1 | 8.446188 |
| IFNG | Interferon Gamma | 8.440768 |
| NR3C2 | Nuclear Receptor Subfamily 3 Group C Member 2 | 8.4305 |
| SCP2 | Sterol Carrier Protein 2 | 8.420654 |
| UBXN4 | UBX Domain Protein 4 | 8.413774 |
| CERT1 | Ceramide Transporter 1 | 8.403917 |
| ATG14 | Autophagy Related 14 | 8.399909 |
| F8 | Coagulation Factor VIII | 8.386883 |
| GRIA1 | Glutamate Ionotropic Receptor AMPA Type Subunit 1 | 8.384485 |
| GABARAP | GABA Type A Receptor-Associated Protein | 8.360096 |
| RCN1 | Reticulocalbin 1 | 8.357349 |
| HSD11B1 | Hydroxysteroid 11-Beta Dehydrogenase 1 | 8.357158 |
| CARD14 | Caspase Recruitment Domain Family Member 14 | 8.353963 |
| AKAP6 | A-Kinase Anchoring Protein 6 | 8.350359 |
| RFT1 | RFT1 Homolog | 8.350307 |
| PIGK | Phosphatidylinositol Glycan Anchor Biosynthesis Class K | 8.338228 |
| GH-LCR | Growth Hormone Locus Control Region | 8.331729 |
| TSPO | Translocator Protein | 8.305192 |
| P3H1 | Prolyl 3-Hydroxylase 1 | 8.296486 |
| POMT2 | Protein O-Mannosyltransferase 2 | 8.291186 |
| ATR | ATR Serine/Threonine Kinase | 8.289417 |
| RAC1 | Rac Family Small GTPase 1 | 8.288699 |
| AGR3 | Anterior Gradient 3, Protein Disulphide Isomerase Family Member | 8.285676 |
| ALG14 | ALG14 UDP-N-Acetylglucosaminyltransferase Subunit | 8.283592 |
| ADRB2 | Adrenoceptor Beta 2 | 8.282927 |
| NCK1 | NCK Adaptor Protein 1 | 8.282869 |
| PTGS1 | Prostaglandin-Endoperoxide Synthase 1 | 8.272216 |
| MPPE1 | Metallophosphoesterase 1 | 8.265921 |
| SP1 | Sp1 Transcription Factor | 8.245627 |
| TMCO1 | Transmembrane And Coiled-Coil Domains 1 | 8.245381 |
| YKT6 | YKT6 V-SNARE Homolog | 8.244467 |
| PPP1R15B | Protein Phosphatase 1 Regulatory Subunit 15B | 8.243247 |
| EEF1A1 | Eukaryotic Translation Elongation Factor 1 Alpha 1 | 8.239214 |
| LBR | Lamin B Receptor | 8.211822 |
| ZC3H12A | Zinc Finger CCCH-Type Containing 12A | 8.210092 |
| NAGLU | N-Acetyl-Alpha-Glucosaminidase | 8.206962 |
| CASP2 | Caspase 2 | 8.205569 |
| MBTPS2 | Membrane Bound Transcription Factor Peptidase, Site 2 | 8.200813 |
| CYP2D6 | Cytochrome P450 Family 2 Subfamily D Member 6 | 8.199604 |
| ITPR2 | Inositol 1,4,5-Trisphosphate Receptor Type 2 | 8.197124 |
| CDK1 | Cyclin Dependent Kinase 1 | 8.189769 |
| CNR1 | Cannabinoid Receptor 1 | 8.18391 |
| FKBP1B | FKBP Prolyl Isomerase 1B | 8.169371 |
| ATP1A3 | ATPase Na+/K+ Transporting Subunit Alpha 3 | 8.161877 |
| INSR | Insulin Receptor | 8.155162 |
| GRIN1 | Glutamate Ionotropic Receptor NMDA Type Subunit 1 | 8.15115 |
| HACD2 | 3-Hydroxyacyl-CoA Dehydratase 2 | 8.151022 |
| GET4 | Guided Entry Of Tail-Anchored Proteins Factor 4 | 8.13945 |
| MCFD2 | Multiple Coagulation Factor Deficiency 2, ER Cargo Receptor Complex Subunit | 8.134626 |
| IGF1 | Insulin Like Growth Factor 1 | 8.126129 |
| GRAMD1A | GRAM Domain Containing 1A | 8.122696 |
| CALHM1 | Calcium Homeostasis Modulator 1 | 8.110737 |
| DBH | Dopamine Beta-Hydroxylase | 8.099258 |
| PIK3C3 | Phosphatidylinositol 3-Kinase Catalytic Subunit Type 3 | 8.081832 |
| SCFD1 | Sec1 Family Domain Containing 1 | 8.074809 |
| SACM1L | SAC1 Like Phosphatidylinositide Phosphatase | 8.068534 |
| GLA | Galactosidase Alpha | 8.067577 |
| ALG2 | ALG2 Alpha-1,3/1,6-Mannosyltransferase | 8.064196 |
| TLR9 | Toll Like Receptor 9 | 8.062104 |
| UBL4A | Ubiquitin Like 4A | 8.058124 |
| GET1 | Guided Entry Of Tail-Anchored Proteins Factor 1 | 8.053599 |
| MYH7 | Myosin Heavy Chain 7 | 8.03334 |
| MAPKAP1 | MAPK Associated Protein 1 | 8.031942 |
| PDZD8 | PDZ Domain Containing 8 | 8.027519 |
| CREBRF | CREB3 Regulatory Factor | 8.025234 |
| SHC1 | SHC Adaptor Protein 1 | 8.016661 |
| ERMARD | ER Membrane Associated RNA Degradation | 8.010435 |
| HMGCLL1 | 3-Hydroxymethyl-3-Methylglutaryl-CoA Lyase Like 1 | 7.999342 |
| UBE2K | Ubiquitin Conjugating Enzyme E2 K | 7.980807 |
| KDR | Kinase Insert Domain Receptor | 7.97788 |
| SELENOF | Selenoprotein F | 7.969396 |
| HSPA13 | Heat Shock Protein Family A (Hsp70) Member 13 | 7.966587 |
| CLN8 | CLN8 Transmembrane ER And ERGIC Protein | 7.963565 |
| LPCAT3 | Lysophosphatidylcholine Acyltransferase 3 | 7.960769 |
| KCNJ11 | Potassium Inwardly Rectifying Channel Subfamily J Member 11 | 7.949436 |
| JKAMP | JNK1/MAPK8 Associated Membrane Protein | 7.944636 |
| DLD | Dihydrolipoamide Dehydrogenase | 7.942204 |
| ZFAND2B | Zinc Finger AN1-Type Containing 2B | 7.921453 |
| BCL2L10 | BCL2 Like 10 | 7.919024 |
| JPH3 | Junctophilin 3 | 7.91677 |
| EEF2 | Eukaryotic Translation Elongation Factor 2 | 7.914554 |
| SEC11A | SEC11 Homolog A, Signal Peptidase Complex Subunit | 7.909825 |
| GPX7 | Glutathione Peroxidase 7 | 7.902972 |
| EMC7 | ER Membrane Protein Complex Subunit 7 | 7.90077 |
| HGSNAT | Heparan-Alpha-Glucosaminide N-Acetyltransferase | 7.898723 |
| PABPC1 | Poly(A) Binding Protein Cytoplasmic 1 | 7.895919 |
| KDSR | 3-Ketodihydrosphingosine Reductase | 7.895583 |
| SGTA | Small Glutamine Rich Tetratricopeptide Repeat Co-Chaperone Alpha | 7.891774 |
| SCAMP5 | Secretory Carrier Membrane Protein 5 | 7.890406 |
| GANAB | Glucosidase II Alpha Subunit | 7.889201 |
| G6PC3 | Glucose-6-Phosphatase Catalytic Subunit 3 | 7.889106 |
| NDRG1 | N-Myc Downstream Regulated 1 | 7.885159 |
| MOSPD2 | Motile Sperm Domain Containing 2 | 7.873013 |
| UFM1 | Ubiquitin Fold Modifier 1 | 7.848809 |
| NR1H2 | Nuclear Receptor Subfamily 1 Group H Member 2 | 7.847532 |
| SOAT2 | Sterol O-Acyltransferase 2 | 7.843211 |
| MAP2K1 | Mitogen-Activated Protein Kinase Kinase 1 | 7.841968 |
| GSTP1 | Glutathione S-Transferase Pi 1 | 7.840194 |
| PRKCA | Protein Kinase C Alpha | 7.837716 |
| UNC93B1 | Unc-93 Homolog B1, TLR Signaling Regulator | 7.836614 |
| YIF1A | Yip1 Interacting Factor Homolog A, Membrane Trafficking Protein | 7.835884 |
| PTPN2 | Protein Tyrosine Phosphatase Non-Receptor Type 2 | 7.827527 |
| SGPP1 | Sphingosine-1-Phosphate Phosphatase 1 | 7.814847 |
| MSRB3 | Methionine Sulfoxide Reductase B3 | 7.804884 |
| CDKN3 | Cyclin Dependent Kinase Inhibitor 3 | 7.804855 |
| KIF1C | Kinesin Family Member 1C | 7.798558 |
| UMOD | Uromodulin | 7.793993 |
| MX1 | MX Dynamin Like GTPase 1 | 7.793513 |
| LACC1 | Laccase Domain Containing 1 | 7.786711 |
| NSF | N-Ethylmaleimide Sensitive Factor, Vesicle Fusing ATPase | 7.786564 |
| MFN2 | Mitofusin 2 | 7.785871 |
| CHAT | Choline O-Acetyltransferase | 7.782014 |
| ACTB | Actin Beta | 7.774903 |
| GORASP1 | Golgi Reassembly Stacking Protein 1 | 7.759888 |
| SCARA3 | Scavenger Receptor Class A Member 3 | 7.751351 |
| TFRC | Transferrin Receptor | 7.750646 |
| CDK5RAP3 | CDK5 Regulatory Subunit Associated Protein 3 | 7.7484 |
| REEP4 | Receptor Accessory Protein 4 | 7.747243 |
| MGST1 | Microsomal Glutathione S-Transferase 1 | 7.740774 |
| GORASP2 | Golgi Reassembly Stacking Protein 2 | 7.734382 |
| PDHA1 | Pyruvate Dehydrogenase E1 Subunit Alpha 1 | 7.731648 |
| PRL | Prolactin | 7.727319 |
| FKBP4 | FKBP Prolyl Isomerase 4 | 7.708244 |
| GRAMD1B | GRAM Domain Containing 1B | 7.705703 |
| AGER | Advanced Glycosylation End-Product Specific Receptor | 7.67233 |
| IL1A | Interleukin 1 Alpha | 7.670585 |
| ANXA5 | Annexin A5 | 7.668034 |
| TECR | Trans-2,3-Enoyl-CoA Reductase | 7.666734 |
| PROC | Protein C, Inactivator Of Coagulation Factors Va And VIIIa | 7.665237 |
| POR | Cytochrome P450 Oxidoreductase | 7.656263 |
| CHRNE | Cholinergic Receptor Nicotinic Epsilon Subunit | 7.654804 |
| NBAS | NBAS Subunit Of NRZ Tethering Complex | 7.644092 |
| SORT1 | Sortilin 1 | 7.643346 |
| REEP1 | Receptor Accessory Protein 1 | 7.641009 |
| CTSD | Cathepsin D | 7.638577 |
| SUMF2 | Sulfatase Modifying Factor 2 | 7.63309 |
| HTR2A | 5-Hydroxytryptamine Receptor 2A | 7.614561 |
| SRP54 | Signal Recognition Particle 54 | 7.600001 |
| FAS | Fas Cell Surface Death Receptor | 7.593858 |
| PRDX3 | Peroxiredoxin 3 | 7.588882 |
| STARD3NL | STARD3 N-Terminal Like | 7.582708 |
| PTGIS | Prostaglandin I2 Synthase | 7.579391 |
| TGM2 | Transglutaminase 2 | 7.578751 |
| DGAT1 | Diacylglycerol O-Acyltransferase 1 | 7.575037 |
| STAT3 | Signal Transducer And Activator Of Transcription 3 | 7.569951 |
| SET | SET Nuclear Proto-Oncogene | 7.566591 |
| KCNJ2 | Potassium Inwardly Rectifying Channel Subfamily J Member 2 | 7.563492 |
| CCND1 | Cyclin D1 | 7.550317 |
| PKD1 | Polycystin 1, Transient Receptor Potential Channel Interacting | 7.545959 |
| HSD17B12 | Hydroxysteroid 17-Beta Dehydrogenase 12 | 7.52845 |
| ANK1 | Ankyrin 1 | 7.525622 |
| TIA1 | TIA1 Cytotoxic Granule Associated RNA Binding Protein | 7.517891 |
| NOTCH2 | Notch Receptor 2 | 7.50958 |
| TMEM259 | Transmembrane Protein 259 | 7.503042 |
| UBAC2 | UBA Domain Containing 2 | 7.49613 |
| ZFYVE27 | Zinc Finger FYVE-Type Containing 27 | 7.493594 |
| CDK5 | Cyclin Dependent Kinase 5 | 7.4925 |
| HAX1 | HCLS1 Associated Protein X-1 | 7.477241 |
| NOL3 | Nucleolar Protein 3 | 7.47268 |
| MICA | MHC Class I Polypeptide-Related Sequence A | 7.470239 |
| MR1 | Major Histocompatibility Complex, Class I-Related | 7.463374 |
| CP | Ceruloplasmin | 7.451293 |
| HLA-C | Major Histocompatibility Complex, Class I, C | 7.445433 |
| PKM | Pyruvate Kinase M1/2 | 7.433251 |
| STT3B | STT3 Oligosaccharyltransferase Complex Catalytic Subunit B | 7.41882 |
| RETREG1 | Reticulophagy Regulator 1 | 7.414987 |
| DPM1 | Dolichyl-Phosphate Mannosyltransferase Subunit 1, Catalytic | 7.407494 |
| RNF183 | Ring Finger Protein 183 | 7.402103 |
| TRPA1 | Transient Receptor Potential Cation Channel Subfamily A Member 1 | 7.40083 |
| SRPRB | SRP Receptor Subunit Beta | 7.398957 |
| DNAJB14 | DnaJ Heat Shock Protein Family (Hsp40) Member B14 | 7.397759 |
| PPM1L | Protein Phosphatase, Mg2+/Mn2+ Dependent 1L | 7.394979 |
| IKBKG | Inhibitor Of Nuclear Factor Kappa B Kinase Regulatory Subunit Gamma | 7.392099 |
| DES | Desmin | 7.392045 |
| CTSB | Cathepsin B | 7.389991 |
| SSR2 | Signal Sequence Receptor Subunit 2 | 7.355722 |
| NGLY1 | N-Glycanase 1 | 7.351737 |
| CLCC1 | Chloride Channel CLIC Like 1 | 7.349089 |
| COPE | COPI Coat Complex Subunit Epsilon | 7.337709 |
| CREBBP | CREB Binding Protein | 7.337461 |
| PPP3CA | Protein Phosphatase 3 Catalytic Subunit Alpha | 7.331773 |
| NOS1 | Nitric Oxide Synthase 1 | 7.327808 |
| PML | PML Nuclear Body Scaffold | 7.325724 |
| MTHFR | Methylenetetrahydrofolate Reductase | 7.323518 |
| CRHR2 | Corticotropin Releasing Hormone Receptor 2 | 7.322817 |
| ICAM1 | Intercellular Adhesion Molecule 1 | 7.321169 |
| MAN1A1 | Mannosidase Alpha Class 1A Member 1 | 7.317418 |
| MAP2K3 | Mitogen-Activated Protein Kinase Kinase 3 | 7.309654 |
| ZFYVE1 | Zinc Finger FYVE-Type Containing 1 | 7.309652 |
| RNF5 | Ring Finger Protein 5 | 7.304906 |
| CYP1B1 | Cytochrome P450 Family 1 Subfamily B Member 1 | 7.303506 |
| PACS2 | Phosphofurin Acidic Cluster Sorting Protein 2 | 7.303095 |
| PIK3CG | Phosphatidylinositol-4,5-Bisphosphate 3-Kinase Catalytic Subunit Gamma | 7.295561 |
| FURIN | Furin, Paired Basic Amino Acid Cleaving Enzyme | 7.291787 |
| ECPAS | Ecm29 Proteasome Adaptor And Scaffold | 7.28675 |
| ELAVL1 | ELAV Like RNA Binding Protein 1 | 7.284131 |
| SYT2 | Synaptotagmin 2 | 7.27916 |
| ABCD1 | ATP Binding Cassette Subfamily D Member 1 | 7.264944 |
| EIF4E | Eukaryotic Translation Initiation Factor 4E | 7.260617 |
| EMC3 | ER Membrane Protein Complex Subunit 3 | 7.258145 |
| TRPM2 | Transient Receptor Potential Cation Channel Subfamily M Member 2 | 7.253201 |
| PITPNM1 | Phosphatidylinositol Transfer Protein Membrane Associated 1 | 7.250624 |
| LEP | Leptin | 7.249093 |
| CLGN | Calmegin | 7.233139 |
| SLC35B1 | Solute Carrier Family 35 Member B1 | 7.214128 |
| F5 | Coagulation Factor V | 7.207996 |
| KRAS | KRAS Proto-Oncogene, GTPase | 7.205581 |
| RHBDD1 | Rhomboid Domain Containing 1 | 7.199158 |
| C1R | Complement C1r | 7.193063 |
| TRAPPC11 | Trafficking Protein Particle Complex Subunit 11 | 7.18701 |
| POMT1 | Protein O-Mannosyltransferase 1 | 7.169538 |
| SSR1 | Signal Sequence Receptor Subunit 1 | 7.168171 |
| DUSP19 | Dual Specificity Phosphatase 19 | 7.167844 |
| GHRL | Ghrelin And Obestatin Prepropeptide | 7.157094 |
| NLRP1 | NLR Family Pyrin Domain Containing 1 | 7.155708 |
| TRAPPC2 | Trafficking Protein Particle Complex Subunit 2 | 7.153577 |
| CYP3A4 | Cytochrome P450 Family 3 Subfamily A Member 4 | 7.149347 |
| SRP72 | Signal Recognition Particle 72 | 7.144242 |
| ZMPSTE24 | Zinc Metallopeptidase STE24 | 7.138841 |
| MYC | MYC Proto-Oncogene, BHLH Transcription Factor | 7.129591 |
| PPARGC1A | PPARG Coactivator 1 Alpha | 7.128012 |
| NPY | Neuropeptide Y | 7.127833 |
| CYP2C19 | Cytochrome P450 Family 2 Subfamily C Member 19 | 7.123262 |
| EMC4 | ER Membrane Protein Complex Subunit 4 | 7.115891 |
| GATA1 | GATA Binding Protein 1 | 7.114007 |
| MGAT2 | Alpha-1,6-Mannosyl-Glycoprotein 2-Beta-N-Acetylglucosaminyltransferase | 7.109999 |
| KPNB1 | Karyopherin Subunit Beta 1 | 7.095943 |
| SEC31B | SEC31 Homolog B, COPII Coat Complex Component | 7.087983 |
| DNAJB2 | DnaJ Heat Shock Protein Family (Hsp40) Member B2 | 7.087176 |
| BTRC | Beta-Transducin Repeat Containing E3 Ubiquitin Protein Ligase | 7.084516 |
| CD74 | CD74 Molecule | 7.079303 |
| BRCA2 | BRCA2 DNA Repair Associated | 7.078496 |
| CLCN1 | Chloride Voltage-Gated Channel 1 | 7.077934 |
| PRDX5 | Peroxiredoxin 5 | 7.075869 |
| ADCYAP1 | Adenylate Cyclase Activating Polypeptide 1 | 7.07373 |
| EIF2B5 | Eukaryotic Translation Initiation Factor 2B Subunit Epsilon | 7.070683 |
| FN1 | Fibronectin 1 | 7.067998 |
| CYB5A | Cytochrome B5 Type A | 7.065848 |
| DSPP | Dentin Sialophosphoprotein | 7.062399 |
| CD36 | CD36 Molecule | 7.054611 |
| TANGO2 | Transport And Golgi Organization 2 Homolog | 7.052702 |
| DST | Dystonin | 7.049953 |
| ATXN2 | Ataxin 2 | 7.042354 |
| STK25 | Serine/Threonine Kinase 25 | 7.042146 |
| MYDGF | Myeloid Derived Growth Factor | 7.04205 |
| PLOD3 | Procollagen-Lysine,2-Oxoglutarate 5-Dioxygenase 3 | 7.038813 |
| TNFRSF1A | TNF Receptor Superfamily Member 1A | 7.036083 |
| KNG1 | Kininogen 1 | 7.031174 |
| SCYL1 | SCY1 Like Pseudokinase 1 | 7.027773 |
| OXTR | Oxytocin Receptor | 7.026897 |
| ABCD4 | ATP Binding Cassette Subfamily D Member 4 | 7.02663 |
| TMEM117 | Transmembrane Protein 117 | 7.020854 |
| RTN2 | Reticulon 2 | 7.017741 |
| CDC42 | Cell Division Cycle 42 | 7.012127 |
| DNAJB1 | DnaJ Heat Shock Protein Family (Hsp40) Member B1 | 7.008397 |
| BCAP29 | B Cell Receptor Associated Protein 29 | 7.004516 |
| EMC2 | ER Membrane Protein Complex Subunit 2 | 7.000575 |
| PLA2G6 | Phospholipase A2 Group VI | 6.997512 |
| S100A9 | S100 Calcium Binding Protein A9 | 6.992437 |
| GBA2 | Glucosylceramidase Beta 2 | 6.982885 |
| CALU | Calumenin | 6.974298 |
| ACTC1 | Actin Alpha Cardiac Muscle 1 | 6.968117 |
| ACTA1 | Actin Alpha 1, Skeletal Muscle | 6.965638 |
| EMC6 | ER Membrane Protein Complex Subunit 6 | 6.954175 |
| GADD45A | Growth Arrest And DNA Damage Inducible Alpha | 6.953204 |
| JAGN1 | Jagunal Homolog 1 | 6.947412 |
| ATF2 | Activating Transcription Factor 2 | 6.92938 |
| MMGT1 | Membrane Magnesium Transporter 1 | 6.925893 |
| FANCD2 | FA Complementation Group D2 | 6.922828 |
| TMEM43 | Transmembrane Protein 43 | 6.91492 |
| COL4A1 | Collagen Type IV Alpha 1 Chain | 6.912389 |
| CYB5R4 | Cytochrome B5 Reductase 4 | 6.911352 |
| PLOD2 | Procollagen-Lysine,2-Oxoglutarate 5-Dioxygenase 2 | 6.908662 |
| TERT | Telomerase Reverse Transcriptase | 6.905632 |
| SSR4 | Signal Sequence Receptor Subunit 4 | 6.901724 |
| VCPIP1 | Valosin Containing Protein Interacting Protein 1 | 6.894663 |
| SLC25A1 | Solute Carrier Family 25 Member 1 | 6.886446 |
| LMBRD1 | LMBR1 Domain Containing 1 | 6.885598 |
| USP19 | Ubiquitin Specific Peptidase 19 | 6.881284 |
| TRAPPC3 | Trafficking Protein Particle Complex Subunit 3 | 6.881111 |
| C1S | Complement C1s | 6.875015 |
| NOS1AP | Nitric Oxide Synthase 1 Adaptor Protein | 6.870567 |
| GOSR1 | Golgi SNAP Receptor Complex Member 1 | 6.868047 |
| HLA-DRA | Major Histocompatibility Complex, Class II, DR Alpha | 6.863194 |
| ELOVL4 | ELOVL Fatty Acid Elongase 4 | 6.860619 |
| SRP14 | Signal Recognition Particle 14 | 6.850735 |
| VAMP7 | Vesicle Associated Membrane Protein 7 | 6.844652 |
| NPLOC4 | NPL4 Homolog, Ubiquitin Recognition Factor | 6.836792 |
| SAMD8 | Sterile Alpha Motif Domain Containing 8 | 6.83396 |
| RNF13 | Ring Finger Protein 13 | 6.833418 |
| SVIP | Small VCP Interacting Protein | 6.829999 |
| REEP5 | Receptor Accessory Protein 5 | 6.820264 |
| YWHAE | Tyrosine 3-Monooxygenase/Tryptophan 5-Monooxygenase Activation Protein Epsilon | 6.817846 |
| MARCHF6 | Membrane Associated Ring-CH-Type Finger 6 | 6.816909 |
| POP1 | POP1 Homolog, Ribonuclease P/MRP Subunit | 6.816401 |
| TTN | Titin | 6.814759 |
| TTR | Transthyretin | 6.812246 |
| TYR | Tyrosinase | 6.80928 |
| SGPP2 | Sphingosine-1-Phosphate Phosphatase 2 | 6.804559 |
| TAPBPL | TAP Binding Protein Like | 6.801363 |
| KCNA2 | Potassium Voltage-Gated Channel Subfamily A Member 2 | 6.801252 |
| CIRBP | Cold Inducible RNA Binding Protein | 6.796681 |
| SLC39A7 | Solute Carrier Family 39 Member 7 | 6.790615 |
| FKBP10 | FKBP Prolyl Isomerase 10 | 6.776898 |
| EMC8 | ER Membrane Protein Complex Subunit 8 | 6.774941 |
| GRIN2A | Glutamate Ionotropic Receptor NMDA Type Subunit 2A | 6.757884 |
| ACSL4 | Acyl-CoA Synthetase Long Chain Family Member 4 | 6.749694 |
| HNRNPK | Heterogeneous Nuclear Ribonucleoprotein K | 6.745708 |
| CPQ | Carboxypeptidase Q | 6.745594 |
| COL2A1 | Collagen Type II Alpha 1 Chain | 6.731063 |
| ADCYAP1R1 | ADCYAP Receptor Type I | 6.730136 |
| RAB18 | RAB18, Member RAS Oncogene Family | 6.725442 |
| SFTPC | Surfactant Protein C | 6.724782 |
| PIGT | Phosphatidylinositol Glycan Anchor Biosynthesis Class T | 6.72053 |
| SRI | Sorcin | 6.714766 |
| CES1 | Carboxylesterase 1 | 6.700781 |
| FKBP1A | FKBP Prolyl Isomerase 1A | 6.694924 |
| SGSH | N-Sulfoglucosamine Sulfohydrolase | 6.686569 |
| IL1RN | Interleukin 1 Receptor Antagonist | 6.67561 |
| DAPK1 | Death Associated Protein Kinase 1 | 6.669888 |
| LAMA2 | Laminin Subunit Alpha 2 | 6.658468 |
| MCL1 | MCL1 Apoptosis Regulator, BCL2 Family Member | 6.657077 |
| SDHB | Succinate Dehydrogenase Complex Iron Sulfur Subunit B | 6.65679 |
| UGT1A9 | UDP Glucuronosyltransferase Family 1 Member A9 | 6.647356 |
| HMOX2 | Heme Oxygenase 2 | 6.62949 |
| PLA2G4C | Phospholipase A2 Group IVC | 6.629183 |
| RET | Ret Proto-Oncogene | 6.620744 |
| FUS | FUS RNA Binding Protein | 6.617366 |
| MAPK8IP1 | Mitogen-Activated Protein Kinase 8 Interacting Protein 1 | 6.615273 |
| NGF | Nerve Growth Factor | 6.609324 |
| RPS6KA3 | Ribosomal Protein S6 Kinase A3 | 6.600644 |
| ACE | Angiotensin I Converting Enzyme | 6.591197 |
| AKR1B1 | Aldo-Keto Reductase Family 1 Member B | 6.590053 |
| HDAC6 | Histone Deacetylase 6 | 6.589203 |
| APOA1 | Apolipoprotein A1 | 6.588792 |
| SOD3 | Superoxide Dismutase 3 | 6.588614 |
| ATP7A | ATPase Copper Transporting Alpha | 6.587573 |
| VRK2 | VRK Serine/Threonine Kinase 2 | 6.58393 |
| RORA | RAR Related Orphan Receptor A | 6.581261 |
| DAXX | Death Domain Associated Protein | 6.575673 |
| F10 | Coagulation Factor X | 6.566853 |
| MLEC | Malectin | 6.560445 |
| COL1A1 | Collagen Type I Alpha 1 Chain | 6.555603 |
| GOSR2 | Golgi SNAP Receptor Complex Member 2 | 6.554127 |
| MMP9 | Matrix Metallopeptidase 9 | 6.550004 |
| NCK2 | NCK Adaptor Protein 2 | 6.54836 |
| CGRRF1 | Cell Growth Regulator With Ring Finger Domain 1 | 6.547925 |
| PLOD1 | Procollagen-Lysine,2-Oxoglutarate 5-Dioxygenase 1 | 6.544441 |
| BCHE | Butyrylcholinesterase | 6.541703 |
| CYP2B6 | Cytochrome P450 Family 2 Subfamily B Member 6 | 6.541309 |
| TXNRD1 | Thioredoxin Reductase 1 | 6.540626 |
| MAOB | Monoamine Oxidase B | 6.538612 |
| BGLAP | Bone Gamma-Carboxyglutamate Protein | 6.535728 |
| HRAS | HRas Proto-Oncogene, GTPase | 6.533372 |
| OPA1 | OPA1 Mitochondrial Dynamin Like GTPase | 6.530684 |
| IGF2BP1 | Insulin Like Growth Factor 2 MRNA Binding Protein 1 | 6.529144 |
| AGTR1 | Angiotensin II Receptor Type 1 | 6.528603 |
| FGFR3 | Fibroblast Growth Factor Receptor 3 | 6.525634 |
| ALPP | Alkaline Phosphatase, Placental | 6.525512 |
| ROCK1 | Rho Associated Coiled-Coil Containing Protein Kinase 1 | 6.517164 |
| UCP2 | Uncoupling Protein 2 | 6.51412 |
| HACD3 | 3-Hydroxyacyl-CoA Dehydratase 3 | 6.511195 |
| IL15RA | Interleukin 15 Receptor Subunit Alpha | 6.507493 |
| CDH1 | Cadherin 1 | 6.499015 |
| OMA1 | OMA1 Zinc Metallopeptidase | 6.498113 |
| GRIN2B | Glutamate Ionotropic Receptor NMDA Type Subunit 2B | 6.493698 |
| FBN1 | Fibrillin 1 | 6.492941 |
| PIK3CA | Phosphatidylinositol-4,5-Bisphosphate 3-Kinase Catalytic Subunit Alpha | 6.489425 |
| USE1 | Unconventional SNARE In The ER 1 | 6.487606 |
| TRPM4 | Transient Receptor Potential Cation Channel Subfamily M Member 4 | 6.486235 |
| CAMLG | Calcium Modulating Ligand | 6.474312 |
| EMC9 | ER Membrane Protein Complex Subunit 9 | 6.473402 |
| EMD | Emerin | 6.471211 |
| ILVBL | IlvB Acetolactate Synthase Like | 6.467979 |
| ACER1 | Alkaline Ceramidase 1 | 6.465769 |
| EXT1 | Exostosin Glycosyltransferase 1 | 6.463466 |
| PTPN11 | Protein Tyrosine Phosphatase Non-Receptor Type 11 | 6.453848 |
| GDF15 | Growth Differentiation Factor 15 | 6.450101 |
| MTDH | Metadherin | 6.446583 |
| VDAC1 | Voltage Dependent Anion Channel 1 | 6.446262 |
| C2CD2L | C2CD2 Like | 6.44539 |
| MMP2 | Matrix Metallopeptidase 2 | 6.445251 |
| TBXAS1 | Thromboxane A Synthase 1 | 6.442664 |
| THBS4 | Thrombospondin 4 | 6.431962 |
| UBQLN2 | Ubiquilin 2 | 6.430781 |
| SCN1A | Sodium Voltage-Gated Channel Alpha Subunit 1 | 6.415355 |
| RMRP | RNA Component Of Mitochondrial RNA Processing Endoribonuclease | 6.403102 |
| NOD2 | Nucleotide Binding Oligomerization Domain Containing 2 | 6.395155 |
| LRPAP1 | LDL Receptor Related Protein Associated Protein 1 | 6.389328 |
| RNF19B | Ring Finger Protein 19B | 6.382182 |
| UGT1A10 | UDP Glucuronosyltransferase Family 1 Member A10 | 6.379853 |
| GLUD1 | Glutamate Dehydrogenase 1 | 6.372826 |
| YWHAZ | Tyrosine 3-Monooxygenase/Tryptophan 5-Monooxygenase Activation Protein Zeta | 6.370739 |
| AGPAT1 | 1-Acylglycerol-3-Phosphate O-Acyltransferase 1 | 6.369559 |
| RELA | RELA Proto-Oncogene, NF-KB Subunit | 6.366933 |
| ALG11 | ALG11 Alpha-1,2-Mannosyltransferase | 6.36377 |
| LMAN1L | Lectin, Mannose Binding 1 Like | 6.356157 |
| FAAH | Fatty Acid Amide Hydrolase | 6.349225 |
| PRKCSH | Protein Kinase C Substrate 80K-H | 6.344691 |
| UFC1 | Ubiquitin-Fold Modifier Conjugating Enzyme 1 | 6.340569 |
| HUWE1 | HECT, UBA And WWE Domain Containing E3 Ubiquitin Protein Ligase 1 | 6.340405 |
| CBY1 | Chibby Family Member 1, Beta Catenin Antagonist | 6.339283 |
| ENTPD5 | Ectonucleoside Triphosphate Diphosphohydrolase 5 (Inactive) | 6.335278 |
| SORL1 | Sortilin Related Receptor 1 | 6.334636 |
| TRAPPC5 | Trafficking Protein Particle Complex Subunit 5 | 6.333853 |
| CREB3L4 | CAMP Responsive Element Binding Protein 3 Like 4 | 6.317355 |
| ATXN2L | Ataxin 2 Like | 6.315902 |
| EEF1B2 | Eukaryotic Translation Elongation Factor 1 Beta 2 | 6.313234 |
| DICER1 | Dicer 1, Ribonuclease III | 6.312777 |
| DAB2IP | DAB2 Interacting Protein | 6.31272 |
| UFD1 | Ubiquitin Recognition Factor In ER Associated Degradation 1 | 6.307011 |
| GOLPH3 | Golgi Phosphoprotein 3 | 6.30382 |
| DYSF | Dysferlin | 6.299904 |
| PPP1CA | Protein Phosphatase 1 Catalytic Subunit Alpha | 6.2995 |
| SPTLC1 | Serine Palmitoyltransferase Long Chain Base Subunit 1 | 6.295683 |
| DCSTAMP | Dendrocyte Expressed Seven Transmembrane Protein | 6.292465 |
| TLR3 | Toll Like Receptor 3 | 6.290827 |
| TNFSF10 | TNF Superfamily Member 10 | 6.289204 |
| NR1H3 | Nuclear Receptor Subfamily 1 Group H Member 3 | 6.288299 |
| ADAMTSL1 | ADAMTS Like 1 | 6.284753 |
| ARFGAP2 | ADP Ribosylation Factor GTPase Activating Protein 2 | 6.28356 |
| SERPINE1 | Serpin Family E Member 1 | 6.27388 |
| AGRN | Agrin | 6.273223 |
| MAP3K7 | Mitogen-Activated Protein Kinase Kinase Kinase 7 | 6.272805 |
| LRP6 | LDL Receptor Related Protein 6 | 6.270723 |
| SERPINC1 | Serpin Family C Member 1 | 6.26637 |
| KPNA2 | Karyopherin Subunit Alpha 2 | 6.26183 |
| UBE2D3 | Ubiquitin Conjugating Enzyme E2 D3 | 6.254593 |
| ACP1 | Acid Phosphatase 1 | 6.25178 |
| B2M | Beta-2-Microglobulin | 6.242842 |
| ELOVL5 | ELOVL Fatty Acid Elongase 5 | 6.241076 |
| G6PC2 | Glucose-6-Phosphatase Catalytic Subunit 2 | 6.240473 |
| SNAP25 | Synaptosome Associated Protein 25 | 6.233238 |
| TEX2 | Testis Expressed 2 | 6.227961 |
| ARSA | Arylsulfatase A | 6.22534 |
| DHDDS | Dehydrodolichyl Diphosphate Synthase Subunit | 6.223619 |
| CHEK1 | Checkpoint Kinase 1 | 6.220944 |
| ATP13A2 | ATPase Cation Transporting 13A2 | 6.220743 |
| PXN | Paxillin | 6.206064 |
| LGALS1 | Galectin 1 | 6.204163 |
| BAG3 | BAG Cochaperone 3 | 6.196301 |
| CAPN2 | Calpain 2 | 6.192875 |
| NAPA | NSF Attachment Protein Alpha | 6.191706 |
| SSR3 | Signal Sequence Receptor Subunit 3 | 6.190781 |
| PDIA5 | Protein Disulfide Isomerase Family A Member 5 | 6.18777 |
| UGT1A8 | UDP Glucuronosyltransferase Family 1 Member A8 | 6.179358 |
| ATP5MK | ATP Synthase Membrane Subunit K | 6.170422 |
| CAMK2A | Calcium/Calmodulin Dependent Protein Kinase II Alpha | 6.169222 |
| GCH1 | GTP Cyclohydrolase 1 | 6.164262 |
| VMA21 | Vacuolar ATPase Assembly Factor VMA21 | 6.154005 |
| NPM1 | Nucleophosmin 1 | 6.148796 |
| GPAT3 | Glycerol-3-Phosphate Acyltransferase 3 | 6.14737 |
| MYH6 | Myosin Heavy Chain 6 | 6.147057 |
| PLD3 | Phospholipase D Family Member 3 | 6.146048 |
| BMP2 | Bone Morphogenetic Protein 2 | 6.144058 |
| BSG | Basigin (Ok Blood Group) | 6.141879 |
| ABCC8 | ATP Binding Cassette Subfamily C Member 8 | 6.139851 |
| PSMD2 | Proteasome 26S Subunit Ubiquitin Receptor, Non-ATPase 2 | 6.133228 |
| ATP2C1 | ATPase Secretory Pathway Ca2+ Transporting 1 | 6.132174 |
| F7 | Coagulation Factor VII | 6.127978 |
| CRAT | Carnitine O-Acetyltransferase | 6.12703 |
| RPA1 | Replication Protein A1 | 6.126224 |
| PSENEN | Presenilin Enhancer, Gamma-Secretase Subunit | 6.126016 |
| RARA | Retinoic Acid Receptor Alpha | 6.116398 |
| NEPRO | Nucleolus And Neural Progenitor Protein | 6.113357 |
| KCNQ2 | Potassium Voltage-Gated Channel Subfamily Q Member 2 | 6.11304 |
| SIRT2 | Sirtuin 2 | 6.105289 |
| DPM3 | Dolichyl-Phosphate Mannosyltransferase Subunit 3, Regulatory | 6.093274 |
| HLA-G | Major Histocompatibility Complex, Class I, G | 6.08999 |
| IL2 | Interleukin 2 | 6.089747 |
| PCNA | Proliferating Cell Nuclear Antigen | 6.08961 |
| EGR1 | Early Growth Response 1 | 6.086165 |
| GPAA1 | Glycosylphosphatidylinositol Anchor Attachment 1 | 6.084425 |
| ABCG1 | ATP Binding Cassette Subfamily G Member 1 | 6.082954 |
| CDIPT | CDP-Diacylglycerol--Inositol 3-Phosphatidyltransferase | 6.082542 |
| TRAPPC9 | Trafficking Protein Particle Complex Subunit 9 | 6.069424 |
| NPPB | Natriuretic Peptide B | 6.064466 |
| PLPP3 | Phospholipid Phosphatase 3 | 6.055777 |
| AFG3L2 | AFG3 Like Matrix AAA Peptidase Subunit 2 | 6.055329 |
| PDLIM1 | PDZ And LIM Domain 1 | 6.049617 |
| PRKD1 | Protein Kinase D1 | 6.041307 |
| HCRT | Hypocretin Neuropeptide Precursor | 6.038782 |
| PIGA | Phosphatidylinositol Glycan Anchor Biosynthesis Class A | 6.032589 |
| RPTOR | Regulatory Associated Protein Of MTOR Complex 1 | 6.03122 |
| SIRT3 | Sirtuin 3 | 6.030943 |
| HSPG2 | Heparan Sulfate Proteoglycan 2 | 6.030023 |
| PIK3R2 | Phosphoinositide-3-Kinase Regulatory Subunit 2 | 6.029199 |
| HSPA6 | Heat Shock Protein Family A (Hsp70) Member 6 | 6.027986 |
| GRP | Gastrin Releasing Peptide | 6.024777 |
| VCL | Vinculin | 6.018586 |
| YIF1B | Yip1 Interacting Factor Homolog B, Membrane Trafficking Protein | 6.017898 |
| CAMK2D | Calcium/Calmodulin Dependent Protein Kinase II Delta | 6.012105 |
| PCK1 | Phosphoenolpyruvate Carboxykinase 1 | 6.010427 |
| DAD1 | Defender Against Cell Death 1 | 6.001939 |
| SLC6A1 | Solute Carrier Family 6 Member 1 | 6.000302 |
| DNAJA1 | DnaJ Heat Shock Protein Family (Hsp40) Member A1 | 5.99088 |
| STAT1 | Signal Transducer And Activator Of Transcription 1 | 5.981032 |
| COPG1 | COPI Coat Complex Subunit Gamma 1 | 5.967433 |
| ANKLE2 | Ankyrin Repeat And LEM Domain Containing 2 | 5.958817 |
| CYP17A1 | Cytochrome P450 Family 17 Subfamily A Member 1 | 5.955261 |
| SLC1A1 | Solute Carrier Family 1 Member 1 | 5.954959 |
| ITGB1 | Integrin Subunit Beta 1 | 5.953186 |
| TREM2 | Triggering Receptor Expressed On Myeloid Cells 2 | 5.951803 |
| ACTG1 | Actin Gamma 1 | 5.949855 |
| VPS33A | VPS33A Core Subunit Of CORVET And HOPS Complexes | 5.945069 |
| SLC37A1 | Solute Carrier Family 37 Member 1 | 5.939048 |
| KCNMA1 | Potassium Calcium-Activated Channel Subfamily M Alpha 1 | 5.937607 |
| FBXO6 | F-Box Protein 6 | 5.932965 |
| ABCA1 | ATP Binding Cassette Subfamily A Member 1 | 5.92979 |
| SELENOT | Selenoprotein T | 5.92809 |
| NOX5 | NADPH Oxidase 5 | 5.926461 |
| TLR2 | Toll Like Receptor 2 | 5.926191 |
| PARP16 | Poly(ADP-Ribose) Polymerase Family Member 16 | 5.915741 |
| COL13A1 | Collagen Type XIII Alpha 1 Chain | 5.913128 |
| BID | BH3 Interacting Domain Death Agonist | 5.90557 |
| LAMP2 | Lysosomal Associated Membrane Protein 2 | 5.901524 |
| GPX8 | Glutathione Peroxidase 8 (Putative) | 5.89755 |
| PLA2G4A | Phospholipase A2 Group IVA | 5.890527 |
| PPARA | Peroxisome Proliferator Activated Receptor Alpha | 5.885859 |
| LGI4 | Leucine Rich Repeat LGI Family Member 4 | 5.881665 |
| YBX1 | Y-Box Binding Protein 1 | 5.880422 |
| MSRA | Methionine Sulfoxide Reductase A | 5.88028 |
| UGT1A7 | UDP Glucuronosyltransferase Family 1 Member A7 | 5.875714 |
| CACNA1A | Calcium Voltage-Gated Channel Subunit Alpha1 A | 5.87571 |
| UQCRFS1 | Ubiquinol-Cytochrome C Reductase, Rieske Iron-Sulfur Polypeptide 1 | 5.875269 |
| FLT3 | Fms Related Receptor Tyrosine Kinase 3 | 5.875015 |
| RAP1GDS1 | Rap1 GTPase-GDP Dissociation Stimulator 1 | 5.870473 |
| WWOX | WW Domain Containing Oxidoreductase | 5.86779 |
| ARSH | Arylsulfatase Family Member H | 5.865429 |
| PIGC | Phosphatidylinositol Glycan Anchor Biosynthesis Class C | 5.861403 |
| ALDH3A2 | Aldehyde Dehydrogenase 3 Family Member A2 | 5.859984 |
| UGT1A4 | UDP Glucuronosyltransferase Family 1 Member A4 | 5.857067 |
| NACA | Nascent Polypeptide Associated Complex Subunit Alpha | 5.855126 |
| ALPL | Alkaline Phosphatase, Biomineralization Associated | 5.854789 |
| MSRB1 | Methionine Sulfoxide Reductase B1 | 5.854691 |
| COLGALT1 | Collagen Beta(1-O)Galactosyltransferase 1 | 5.854337 |
| SLC27A2 | Solute Carrier Family 27 Member 2 | 5.843897 |
| PIGB | Phosphatidylinositol Glycan Anchor Biosynthesis Class B | 5.838052 |
| CUL3 | Cullin 3 | 5.824803 |
| UGT1A3 | UDP Glucuronosyltransferase Family 1 Member A3 | 5.821043 |
| PMEL | Premelanosome Protein | 5.818973 |
| YTHDF2 | YTH N6-Methyladenosine RNA Binding Protein 2 | 5.813521 |
| TRAPPC4 | Trafficking Protein Particle Complex Subunit 4 | 5.807836 |
| TRA | T Cell Receptor Alpha Locus | 5.805613 |
| LPL | Lipoprotein Lipase | 5.804234 |
| KMT2B | Lysine Methyltransferase 2B | 5.800021 |
| SLC4A1 | Solute Carrier Family 4 Member 1 (Diego Blood Group) | 5.798791 |
| UBQLN4 | Ubiquilin 4 | 5.794048 |
| ATP1A1 | ATPase Na+/K+ Transporting Subunit Alpha 1 | 5.793483 |
| TRPV1 | Transient Receptor Potential Cation Channel Subfamily V Member 1 | 5.79318 |
| SETD2 | SET Domain Containing 2, Histone Lysine Methyltransferase | 5.792985 |
| PRKRA | Protein Activator Of Interferon Induced Protein Kinase EIF2AK2 | 5.788273 |
| CYP19A1 | Cytochrome P450 Family 19 Subfamily A Member 1 | 5.787447 |
| LGALS3 | Galectin 3 | 5.785028 |
| POFUT1 | Protein O-Fucosyltransferase 1 | 5.779183 |
| YOD1 | YOD1 Deubiquitinase | 5.775467 |
| TAOK3 | TAO Kinase 3 | 5.774519 |
| PTPRC | Protein Tyrosine Phosphatase Receptor Type C | 5.768814 |
| MIR34A | MicroRNA 34a | 5.76838 |
| DLAT | Dihydrolipoamide S-Acetyltransferase | 5.758927 |
| RPE65 | Retinoid Isomerohydrolase RPE65 | 5.755983 |
| GFAP | Glial Fibrillary Acidic Protein | 5.755078 |
| LRIT3 | Leucine Rich Repeat, Ig-Like And Transmembrane Domains 3 | 5.751523 |
| FAM120A | Family With Sequence Similarity 120A | 5.74563 |
| TRAF6 | TNF Receptor Associated Factor 6 | 5.736246 |
| EPHX1 | Epoxide Hydrolase 1 | 5.728276 |
| UCHL1 | Ubiquitin C-Terminal Hydrolase L1 | 5.711159 |
| UVRAG | UV Radiation Resistance Associated | 5.708395 |
| PLP1 | Proteolipid Protein 1 | 5.704718 |
| CDK2 | Cyclin Dependent Kinase 2 | 5.703929 |
| SI | Sucrase-Isomaltase | 5.701946 |
| PARG | Poly(ADP-Ribose) Glycohydrolase | 5.698216 |
| UBE2D2 | Ubiquitin Conjugating Enzyme E2 D2 | 5.693721 |
| CCDC88A | Coiled-Coil Domain Containing 88A | 5.690186 |
| SLC8A3 | Solute Carrier Family 8 Member A3 | 5.688931 |
| SCN10A | Sodium Voltage-Gated Channel Alpha Subunit 10 | 5.685342 |
| EOGT | EGF Domain Specific O-Linked N-Acetylglucosamine Transferase | 5.684913 |
| EIF5A | Eukaryotic Translation Initiation Factor 5A | 5.683418 |
| NPC2 | NPC Intracellular Cholesterol Transporter 2 | 5.683301 |
| KCNN4 | Potassium Calcium-Activated Channel Subfamily N Member 4 | 5.682719 |
| AGT | Angiotensinogen | 5.682107 |
| ELOVL2 | ELOVL Fatty Acid Elongase 2 | 5.679664 |
| ANXA2 | Annexin A2 | 5.677977 |
| PIGH | Phosphatidylinositol Glycan Anchor Biosynthesis Class H | 5.675057 |
| NSFL1C | NSFL1 Cofactor | 5.6745 |
| TM7SF2 | Transmembrane 7 Superfamily Member 2 | 5.671021 |
| DPM2 | Dolichyl-Phosphate Mannosyltransferase Subunit 2, Regulatory | 5.670761 |
| XIAP | X-Linked Inhibitor Of Apoptosis | 5.665239 |
| TH | Tyrosine Hydroxylase | 5.6622 |
| PIGS | Phosphatidylinositol Glycan Anchor Biosynthesis Class S | 5.657073 |
| BAD | BCL2 Associated Agonist Of Cell Death | 5.644003 |
| PSMA7 | Proteasome 20S Subunit Alpha 7 | 5.641157 |
| RACK1 | Receptor For Activated C Kinase 1 | 5.639627 |
| HTR1A | 5-Hydroxytryptamine Receptor 1A | 5.638844 |
| USP13 | Ubiquitin Specific Peptidase 13 | 5.638432 |
| VAMP1 | Vesicle Associated Membrane Protein 1 | 5.638091 |
| PNKD | PNKD Metallo-Beta-Lactamase Domain Containing | 5.636946 |
| WDR83OS | WD Repeat Domain 83 Opposite Strand | 5.634799 |
| USP9X | Ubiquitin Specific Peptidase 9 X-Linked | 5.634021 |
| H2AX | H2A.X Variant Histone | 5.6283 |
| ADAMTS13 | ADAM Metallopeptidase With Thrombospondin Type 1 Motif 13 | 5.62463 |
| UGT1A | UDP Glucuronosyltransferase Family 1 Member A Complex Locus | 5.623094 |
| CASP1 | Caspase 1 | 5.622622 |
| FITM2 | Fat Storage Inducing Transmembrane Protein 2 | 5.619159 |
| MET | MET Proto-Oncogene, Receptor Tyrosine Kinase | 5.617685 |
| USP14 | Ubiquitin Specific Peptidase 14 | 5.608085 |
| ARF1 | ADP Ribosylation Factor 1 | 5.600962 |
| PPIA | Peptidylprolyl Isomerase A | 5.598885 |
| ASL | Argininosuccinate Lyase | 5.598268 |
| SEC22A | SEC22 Homolog A, Vesicle Trafficking Protein | 5.597146 |
| IL18 | Interleukin 18 | 5.589247 |
| PTPA | Protein Phosphatase 2 Phosphatase Activator | 5.578484 |
| RASGRF2 | Ras Protein Specific Guanine Nucleotide Releasing Factor 2 | 5.570591 |
| TGFA | Transforming Growth Factor Alpha | 5.570003 |
| PPIF | Peptidylprolyl Isomerase F | 5.567487 |
| TRAP1 | TNF Receptor Associated Protein 1 | 5.564984 |
| LRRC59 | Leucine Rich Repeat Containing 59 | 5.561094 |
| HSPA1L | Heat Shock Protein Family A (Hsp70) Member 1 Like | 5.558681 |
| KL | Klotho | 5.54951 |
| RAB3GAP1 | RAB3 GTPase Activating Protein Catalytic Subunit 1 | 5.54733 |
| NRG1 | Neuregulin 1 | 5.546505 |
| FASLG | Fas Ligand | 5.546445 |
| CYP2A6 | Cytochrome P450 Family 2 Subfamily A Member 6 | 5.545689 |
| TBL2 | Transducin Beta Like 2 | 5.537586 |
| PMAIP1 | Phorbol-12-Myristate-13-Acetate-Induced Protein 1 | 5.535959 |
| CYP2C9 | Cytochrome P450 Family 2 Subfamily C Member 9 | 5.535381 |
| CDKAL1 | CDK5 Regulatory Subunit Associated Protein 1 Like 1 | 5.534067 |
| HP | Haptoglobin | 5.533607 |
| PEMT | Phosphatidylethanolamine N-Methyltransferase | 5.526129 |
| VKORC1L1 | Vitamin K Epoxide Reductase Complex Subunit 1 Like 1 | 5.522895 |
| HTR3A | 5-Hydroxytryptamine Receptor 3A | 5.52095 |
| MEF2C | Myocyte Enhancer Factor 2C | 5.520911 |
| PDHB | Pyruvate Dehydrogenase E1 Subunit Beta | 5.520752 |
| ELOVL7 | ELOVL Fatty Acid Elongase 7 | 5.519201 |
| NDUFS4 | NADH:Ubiquinone Oxidoreductase Subunit S4 | 5.514392 |
| HSPA2 | Heat Shock Protein Family A (Hsp70) Member 2 | 5.514015 |
| COPB2 | COPI Coat Complex Subunit Beta 2 | 5.508985 |
| CDKN2A | Cyclin Dependent Kinase Inhibitor 2A | 5.5031 |
| ATG9A | Autophagy Related 9A | 5.501772 |
| TFEB | Transcription Factor EB | 5.501767 |
| TRAPPC12 | Trafficking Protein Particle Complex Subunit 12 | 5.495687 |
| ACSL3 | Acyl-CoA Synthetase Long Chain Family Member 3 | 5.49466 |
| CSNK2A1 | Casein Kinase 2 Alpha 1 | 5.493505 |
| CHP1 | Calcineurin Like EF-Hand Protein 1 | 5.492865 |
| TRPM8 | Transient Receptor Potential Cation Channel Subfamily M Member 8 | 5.483677 |
| UGT1A5 | UDP Glucuronosyltransferase Family 1 Member A5 | 5.477101 |
| TTF2 | Transcription Termination Factor 2 | 5.472316 |
| IAPP | Islet Amyloid Polypeptide | 5.471467 |
| PNPLA6 | Patatin Like Phospholipase Domain Containing 6 | 5.467666 |
| PURA | Purine Rich Element Binding Protein A | 5.466282 |
| FLNB | Filamin B | 5.463548 |
| CERS6 | Ceramide Synthase 6 | 5.463155 |
| ARRB1 | Arrestin Beta 1 | 5.459452 |
| NF1 | Neurofibromin 1 | 5.458903 |
| TESPA1 | Thymocyte Expressed, Positive Selection Associated 1 | 5.44838 |
| HNRNPU | Heterogeneous Nuclear Ribonucleoprotein U | 5.44686 |
| HLA-DRB1 | Major Histocompatibility Complex, Class II, DR Beta 1 | 5.445292 |
| MAN2B1 | Mannosidase Alpha Class 2B Member 1 | 5.444276 |
| KCNB1 | Potassium Voltage-Gated Channel Subfamily B Member 1 | 5.442124 |
| LIPC | Lipase C, Hepatic Type | 5.439047 |
| RUVBL2 | RuvB Like AAA ATPase 2 | 5.433352 |
| CFLAR | CASP8 And FADD Like Apoptosis Regulator | 5.431244 |
| PALS1 | Protein Associated With LIN7 1, MAGUK P55 Family Member | 5.429113 |
| SMPD2 | Sphingomyelin Phosphodiesterase 2 | 5.427896 |
| RPL10 | Ribosomal Protein L10 | 5.426992 |
| SPCS2 | Signal Peptidase Complex Subunit 2 | 5.42576 |
| SMN1 | Survival Of Motor Neuron 1, Telomeric | 5.421015 |
| NCLN | Nicalin | 5.415156 |
| SERPINI1 | Serpin Family I Member 1 | 5.413888 |
| HADHB | Hydroxyacyl-CoA Dehydrogenase Trifunctional Multienzyme Complex Subunit Beta | 5.410623 |
| CDH2 | Cadherin 2 | 5.408671 |
| NIBAN1 | Niban Apoptosis Regulator 1 | 5.406737 |
| UBE2N | Ubiquitin Conjugating Enzyme E2 N | 5.398006 |
| AGPAT2 | 1-Acylglycerol-3-Phosphate O-Acyltransferase 2 | 5.397935 |
| GFPT1 | Glutamine--Fructose-6-Phosphate Transaminase 1 | 5.39397 |
| MYRF | Myelin Regulatory Factor | 5.393103 |
| BRAF | B-Raf Proto-Oncogene, Serine/Threonine Kinase | 5.392915 |
| PRKDC | Protein Kinase, DNA-Activated, Catalytic Subunit | 5.390024 |
| ULBP1 | UL16 Binding Protein 1 | 5.388706 |
| PRKAB1 | Protein Kinase AMP-Activated Non-Catalytic Subunit Beta 1 | 5.386183 |
| LIN28A | Lin-28 Homolog A | 5.383773 |
| RIC3 | RIC3 Acetylcholine Receptor Chaperone | 5.382417 |
| DRD1 | Dopamine Receptor D1 | 5.381422 |
| DUOXA1 | Dual Oxidase Maturation Factor 1 | 5.381156 |
| ANKS4B | Ankyrin Repeat And Sterile Alpha Motif Domain Containing 4B | 5.380867 |
| HSD11B2 | Hydroxysteroid 11-Beta Dehydrogenase 2 | 5.379252 |
| OLR1 | Oxidized Low Density Lipoprotein Receptor 1 | 5.378887 |
| KCNIP4 | Potassium Voltage-Gated Channel Interacting Protein 4 | 5.375228 |
| MEF2A | Myocyte Enhancer Factor 2A | 5.370764 |
| LMNB1 | Lamin B1 | 5.370135 |
| CASR | Calcium Sensing Receptor | 5.359273 |
| SEC22C | SEC22 Homolog C, Vesicle Trafficking Protein | 5.356649 |
| SERPINA2 | Serpin Family A Member 2 (Gene/Pseudogene) | 5.352215 |
| SELP | Selectin P | 5.343926 |
| EIF2B1 | Eukaryotic Translation Initiation Factor 2B Subunit Alpha | 5.341355 |
| PEX11B | Peroxisomal Biogenesis Factor 11 Beta | 5.340438 |
| AVP | Arginine Vasopressin | 5.338967 |
| PLCG1 | Phospholipase C Gamma 1 | 5.338659 |
| PDE5A | Phosphodiesterase 5A | 5.338113 |
| ACBD3 | Acyl-CoA Binding Domain Containing 3 | 5.336448 |
| QDPR | Quinoid Dihydropteridine Reductase | 5.334661 |
| CR1 | Complement C3b/C4b Receptor 1 (Knops Blood Group) | 5.328671 |
| TIAL1 | TIA1 Cytotoxic Granule Associated RNA Binding Protein Like 1 | 5.327433 |
| ZDHHC4 | Zinc Finger DHHC-Type Palmitoyltransferase 4 | 5.325793 |
| EEF1D | Eukaryotic Translation Elongation Factor 1 Delta | 5.316687 |
| TPM1 | Tropomyosin 1 | 5.310005 |
| TMEM67 | Transmembrane Protein 67 | 5.307762 |
| CHRM3 | Cholinergic Receptor Muscarinic 3 | 5.306836 |
| LPCAT1 | Lysophosphatidylcholine Acyltransferase 1 | 5.305692 |
| ATF1 | Activating Transcription Factor 1 | 5.304689 |
| PTK2 | Protein Tyrosine Kinase 2 | 5.301264 |
| GOLGB1 | Golgin B1 | 5.297478 |
| CSTB | Cystatin B | 5.295172 |
| LDHA | Lactate Dehydrogenase A | 5.280085 |
| CASC3 | CASC3 Exon Junction Complex Subunit | 5.276394 |
| ERBB2 | Erb-B2 Receptor Tyrosine Kinase 2 | 5.261974 |
| ATG7 | Autophagy Related 7 | 5.261068 |
| CLN5 | CLN5 Intracellular Trafficking Protein | 5.257618 |
| NCSTN | Nicastrin | 5.253432 |
| SYNCRIP | Synaptotagmin Binding Cytoplasmic RNA Interacting Protein | 5.252891 |
| PLG | Plasminogen | 5.252563 |
| NUP210 | Nucleoporin 210 | 5.251059 |
| SGF29 | SAGA Complex Associated Factor 29 | 5.250876 |
| YY1 | YY1 Transcription Factor | 5.250799 |
| ELOVL1 | ELOVL Fatty Acid Elongase 1 | 5.246082 |
| MUC5AC | Mucin 5AC, Oligomeric Mucus/Gel-Forming | 5.240655 |
| NRAS | NRAS Proto-Oncogene, GTPase | 5.233254 |
| SLC9A1 | Solute Carrier Family 9 Member A1 | 5.22897 |
| OPRM1 | Opioid Receptor Mu 1 | 5.226197 |
| HNF4A | Hepatocyte Nuclear Factor 4 Alpha | 5.214128 |
| NSDHL | NAD(P) Dependent Steroid Dehydrogenase-Like | 5.209011 |
| STEEP1 | STING1 ER Exit Protein 1 | 5.205729 |
| FGF2 | Fibroblast Growth Factor 2 | 5.204324 |
| TOR1AIP1 | Torsin 1A Interacting Protein 1 | 5.204095 |
| MIR199A1 | MicroRNA 199a-1 | 5.202199 |
| AIMP1 | Aminoacyl TRNA Synthetase Complex Interacting Multifunctional Protein 1 | 5.200974 |
| PEF1 | Penta-EF-Hand Domain Containing 1 | 5.197813 |
| STARD5 | StAR Related Lipid Transfer Domain Containing 5 | 5.190176 |
| TRPC1 | Transient Receptor Potential Cation Channel Subfamily C Member 1 | 5.189981 |
| DLG1 | Discs Large MAGUK Scaffold Protein 1 | 5.18822 |
| EZH2 | Enhancer Of Zeste 2 Polycomb Repressive Complex 2 Subunit | 5.186925 |
| CDKN1B | Cyclin Dependent Kinase Inhibitor 1B | 5.175326 |
| CYP21A2 | Cytochrome P450 Family 21 Subfamily A Member 2 | 5.172648 |
| MYLK | Myosin Light Chain Kinase | 5.167694 |
| E2F1 | E2F Transcription Factor 1 | 5.166516 |
| SRL | Sarcalumenin | 5.164198 |
| KRT8 | Keratin 8 | 5.16344 |
| TLR7 | Toll Like Receptor 7 | 5.162696 |
| CTH | Cystathionine Gamma-Lyase | 5.158203 |
| BLZF1 | Basic Leucine Zipper Nuclear Factor 1 | 5.155042 |
| GH1 | Growth Hormone 1 | 5.15438 |
| POMP | Proteasome Maturation Protein | 5.150156 |
| RPLP0 | Ribosomal Protein Lateral Stalk Subunit P0 | 5.145832 |
| PTP4A1 | Protein Tyrosine Phosphatase 4A1 | 5.145557 |
| IGF1R | Insulin Like Growth Factor 1 Receptor | 5.144852 |
| STXBP1 | Syntaxin Binding Protein 1 | 5.143266 |
| OPRD1 | Opioid Receptor Delta 1 | 5.137342 |
| UBE2D1 | Ubiquitin Conjugating Enzyme E2 D1 | 5.13348 |
| TMEM199 | Transmembrane Protein 199 | 5.13261 |
| NR4A1 | Nuclear Receptor Subfamily 4 Group A Member 1 | 5.13198 |
| TJP1 | Tight Junction Protein 1 | 5.131021 |
| RPS6 | Ribosomal Protein S6 | 5.128533 |
| GABRA1 | Gamma-Aminobutyric Acid Type A Receptor Subunit Alpha1 | 5.120553 |
| EPAS1 | Endothelial PAS Domain Protein 1 | 5.120019 |
| TRAM2 | Translocation Associated Membrane Protein 2 | 5.119758 |
| NDUFS8 | NADH:Ubiquinone Oxidoreductase Core Subunit S8 | 5.113416 |
| DGAT2 | Diacylglycerol O-Acyltransferase 2 | 5.112139 |
| SPAG5 | Sperm Associated Antigen 5 | 5.109059 |
| TSC1 | TSC Complex Subunit 1 | 5.107747 |
| CCK | Cholecystokinin | 5.105546 |
| UBXN2B | UBX Domain Protein 2B | 5.10526 |
| NPPA | Natriuretic Peptide A | 5.104582 |
| SLC2A4 | Solute Carrier Family 2 Member 4 | 5.099747 |
| DEGS1 | Delta 4-Desaturase, Sphingolipid 1 | 5.092605 |
| DYNC1H1 | Dynein Cytoplasmic 1 Heavy Chain 1 | 5.090978 |
| GTF2I | General Transcription Factor IIi | 5.088943 |
| ELOVL3 | ELOVL Fatty Acid Elongase 3 | 5.087617 |
| PROS1 | Protein S | 5.084815 |
| MATN3 | Matrilin 3 | 5.07702 |
| FGFR4 | Fibroblast Growth Factor Receptor 4 | 5.074791 |
| HLA-DPB1 | Major Histocompatibility Complex, Class II, DP Beta 1 | 5.073803 |
| ENPP1 | Ectonucleotide Pyrophosphatase/Phosphodiesterase 1 | 5.069609 |
| DYRK1A | Dual Specificity Tyrosine Phosphorylation Regulated Kinase 1A | 5.069344 |
| CCL4 | C-C Motif Chemokine Ligand 4 | 5.068934 |
| BIRC2 | Baculoviral IAP Repeat Containing 2 | 5.066642 |
| DHX36 | DEAH-Box Helicase 36 | 5.066614 |
| F3 | Coagulation Factor III, Tissue Factor | 5.058293 |
| CD59 | CD59 Molecule (CD59 Blood Group) | 5.053978 |
| DELE1 | DAP3 Binding Cell Death Enhancer 1 | 5.051995 |
| MECP2 | Methyl-CpG Binding Protein 2 | 5.051294 |
| STK39 | Serine/Threonine Kinase 39 | 5.049914 |
| LRP2 | LDL Receptor Related Protein 2 | 5.048332 |
| KRTCAP2 | Keratinocyte Associated Protein 2 | 5.047668 |
| ABCC6 | ATP Binding Cassette Subfamily C Member 6 | 5.046158 |
| RAF1 | Raf-1 Proto-Oncogene, Serine/Threonine Kinase | 5.044864 |
| NAT8 | N-Acetyltransferase 8 (Putative) | 5.042976 |
| PREP | Prolyl Endopeptidase | 5.038263 |
| C6orf120 | Chromosome 6 Open Reading Frame 120 | 5.036865 |
| DDHD1 | DDHD Domain Containing 1 | 5.035842 |
| GCLC | Glutamate-Cysteine Ligase Catalytic Subunit | 5.031579 |
| C3orf52 | Chromosome 3 Open Reading Frame 52 | 5.031522 |
| CYP51A1 | Cytochrome P450 Family 51 Subfamily A Member 1 | 5.028956 |
| HACE1 | HECT Domain And Ankyrin Repeat Containing E3 Ubiquitin Protein Ligase 1 | 5.027467 |
| MIR21 | MicroRNA 21 | 5.025929 |
| VDR | Vitamin D Receptor | 5.025618 |
| PRKCQ | Protein Kinase C Theta | 5.020879 |
| LSG1 | Large 60S Subunit Nuclear Export GTPase 1 | 5.020027 |
| CCN2 | Cellular Communication Network Factor 2 | 5.018164 |
| GPI | Glucose-6-Phosphate Isomerase | 5.017212 |
| PGRMC1 | Progesterone Receptor Membrane Component 1 | 5.016309 |
| LAMP1 | Lysosomal Associated Membrane Protein 1 | 5.012759 |
| SPP1 | Secreted Phosphoprotein 1 | 5.010533 |
| FGF21 | Fibroblast Growth Factor 21 | 5.010385 |
| ATXN3 | Ataxin 3 | 5.009679 |
| RPS3 | Ribosomal Protein S3 | 5.007141 |
| MYO9A | Myosin IXA | 5.005795 |
| TGFBR1 | Transforming Growth Factor Beta Receptor 1 | 5.003658 |
| CAST | Calpastatin | 5.002892 |
| ACSF3 | Acyl-CoA Synthetase Family Member 3 | 5.002577 |
| ACER3 | Alkaline Ceramidase 3 | 5.000251 |
