# Supplementary material for: Endoplasmic Reticulum Stress-Related Four-Biomarker Risk Classifier for Survival Evaluation in Esophageal Cancer
Source: J Oncol. 2022 Mar 18;2022:5860671. doi: 10.1155/2022/5860671 (PMC8956413; doi:10.1155/2022/5860671)
Supplement: Supplementary Materials — Supplementary Table 1 The list of endoplasmic reticulum stress-related genes. [file 5860671.f1.zip › 5860671.f1/Supplementary Table 1.pdf]

Supplementary Table 1 The list of endoplasmic reticulum stress-related genes.

Gene Symbol	Description	Score
ERN1	Endoplasmic Reticulum To Nucleus Signaling 1	61.67589
HSPA5	Heat Shock Protein Family A (Hsp70) Member 5	61.20959
ATP2A2	ATPase Sarcoplasmic/Endoplasmic Reticulum Ca2+ Transporting 2	52.67031
ATP2A1	ATPase Sarcoplasmic/Endoplasmic Reticulum Ca2+ Transporting 1	50.40529
SERP1	Stress Associated Endoplasmic Reticulum Protein 1	46.38565
ATP2A3	ATPase Sarcoplasmic/Endoplasmic Reticulum Ca2+ Transporting 3	45.22342
XBP1	X-Box Binding Protein 1	44.63555
EIF2AK3	Eukaryotic Translation Initiation Factor 2 Alpha Kinase 3	43.61681
ERP29	Endoplasmic Reticulum Protein 29	43.32138
SERP2	Stress Associated Endoplasmic Reticulum Protein Family Member 2	42.57033
ERP44	Endoplasmic Reticulum Protein 44	42.19085
KDELR1	KDEL Endoplasmic Reticulum Protein Retention Receptor 1	41.6874
OS9	OS9 Endoplasmic Reticulum Lectin	40.63673
ERAP1	Endoplasmic Reticulum Aminopeptidase 1	40.45379
ERO1A	Endoplasmic Reticulum Oxidoreductase 1 Alpha	40.39962
KDELR2	KDEL Endoplasmic Reticulum Protein Retention Receptor 2	39.99511
HERPUD1	Homocysteine Inducible ER Protein With Ubiquitin Like Domain 1	39.4991
ERLEC1	Endoplasmic Reticulum Lectin 1	37.66689
VCP	Valosin Containing Protein	37.63148
ERAP2	Endoplasmic Reticulum Aminopeptidase 2	37.29198
KDELR3	KDEL Endoplasmic Reticulum Protein Retention Receptor 3	36.65843
SEC16A	SEC16 Homolog A, Endoplasmic Reticulum Export Factor	35.70557
ERN2	Endoplasmic Reticulum To Nucleus Signaling 2	35.27291
ATF6	Activating Transcription Factor 6	35.14064
ERP27	Endoplasmic Reticulum Protein 27	34.71851
RER1	Retention In Endoplasmic Reticulum Sorting Receptor 1	34.15969
ERO1B	Endoplasmic Reticulum Oxidoreductase 1 Beta	33.47313
DDIT3	DNA Damage Inducible Transcript 3	32.97501
CALR	Calreticulin	32.22462
CHERP	Calcium Homeostasis Endoplasmic Reticulum Protein	31.32324
TP53	Tumor Protein P53	30.86179
RYR2	Ryanodine Receptor 2	30.58464
HSP90B1	Heat Shock Protein 90 Beta Family Member 1	30.13174
ERMP1	Endoplasmic Reticulum Metallopeptidase 1	29.4426
CANX	Calnexin	29.07183
EIF2S1	Eukaryotic Translation Initiation Factor 2 Subunit Alpha	28.43548
ATF4	Activating Transcription Factor 4	28.40055
RYR1	Ryanodine Receptor 1	27.90683
CPT2	Carnitine Palmitoyltransferase 2	27.30067
TRDN	Triadin	26.14801
SYVN1	Synoviolin 1	25.99647

PDIA3	Protein Disulfide Isomerase Family A Member 3	25.82389
CASQ2	Calsequestrin 2	25.25162
NFE2L1	NFE2 Like BZIP Transcription Factor 1	25.09475
MAPK8	Mitogen-Activated Protein Kinase 8	24.22639
DERL2	Derlin 2	23.47571
NFE2L2	NFE2 Like BZIP Transcription Factor 2	23.25203
DERL1	Derlin 1	22.58221
LMAN1	Lectin, Mannose Binding 1	22.41786
DNAJC10	DnaJ Heat Shock Protein Family (Hsp40) Member C10	22.37406
TXNDC12	Thioredoxin Domain Containing 12	22.31909
CASQ1	Calsequestrin 1	22.09608
HMOX1	Heme Oxygenase 1	21.96422
APP	Amyloid Beta Precursor Protein	21.93855
SOD1	Superoxide Dismutase 1	21.8755
CALM1	Calmodulin 1	21.63652
P4HB	Prolyl 4-Hydroxylase Subunit Beta	21.49491
PSEN1	Presenilin 1	21.47169
HYOU1	Hypoxia Up-Regulated 1	21.25714
MAPK14	Mitogen-Activated Protein Kinase 14	21.14348
CASP3	Caspase 3	20.54427
ITPR1	Inositol 1,4,5-Trisphosphate Receptor Type 1	20.40857
SIL1	SIL1 Nucleotide Exchange Factor	20.35003
CASP4	Caspase 4	20.32967
DNAJB9	DnaJ Heat Shock Protein Family (Hsp40) Member B9	20.32507
AMFR	Autocrine Motility Factor Receptor	20.20701
PRKN	Parkin RBR E3 Ubiquitin Protein Ligase	20.15544
CAT	Catalase	20.08779
MAP3K5	Mitogen-Activated Protein Kinase Kinase Kinase 5	20.07761
MAPK1	Mitogen-Activated Protein Kinase 1	20.02571
BCAP31	B Cell Receptor Associated Protein 31	20.01274
BCL2	BCL2 Apoptosis Regulator	19.78052
SEC23A	SEC23 Homolog A, COPII Coat Complex Component	19.77968
DNAJC3	DnaJ Heat Shock Protein Family (Hsp40) Member C3	19.6843
H6PD	Hexose-6-Phosphate Dehydrogenase/Glucose 1-Dehydrogenase	19.64792
STIM1	Stromal Interaction Molecule 1	19.45386
TNF	Tumor Necrosis Factor	19.39718
PDIA4	Protein Disulfide Isomerase Family A Member 4	19.37948
CLU	Clusterin	19.292
G3BP1	G3BP Stress Granule Assembly Factor 1	19.26591
CREB3	CAMP Responsive Element Binding Protein 3	19.1912
SLC6A4	Solute Carrier Family 6 Member 4	19.05468
TXNDC5	Thioredoxin Domain Containing 5	19.00186
PPP1R15A	Protein Phosphatase 1 Regulatory Subunit 15A	18.8567

JSRP1	Junctional Sarcoplasmic Reticulum Protein 1	18.78548
DERL3	Derlin 3	18.66446
STIP1	Stress Induced Phosphoprotein 1	18.60493
BAX	BCL2 Associated X, Apoptosis Regulator	18.5895
INS	Insulin	18.30263
MAN1B1	Mannosidase Alpha Class 1B Member 1	18.2039
MANF	Mesencephalic Astrocyte Derived Neurotrophic Factor	17.943
ATF6B	Activating Transcription Factor 6 Beta	17.93627
LNPK	Lunapark, ER Junction Formation Factor	17.91348
OXSR1	Oxidative Stress Responsive Kinase 1	17.83235
RTN4	Reticulon 4	17.46556
CRH	Corticotropin Releasing Hormone	17.39136
EPM2A	EPM2A Glucan Phosphatase, Laforin	17.21646
CFTR	CF Transmembrane Conductance Regulator	17.16868
DNAJB11	DnaJ Heat Shock Protein Family (Hsp40) Member B11	17.11733
WFS1	Wolframin ER Transmembrane Glycoprotein	17.11353
STING1	Stimulator Of Interferon Response CGAMP Interactor 1	17.09591
FKBP14	FKBP Prolyl Isomerase 14	17.04955
SEL1L	SEL1L Adaptor Subunit Of ERAD E3 Ubiquitin Ligase	16.94469
CREB3L1	CAMP Responsive Element Binding Protein 3 Like 1	16.71351
HSPA1A	Heat Shock Protein Family A (Hsp70) Member 1A	16.69065
FOS	Fos Proto-Oncogene, AP-1 Transcription Factor Subunit	16.63699
PARK7	Parkinsonism Associated Deglycase	16.40114
MTOR	Mechanistic Target Of Rapamycin Kinase	16.28128
ATP13A1	ATPase 13A1	16.22049
HSPA4	Heat Shock Protein Family A (Hsp70) Member 4	16.21702
SELENOS	Selenoprotein S	16.14998
IL6	Interleukin 6	16.02559
DDRGK1	DDRGK Domain Containing 1	15.97115
MIA2	MIA SH3 Domain ER Export Factor 2	15.94477
LMNA	Lamin A/C	15.94096
KCNQ1	Potassium Voltage-Gated Channel Subfamily Q Member 1	15.88764
KCNH2	Potassium Voltage-Gated Channel Subfamily H Member 2	15.87576
HSF1	Heat Shock Transcription Factor 1	15.86728
SIRT1	Sirtuin 1	15.81215
UBC	Ubiquitin C	15.58262
ERGIC3	ERGIC And Golgi 3	15.49415
BDNF	Brain Derived Neurotrophic Factor	15.33601
SEC61A1	SEC61 Translocon Subunit Alpha 1	15.28462
SREBF1	Sterol Regulatory Element Binding Transcription Factor 1	15.26151
SIGMAR1	Sigma Non-Opioid Intracellular Receptor 1	15.21339
G3BP2	G3BP Stress Granule Assembly Factor 2	15.11395
RPN1	Ribophorin I	14.9468

SELENON	Selenoprotein N	14.94155
HSPA8	Heat Shock Protein Family A (Hsp70) Member 8	14.92215
PDIA2	Protein Disulfide Isomerase Family A Member 2	14.88027
IL1B	Interleukin 1 Beta	14.84518
PTPN1	Protein Tyrosine Phosphatase Non-Receptor Type 1	14.81349
CYCS	Cytochrome C, Somatic	14.75115
HSP90AA1	Heat Shock Protein 90 Alpha Family Class A Member 1	14.74063
ERLIN2	ER Lipid Raft Associated 2	14.64168
BAG6	BAG Cochaperone 6	14.62383
SURF4	Surfeit 4	14.54184
VAPB	VAMP Associated Protein B And C	14.52928
CXCL8	C-X-C Motif Chemokine Ligand 8	14.45609
INSIG1	Insulin Induced Gene 1	14.43716
GSR	Glutathione-Disulfide Reductase	14.3992
NHLRC1	NHL Repeat Containing E3 Ubiquitin Protein Ligase 1	14.35578
NOS3	Nitric Oxide Synthase 3	14.32792
NR3C1	Nuclear Receptor Subfamily 3 Group C Member 1	14.2975
PKP2	Plakophilin 2	14.28161
SREBF2	Sterol Regulatory Element Binding Transcription Factor 2	14.24426
HSPB1	Heat Shock Protein Family B (Small) Member 1	14.16895
CREB3L2	CAMP Responsive Element Binding Protein 3 Like 2	14.16421
SELENOK	Selenoprotein K	14.08746
APOE	Apolipoprotein E	14.05471
ANK2	Ankyrin 2	13.99543
DDX3X	DEAD-Box Helicase 3 X-Linked	13.88916
JUN	Jun Proto-Oncogene, AP-1 Transcription Factor Subunit	13.79473
SEC31A	SEC31 Homolog A, COPII Coat Complex Component	13.76338
TMED4	Transmembrane P24 Trafficking Protein 4	13.7511
SCAP	SREBF Chaperone	13.74349
SEC24A	SEC24 Homolog A, COPII Coat Complex Component	13.70224
SOD2	Superoxide Dismutase 2	13.69118
MIA3	MIA SH3 Domain ER Export Factor 3	13.67494
HMGCR	3-Hydroxy-3-Methylglutaryl-CoA Reductase	13.62906
TGFB1	Transforming Growth Factor Beta 1	13.60651
SNCA	Synuclein Alpha	13.58748
ADIPOQ	Adiponectin, C1Q And Collagen Domain Containing	13.56961
RTN3	Reticulon 3	13.54299
TXN	Thioredoxin	13.51265
SCN5A	Sodium Voltage-Gated Channel Alpha Subunit 5	13.50983
EDEM1	ER Degradation Enhancing Alpha-Mannosidase Like Protein 1	13.4877
EIF2AK2	Eukaryotic Translation Initiation Factor 2 Alpha Kinase 2	13.46035
ASPH	Aspartate Beta-Hydroxylase	13.40934
NOTCH3	Notch Receptor 3	13.36577

RPS27A	Ribosomal Protein S27a	13.34644
LMAN2	Lectin, Mannose Binding 2	13.3444
BSCL2	BSCL2 Lipid Droplet Biogenesis Associated, Seipin	13.32844
CREB3L3	CAMP Responsive Element Binding Protein 3 Like 3	13.31832
SEC13	SEC13 Homolog, Nuclear Pore And COPII Coat Complex Component	13.31024
RAB1A	RAB1A, Member RAS Oncogene Family	13.28846
CKAP4	Cytoskeleton Associated Protein 4	13.27768
PSEN2	Presenilin 2	13.26591
PARP1	Poly(ADP-Ribose) Polymerase 1	13.21008
TRAM1	Translocation Associated Membrane Protein 1	13.20531
PTGS2	Prostaglandin-Endoperoxide Synthase 2	13.19218
ERLIN1	ER Lipid Raft Associated 1	13.17359
TAPBP	TAP Binding Protein	13.16527
MAPK10	Mitogen-Activated Protein Kinase 10	13.13984
RRBP1	Ribosome Binding Protein 1	13.13486
SQSTM1	Sequestosome 1	13.11823
RAB1B	RAB1B, Member RAS Oncogene Family	13.11014
ERGIC2	ERGIC And Golgi 2	13.09624
SEC24B	SEC24 Homolog B, COPII Coat Complex Component	13.0681
CRHR1	Corticotropin Releasing Hormone Receptor 1	13.04762
ESR1	Estrogen Receptor 1	13.0175
CASP9	Caspase 9	13.01397
CLN3	CLN3 Lysosomal/Endosomal Transmembrane Protein, Battenin	12.9609
CALM3	Calmodulin 3	12.85714
DMD	Dystrophin	12.83013
VWF	Von Willebrand Factor	12.78849
ARL6IP1	ADP Ribosylation Factor Like GTPase 6 Interacting Protein 1	12.77665
TOR1A	Torsin Family 1 Member A	12.75956
SERPINA1	Serpin Family A Member 1	12.75667
CASP8	Caspase 8	12.75503
UFL1	UFM1 Specific Ligase 1	12.74301
STIM2	Stromal Interaction Molecule 2	12.72384
YIPF5	Yip1 Domain Family Member 5	12.70669
BNIP1	BCL2 Interacting Protein 1	12.69318
P4HTM	Prolyl 4-Hydroxylase, Transmembrane	12.66548
ALG1	ALG1 Chitobiosyldiphosphodolichol Beta-Mannosyltransferase	12.63918
CRP	C-Reactive Protein	12.61874
ATF3	Activating Transcription Factor 3	12.6033
CAV3	Caveolin 3	12.5933
ATL3	Atlastin GTPase 3	12.5618
MPO	Myeloperoxidase	12.55774
SESN2	Sestrin 2	12.45641
PPIB	Peptidylprolyl Isomerase B	12.44719

CISD2	CDGSH Iron Sulfur Domain 2	12.42839
LRRK2	Leucine Rich Repeat Kinase 2	12.40189
TAP1	Transporter 1, ATP Binding Cassette Subfamily B Member	12.39668
KEAP1	Kelch Like ECH Associated Protein 1	12.34749
POMC	Proopiomelanocortin	12.33138
TMED2	Transmembrane P24 Trafficking Protein 2	12.32648
VEGFA	Vascular Endothelial Growth Factor A	12.25063
AKT1	AKT Serine/Threonine Kinase 1	12.23633
INSIG2	Insulin Induced Gene 2	12.23219
CD4	CD4 Molecule	12.1997
PLN	Phospholamban	12.19842
UGGT1	UDP-Glucose Glycoprotein Glucosyltransferase 1	12.19679
HSPA9	Heat Shock Protein Family A (Hsp70) Member 9	12.14654
JPH2	Junctophilin 2	12.14545
NOX4	NADPH Oxidase 4	12.12688
EIF2AK1	Eukaryotic Translation Initiation Factor 2 Alpha Kinase 1	12.11481
ABL1	ABL Proto-Oncogene 1, Non-Receptor Tyrosine Kinase	12.06154
RSAD2	Radical S-Adenosyl Methionine Domain Containing 2	12.02475
APOB	Apolipoprotein B	12.01772
SAR1B	Secretion Associated Ras Related GTPase 1B	11.98537
COPB1	COPI Coat Complex Subunit Beta 1	11.95759
SLN	Sarcolipin	11.9542
CCL2	C-C Motif Chemokine Ligand 2	11.9479
HERPUD2	HERPUD Family Member 2	11.89472
GJA1	Gap Junction Protein Alpha 1	11.89176
SPAST	Spastin	11.86323
SGK1	Serum/Glucocorticoid Regulated Kinase 1	11.80528
STX17	Syntaxin 17	11.78632
SEC62	SEC62 Homolog, Preprotein Translocation Factor	11.75638
CALM2	Calmodulin 2	11.75332
GET3	Guided Entry Of Tail-Anchored Proteins Factor 3, ATPase	11.71967
TRIM13	Tripartite Motif Containing 13	11.71597
SEC24C	SEC24 Homolog C, COPII Coat Complex Component	11.7069
HLA-B	Major Histocompatibility Complex, Class I, B	11.69621
CACNA1C	Calcium Voltage-Gated Channel Subunit Alpha1 C	11.69508
ORAI1	ORAI Calcium Release-Activated Calcium Modulator 1	11.67704
DDOST	Dolichyl-Diphosphooligosaccharide--Protein Glycosyltransferase Non-Catalytic Subunit	11.66921
TMBIM6	Transmembrane BAX Inhibitor Motif Containing 6	11.62822
PRNP	Prion Protein	11.6281
PDIA6	Protein Disulfide Isomerase Family A Member 6	11.62073
EIF4G1	Eukaryotic Translation Initiation Factor 4 Gamma 1	11.60152
BCL2L1	BCL2 Like 1	11.58755
PRKCD	Protein Kinase C Delta	11.57875

EGFR	Epidermal Growth Factor Receptor	11.53666
GBF1	Golgi Brefeldin A Resistant Guanine Nucleotide Exchange Factor 1	11.52339
SLC37A4	Solute Carrier Family 37 Member 4	11.52257
UBE2J1	Ubiquitin Conjugating Enzyme E2 J1	11.51135
PDCD6	Programmed Cell Death 6	11.48717
SEC63	SEC63 Homolog, Protein Translocation Regulator	11.48514
SEC61B	SEC61 Translocon Subunit Beta	11.47974
EDEM2	ER Degradation Enhancing Alpha-Mannosidase Like Protein 2	11.47885
ALB	Albumin	11.47502
ESYT1	Extended Synaptotagmin 1	11.46611
VIM	Vimentin	11.45302
CAPN3	Calpain 3	11.43749
TARDBP	TAR DNA Binding Protein	11.42282
DNAH8	Dynein Axonemal Heavy Chain 8	11.4035
ATL1	Atlastin GTPase 1	11.37472
MYOC	Myocilin	11.37471
MAPK9	Mitogen-Activated Protein Kinase 9	11.35633
XDH	Xanthine Dehydrogenase	11.35188
CANT1	Calcium Activated Nucleotidase 1	11.34012
RNF139	Ring Finger Protein 139	11.33491
SERPINH1	Serpin Family H Member 1	11.33261
HTRA2	HtrA Serine Peptidase 2	11.31307
PREB	Prolactin Regulatory Element Binding	11.30108
OSBPL8	Oxysterol Binding Protein Like 8	11.21776
CAV1	Caveolin 1	11.18445
TAP2	Transporter 2, ATP Binding Cassette Subfamily B Member	11.15477
TMED10	Transmembrane P24 Trafficking Protein 10	11.15173
BAK1	BCL2 Antagonist/Killer 1	11.10286
PRDX4	Peroxiredoxin 4	11.0893
TMEM33	Transmembrane Protein 33	11.05001
TFG	Trafficking From ER To Golgi Regulator	11.04788
SOAT1	Sterol O-Acyltransferase 1	11.04239
SAR1A	Secretion Associated Ras Related GTPase 1A	11.0314
HSD17B10	Hydroxysteroid 17-Beta Dehydrogenase 10	11.02224
STUB1	STIP1 Homology And U-Box Containing Protein 1	11.01499
EDEM3	ER Degradation Enhancing Alpha-Mannosidase Like Protein 3	11.00724
FOXO1	Forkhead Box O1	11.00432
PINK1	PTEN Induced Kinase 1	10.99677
RYR3	Ryanodine Receptor 3	10.98407
RTN1	Reticulon 1	10.9587
VHL	Von Hippel-Lindau Tumor Suppressor	10.93574
PRKAA1	Protein Kinase AMP-Activated Catalytic Subunit Alpha 1	10.92647
TEX264	Testis Expressed 264, ER-Phagy Receptor	10.91878

VAPA	VAMP Associated Protein A	10.91279
SEC24D	SEC24 Homolog D, COPII Coat Complex Component	10.91228
RPN2	Ribophorin II	10.91054
CYBA	Cytochrome B-245 Alpha Chain	10.87929
TRIP11	Thyroid Hormone Receptor Interactor 11	10.87178
BACE1	Beta-Secretase 1	10.84985
TOR1B	Torsin Family 1 Member B	10.84638
FOXO3	Forkhead Box O3	10.83612
TLR4	Toll Like Receptor 4	10.81847
AUP1	AUP1 Lipid Droplet Regulating VLDL Assembly Factor	10.77417
RNF185	Ring Finger Protein 185	10.76441
TECRL	Trans-2,3-Enoyl-CoA Reductase Like	10.74385
TMED9	Transmembrane P24 Trafficking Protein 9	10.73923
GSK3B	Glycogen Synthase Kinase 3 Beta	10.73245
HSPA1B	Heat Shock Protein Family A (Hsp70) Member 1B	10.72249
NFKB1	Nuclear Factor Kappa B Subunit 1	10.71609
UBQLN1	Ubiquilin 1	10.71093
RCN2	Reticulocalbin 2	10.703
TRAF2	TNF Receptor Associated Factor 2	10.67353
MOGS	Mannosyl-Oligosaccharide Glucosidase	10.66786
ATM	ATM Serine/Threonine Kinase	10.64906
DHCR24	24-Dehydrocholesterol Reductase	10.64183
PCSK9	Proprotein Convertase Subtilisin/Kexin Type 9	10.63497
NOS2	Nitric Oxide Synthase 2	10.62834
STX18	Syntaxin 18	10.60463
FMR1	FMRP Translational Regulator 1	10.59731
PMM2	Phosphomannomutase 2	10.56867
UGGT2	UDP-Glucose Glycoprotein Glucosyltransferase 2	10.56759
STX5	Syntaxin 5	10.50949
FAF2	Fas Associated Factor Family Member 2	10.50076
AHCYL1	Adenosylhomocysteinase Like 1	10.48951
FKBP5	FKBP Prolyl Isomerase 5	10.48365
TMEM208	Transmembrane Protein 208	10.48354
KCNE1	Potassium Voltage-Gated Channel Subfamily E Regulatory Subunit 1	10.4642
G6PC1	Glucose-6-Phosphatase Catalytic Subunit 1	10.45968
CEBPB	CCAAT Enhancer Binding Protein Beta	10.45587
MAPK3	Mitogen-Activated Protein Kinase 3	10.45374
UBA52	Ubiquitin A-52 Residue Ribosomal Protein Fusion Product 1	10.44676
TNFRSF10B	TNF Receptor Superfamily Member 10b	10.44447
CCDC47	Coiled-Coil Domain Containing 47	10.43917
SLC8A1	Solute Carrier Family 8 Member A1	10.43395
TXNIP	Thioredoxin Interacting Protein	10.42945
IER3IP1	Immediate Early Response 3 Interacting Protein 1	10.38209

CCDC88B	Coiled-Coil Domain Containing 88B	10.37848
KCNJ5	Potassium Inwardly Rectifying Channel Subfamily J Member 5	10.36464
QRICH1	Glutamine Rich 1	10.36217
EGF	Epidermal Growth Factor	10.35225
CYP2E1	Cytochrome P450 Family 2 Subfamily E Member 1	10.32307
COMP	Cartilage Oligomeric Matrix Protein	10.30769
FOXRED2	FAD Dependent Oxidoreductase Domain Containing 2	10.30243
PIEZO1	Piezo Type Mechanosensitive Ion Channel Component 1	10.30134
COMT	Catechol-O-Methyltransferase	10.28566
BBC3	BCL2 Binding Component 3	10.28216
MAPT	Microtubule Associated Protein Tau	10.27945
NQO1	NAD(P)H Quinone Dehydrogenase 1	10.25786
RAB10	RAB10, Member RAS Oncogene Family	10.2107
G6PD	Glucose-6-Phosphate Dehydrogenase	10.20141
RAB6A	RAB6A, Member RAS Oncogene Family	10.19674
NLRP3	NLR Family Pyrin Domain Containing 3	10.19188
PON1	Paraoxonase 1	10.17631
COL7A1	Collagen Type VII Alpha 1 Chain	10.16891
HLA-A	Major Histocompatibility Complex, Class I, A	10.15979
STARD3	StAR Related Lipid Transfer Domain Containing 3	10.15604
BECN1	Beclin 1	10.14536
CYP1A2	Cytochrome P450 Family 1 Subfamily A Member 2	10.14344
LOC110806262	Solute Carrier Family 6 Member 4 Gene Promoter	10.14253
EMC1	ER Membrane Protein Complex Subunit 1	10.13597
MAPKAPK2	MAPK Activated Protein Kinase 2	10.11254
COPA	COPI Coat Complex Subunit Alpha	10.10142
TG	Thyroglobulin	10.09978
CDKN1A	Cyclin Dependent Kinase Inhibitor 1A	10.08722
TOR1AIP2	Torsin 1A Interacting Protein 2	10.08272
SEC23IP	SEC23 Interacting Protein	10.07786
ESYT2	Extended Synaptotagmin 2	10.06518
AQP11	Aquaporin 11	10.05941
SRPRA	SRP Receptor Subunit Alpha	10.01753
DSP	Desmoplakin	10.01209
EIF2AK4	Eukaryotic Translation Initiation Factor 2 Alpha Kinase 4	9.963552
KTN1	Kinectin 1	9.935626
FKRP	Fukutin Related Protein	9.918791
POGLUT2	Protein O-Glucosyltransferase 2	9.914181
GPR37	G Protein-Coupled Receptor 37	9.908205
PRKAA2	Protein Kinase AMP-Activated Catalytic Subunit Alpha 2	9.907925
TMX3	Thioredoxin Related Transmembrane Protein 3	9.863596
GAPDH	Glyceraldehyde-3-Phosphate Dehydrogenase	9.855058

CYBB	Cytochrome B-245 Beta Chain	9.835807
THBS1	Thrombospondin 1	9.831228
DNM1L	Dynamin 1 Like	9.827184
VKORC1	Vitamin K Epoxide Reductase Complex Subunit 1	9.8061
F2	Coagulation Factor II, Thrombin	9.805059
SLC2A1	Solute Carrier Family 2 Member 1	9.747637
UBB	Ubiquitin B	9.731034
HSPD1	Heat Shock Protein Family D (Hsp60) Member 1	9.720814
TRPV4	Transient Receptor Potential Cation Channel Subfamily V Member 4	9.714215
CYB5R3	Cytochrome B5 Reductase 3	9.707078
APEX1	Apurinic/Apyrimidinic Endodeoxyribonuclease 1	9.679354
BCL2L11	BCL2 Like 11	9.677199
MZB1	Marginal Zone B And B1 Cell Specific Protein	9.673955
SEC22B	SEC22 Homolog B, Vesicle Trafficking Protein	9.670108
SHH	Sonic Hedgehog Signaling Molecule	9.624571
MAP2K6	Mitogen-Activated Protein Kinase Kinase 6	9.623247
CRYAB	Crystallin Alpha B	9.608627
ATL2	Atlastin GTPase 2	9.607834
CYP1A1	Cytochrome P450 Family 1 Subfamily A Member 1	9.566708
SEC23B	SEC23 Homolog B, COPII Coat Complex Component	9.552957
KCNE2	Potassium Voltage-Gated Channel Subfamily E Regulatory Subunit 2	9.540857
ZDHHC6	Zinc Finger DHHC-Type Palmitoyltransferase 6	9.530926
DHCR7	7-Dehydrocholesterol Reductase	9.513714
USO1	USO1 Vesicle Transport Factor	9.487221
TMX2	Thioredoxin Related Transmembrane Protein 2	9.459623
DMPK	DM1 Protein Kinase	9.441179
SMPD1	Sphingomyelin Phosphodiesterase 1	9.419294
SRP68	Signal Recognition Particle 68	9.409531
HTT	Huntingtin	9.403862
SRC	SRC Proto-Oncogene, Non-Receptor Tyrosine Kinase	9.367157
GPX1	Glutathione Peroxidase 1	9.351853
UBE2G2	Ubiquitin Conjugating Enzyme E2 G2	9.347162
ITPR3	Inositol 1,4,5-Trisphosphate Receptor Type 3	9.343953
HIF1A	Hypoxia Inducible Factor 1 Subunit Alpha	9.341242
MAP1LC3A	Microtubule Associated Protein 1 Light Chain 3 Alpha	9.331038
CLN6	CLN6 Transmembrane ER Protein	9.330304
GABARAPL1	GABA Type A Receptor Associated Protein Like 1	9.317569
OXT	Oxytocin/Neurophysin I Prepropeptide	9.307431
OSBP	Oxysterol Binding Protein	9.29965
MAPK13	Mitogen-Activated Protein Kinase 13	9.298482
GPER1	G Protein-Coupled Estrogen Receptor 1	9.297143
NOTCH1	Notch Receptor 1	9.289087
P3H4	Prolyl 3-Hydroxylase Family Member 4 (Inactive)	9.27723

MDM2	MDM2 Proto-Oncogene	9.253346
NPC1	NPC Intracellular Cholesterol Transporter 1	9.251797
GBA	Glucosylceramidase Beta	9.251139
DPAGT1	Dolichyl-Phosphate N-Acetylglucosaminephosphotransferase 1	9.248029
MAP2K7	Mitogen-Activated Protein Kinase Kinase 7	9.23126
UGT1A1	UDP Glucuronosyltransferase Family 1 Member A1	9.218047
NUPR1	Nuclear Protein 1, Transcriptional Regulator	9.215899
SCD	Stearoyl-CoA Desaturase	9.210697
PKD2	Polycystin 2, Transient Receptor Potential Cation Channel	9.20929
SCAPER	S-Phase Cyclin A Associated Protein In The ER	9.193541
ICMT	Isoprenylcysteine Carboxyl Methyltransferase	9.189874
HRC	Histidine Rich Calcium Binding Protein	9.180876
LDLR	Low Density Lipoprotein Receptor	9.178143
F9	Coagulation Factor IX	9.156574
BET1	Bet1 Golgi Vesicular Membrane Trafficking Protein	9.148979
TMX1	Thioredoxin Related Transmembrane Protein 1	9.141653
CACNA1S	Calcium Voltage-Gated Channel Subunit Alpha1 S	9.131956
MBTPS1	Membrane Bound Transcription Factor Peptidase, Site 1	9.126532
AGR2	Anterior Gradient 2, Protein Disulphide Isomerase Family Member	9.119012
CNIH4	Cornichon Family AMPA Receptor Auxiliary Protein 4	9.111598
S100A1	S100 Calcium Binding Protein A1	9.078587
JPH4	Junctophilin 4	9.068914
CASP7	Caspase 7	9.048899
PPARG	Peroxisome Proliferator Activated Receptor Gamma	9.030825
PCSK6	Proprotein Convertase Subtilisin/Kexin Type 6	9.01847
BOK	BCL2 Family Apoptosis Regulator BOK	8.994361
SMPD4	Sphingomyelin Phosphodiesterase 4	8.985379
DRD2	Dopamine Receptor D2	8.970654
UBXN8	UBX Domain Protein 8	8.957863
UGT1A6	UDP Glucuronosyltransferase Family 1 Member A6	8.94973
TMEM214	Transmembrane Protein 214	8.943626
ALG13	ALG13 UDP-N-Acetylglucosaminyltransferase Subunit	8.943331
SEC61G	SEC61 Translocon Subunit Gamma	8.941509
RHOA	Ras Homolog Family Member A	8.939493
RNFT1	Ring Finger Protein, Transmembrane 1	8.930634
PITPNB	Phosphatidylinositol Transfer Protein Beta	8.930323
PRDX2	Peroxiredoxin 2	8.927347
STT3A	STT3 Oligosaccharyltransferase Complex Catalytic Subunit A	8.923197
ARL6IP5	ADP Ribosylation Factor Like GTPase 6 Interacting Protein 5	8.922329
EMC10	ER Membrane Protein Complex Subunit 10	8.897445
CREB1	CAMP Responsive Element Binding Protein 1	8.886888
AKAP9	A-Kinase Anchoring Protein 9	8.878514
PLEKHF2	Pleckstrin Homology And FYVE Domain Containing 2	8.873873

VCAM1	Vascular Cell Adhesion Molecule 1	8.872223
PRDX1	Peroxiredoxin 1	8.87097
VMP1	Vacuole Membrane Protein 1	8.844719
MAOA	Monoamine Oxidase A	8.833247
PIGN	Phosphatidylinositol Glycan Anchor Biosynthesis Class N	8.829769
MTTP	Microsomal Triglyceride Transfer Protein	8.82287
MAP1LC3B	Microtubule Associated Protein 1 Light Chain 3 Beta	8.819851
EPO	Erythropoietin	8.80982
BRSK2	BR Serine/Threonine Kinase 2	8.806129
JAK2	Janus Kinase 2	8.800504
STAU1	Staufen Double-Stranded RNA Binding Protein 1	8.794632
MAP2K4	Mitogen-Activated Protein Kinase Kinase 4	8.788831
GABARAPL2	GABA Type A Receptor Associated Protein Like 2	8.78162
INPP5K	Inositol Polyphosphate-5-Phosphatase K	8.780443
PIK3R1	Phosphoinositide-3-Kinase Regulatory Subunit 1	8.777287
HM13	Histocompatibility Minor 13	8.767171
TMCC1	Transmembrane And Coiled-Coil Domain Family 1	8.762245
ZW10	Zw10 Kinetochore Protein	8.760718
DNAJB12	DnaJ Heat Shock Protein Family (Hsp40) Member B12	8.751414
CASP12	Caspase 12 (Gene/Pseudogene)	8.749605
RINT1	RAD50 Interactor 1	8.733054
RNF186	Ring Finger Protein 186	8.723569
TRIB3	Tribbles Pseudokinase 3	8.722757
SDHA	Succinate Dehydrogenase Complex Flavoprotein Subunit A	8.706469
FICD	FIC Domain Protein Adenylyltransferase	8.703594
UBE2J2	Ubiquitin Conjugating Enzyme E2 J2	8.699154
RAB2A	RAB2A, Member RAS Oncogene Family	8.690569
LONP1	Lon Peptidase 1, Mitochondrial	8.689078
BRCA1	BRCA1 DNA Repair Associated	8.68313
SNTA1	Syntrophin Alpha 1	8.681916
HFE	Homeostatic Iron Regulator	8.678569
MUC1	Mucin 1, Cell Surface Associated	8.676291
HSP90AB1	Heat Shock Protein 90 Alpha Family Class B Member 1	8.67391
IL10	Interleukin 10	8.664721
PON2	Paraoxonase 2	8.658606
SDF2L1	Stromal Cell Derived Factor 2 Like 1	8.648069
AIFM1	Apoptosis Inducing Factor Mitochondria Associated 1	8.64011
CAMK2G	Calcium/Calmodulin Dependent Protein Kinase II Gamma	8.624817
TMED7	Transmembrane P24 Trafficking Protein 7	8.616282
EP300	E1A Binding Protein P300	8.611076
PRDX6	Peroxiredoxin 6	8.604853
OSBPL3	Oxysterol Binding Protein Like 3	8.602255
TUSC3	Tumor Suppressor Candidate 3	8.567719

EDN1	Endothelin 1	8.564335
JPH1	Junctophilin 1	8.550062
MICB	MHC Class I Polypeptide-Related Sequence B	8.547023
ORMDL3	ORMDL Sphingolipid Biosynthesis Regulator 3	8.533163
C9orf72	C9orf72-SMCR8 Complex Subunit	8.510822
TMED1	Transmembrane P24 Trafficking Protein 1	8.500257
ELN	Elastin	8.493933
GOLGA2	Golgin A2	8.493762
TMTC3	Transmembrane O-Mannosyltransferase Targeting Cadherins 3	8.491985
SHISA5	Shisa Family Member 5	8.49105
TF	Transferrin	8.484453
EBP	EBP Cholestenol Delta-Isomerase	8.479836
SLC39A14	Solute Carrier Family 39 Member 14	8.470048
PTEN	Phosphatase And Tensin Homolog	8.465764
CTNNB1	Catenin Beta 1	8.455572
GSTM1	Glutathione S-Transferase Mu 1	8.452892
UBA5	Ubiquitin Like Modifier Activating Enzyme 5	8.44866
HMGB1	High Mobility Group Box 1	8.446188
IFNG	Interferon Gamma	8.440768
NR3C2	Nuclear Receptor Subfamily 3 Group C Member 2	8.4305
SCP2	Sterol Carrier Protein 2	8.420654
UBXN4	UBX Domain Protein 4	8.413774
CERT1	Ceramide Transporter 1	8.403917
ATG14	Autophagy Related 14	8.399909
F8	Coagulation Factor VIII	8.386883
GRIA1	Glutamate Ionotropic Receptor AMPA Type Subunit 1	8.384485
GABARAP	GABA Type A Receptor-Associated Protein	8.360096
RCN1	Reticulocalbin 1	8.357349
HSD11B1	Hydroxysteroid 11-Beta Dehydrogenase 1	8.357158
CARD14	Caspase Recruitment Domain Family Member 14	8.353963
AKAP6	A-Kinase Anchoring Protein 6	8.350359
RFT1	RFT1 Homolog	8.350307
PIGK	Phosphatidylinositol Glycan Anchor Biosynthesis Class K	8.338228
GH-LCR	Growth Hormone Locus Control Region	8.331729
TSPO	Translocator Protein	8.305192
P3H1	Prolyl 3-Hydroxylase 1	8.296486
POMT2	Protein O-Mannosyltransferase 2	8.291186
ATR	ATR Serine/Threonine Kinase	8.289417
RAC1	Rac Family Small GTPase 1	8.288699
AGR3	Anterior Gradient 3, Protein Disulphide Isomerase Family Member	8.285676
ALG14	ALG14 UDP-N-Acetylglucosaminyltransferase Subunit	8.283592
ADRB2	Adrenoceptor Beta 2	8.282927
NCK1	NCK Adaptor Protein 1	8.282869

PTGS1	Prostaglandin-Endoperoxide Synthase 1	8.272216
MPPE1	Metallophosphoesterase 1	8.265921
SP1	Sp1 Transcription Factor	8.245627
TMCO1	Transmembrane And Coiled-Coil Domains 1	8.245381
YKT6	YKT6 V-SNARE Homolog	8.244467
PPP1R15B	Protein Phosphatase 1 Regulatory Subunit 15B	8.243247
EEF1A1	Eukaryotic Translation Elongation Factor 1 Alpha 1	8.239214
LBR	Lamin B Receptor	8.211822
ZC3H12A	Zinc Finger CCCH-Type Containing 12A	8.210092
NAGLU	N-Acetyl-Alpha-Glucosaminidase	8.206962
CASP2	Caspase 2	8.205569
MBTPS2	Membrane Bound Transcription Factor Peptidase, Site 2	8.200813
CYP2D6	Cytochrome P450 Family 2 Subfamily D Member 6	8.199604
ITPR2	Inositol 1,4,5-Trisphosphate Receptor Type 2	8.197124
CDK1	Cyclin Dependent Kinase 1	8.189769
CNR1	Cannabinoid Receptor 1	8.18391
FKBP1B	FKBP Prolyl Isomerase 1B	8.169371
ATP1A3	ATPase Na+/K+ Transporting Subunit Alpha 3	8.161877
INSR	Insulin Receptor	8.155162
GRIN1	Glutamate Ionotropic Receptor NMDA Type Subunit 1	8.15115
HACD2	3-Hydroxyacyl-CoA Dehydratase 2	8.151022
GET4	Guided Entry Of Tail-Anchored Proteins Factor 4	8.13945
MCFD2	Multiple Coagulation Factor Deficiency 2, ER Cargo Receptor Complex Subunit	8.134626
IGF1	Insulin Like Growth Factor 1	8.126129
GRAMD1A	GRAM Domain Containing 1A	8.122696
CALHM1	Calcium Homeostasis Modulator 1	8.110737
DBH	Dopamine Beta-Hydroxylase	8.099258
PIK3C3	Phosphatidylinositol 3-Kinase Catalytic Subunit Type 3	8.081832
SCFD1	Sec1 Family Domain Containing 1	8.074809
SACM1L	SAC1 Like Phosphatidylinositide Phosphatase	8.068534
GLA	Galactosidase Alpha	8.067577
ALG2	ALG2 Alpha-1,3/1,6-Mannosyltransferase	8.064196
TLR9	Toll Like Receptor 9	8.062104
UBL4A	Ubiquitin Like 4A	8.058124
GET1	Guided Entry Of Tail-Anchored Proteins Factor 1	8.053599
MYH7	Myosin Heavy Chain 7	8.03334
MAPKAP1	MAPK Associated Protein 1	8.031942
PDZD8	PDZ Domain Containing 8	8.027519
CREBRF	CREB3 Regulatory Factor	8.025234
SHC1	SHC Adaptor Protein 1	8.016661
ERMARD	ER Membrane Associated RNA Degradation	8.010435
HMGCLL1	3-Hydroxymethyl-3-Methylglutaryl-CoA Lyase Like 1	7.999342
UBE2K	Ubiquitin Conjugating Enzyme E2 K	7.980807

KDR	Kinase Insert Domain Receptor	7.97788
SELENOF	Selenoprotein F	7.969396
HSPA13	Heat Shock Protein Family A (Hsp70) Member 13	7.966587
CLN8	CLN8 Transmembrane ER And ERGIC Protein	7.963565
LPCAT3	Lysophosphatidylcholine Acyltransferase 3	7.960769
KCNJ11	Potassium Inwardly Rectifying Channel Subfamily J Member 11	7.949436
JKAMP	JNK1/MAPK8 Associated Membrane Protein	7.944636
DLD	Dihydrolipoamide Dehydrogenase	7.942204
ZFAND2B	Zinc Finger AN1-Type Containing 2B	7.921453
BCL2L10	BCL2 Like 10	7.919024
JPH3	Junctophilin 3	7.91677
EEF2	Eukaryotic Translation Elongation Factor 2	7.914554
SEC11A	SEC11 Homolog A, Signal Peptidase Complex Subunit	7.909825
GPX7	Glutathione Peroxidase 7	7.902972
EMC7	ER Membrane Protein Complex Subunit 7	7.90077
HGSNAT	Heparan-Alpha-Glucosaminide N-Acetyltransferase	7.898723
PABPC1	Poly(A) Binding Protein Cytoplasmic 1	7.895919
KDSR	3-Ketodihydrosphingosine Reductase	7.895583
SGTA	Small Glutamine Rich Tetratricopeptide Repeat Co-Chaperone Alpha	7.891774
SCAMP5	Secretory Carrier Membrane Protein 5	7.890406
GANAB	Glucosidase II Alpha Subunit	7.889201
G6PC3	Glucose-6-Phosphatase Catalytic Subunit 3	7.889106
NDRG1	N-Myc Downstream Regulated 1	7.885159
MOSPD2	Motile Sperm Domain Containing 2	7.873013
UFM1	Ubiquitin Fold Modifier 1	7.848809
NR1H2	Nuclear Receptor Subfamily 1 Group H Member 2	7.847532
SOAT2	Sterol O-Acyltransferase 2	7.843211
MAP2K1	Mitogen-Activated Protein Kinase Kinase 1	7.841968
GSTP1	Glutathione S-Transferase Pi 1	7.840194
PRKCA	Protein Kinase C Alpha	7.837716
UNC93B1	Unc-93 Homolog B1, TLR Signaling Regulator	7.836614
YIF1A	Yip1 Interacting Factor Homolog A, Membrane Trafficking Protein	7.835884
PTPN2	Protein Tyrosine Phosphatase Non-Receptor Type 2	7.827527
SGPP1	Sphingosine-1-Phosphate Phosphatase 1	7.814847
MSRB3	Methionine Sulfoxide Reductase B3	7.804884
CDKN3	Cyclin Dependent Kinase Inhibitor 3	7.804855
KIF1C	Kinesin Family Member 1C	7.798558
UMOD	Uromodulin	7.793993
MX1	MX Dynamin Like GTPase 1	7.793513
LACC1	Laccase Domain Containing 1	7.786711
NSF	N-Ethylmaleimide Sensitive Factor, Vesicle Fusing ATPase	7.786564
MFN2	Mitofusin 2	7.785871
CHAT	Choline O-Acetyltransferase	7.782014

ACTB	Actin Beta	7.774903
GORASP1	Golgi Reassembly Stacking Protein 1	7.759888
SCARA3	Scavenger Receptor Class A Member 3	7.751351
TFRC	Transferrin Receptor	7.750646
CDK5RAP3	CDK5 Regulatory Subunit Associated Protein 3	7.7484
REEP4	Receptor Accessory Protein 4	7.747243
MGST1	Microsomal Glutathione S-Transferase 1	7.740774
GORASP2	Golgi Reassembly Stacking Protein 2	7.734382
PDHA1	Pyruvate Dehydrogenase E1 Subunit Alpha 1	7.731648
PRL	Prolactin	7.727319
FKBP4	FKBP Prolyl Isomerase 4	7.708244
GRAMD1B	GRAM Domain Containing 1B	7.705703
AGER	Advanced Glycosylation End-Product Specific Receptor	7.67233
IL1A	Interleukin 1 Alpha	7.670585
ANXA5	Annexin A5	7.668034
TECR	Trans-2,3-Enoyl-CoA Reductase	7.666734
PROC	Protein C, Inactivator Of Coagulation Factors Va And VIIIa	7.665237
POR	Cytochrome P450 Oxidoreductase	7.656263
CHRNE	Cholinergic Receptor Nicotinic Epsilon Subunit	7.654804
NBAS	NBAS Subunit Of NRZ Tethering Complex	7.644092
SORT1	Sortilin 1	7.643346
REEP1	Receptor Accessory Protein 1	7.641009
CTSD	Cathepsin D	7.638577
SUMF2	Sulfatase Modifying Factor 2	7.63309
HTR2A	5-Hydroxytryptamine Receptor 2A	7.614561
SRP54	Signal Recognition Particle 54	7.600001
FAS	Fas Cell Surface Death Receptor	7.593858
PRDX3	Peroxiredoxin 3	7.588882
STARD3NL	STARD3 N-Terminal Like	7.582708
PTGIS	Prostaglandin I2 Synthase	7.579391
TGM2	Transglutaminase 2	7.578751
DGAT1	Diacylglycerol O-Acyltransferase 1	7.575037
STAT3	Signal Transducer And Activator Of Transcription 3	7.569951
SET	SET Nuclear Proto-Oncogene	7.566591
KCNJ2	Potassium Inwardly Rectifying Channel Subfamily J Member 2	7.563492
CCND1	Cyclin D1	7.550317
PKD1	Polycystin 1, Transient Receptor Potential Channel Interacting	7.545959
HSD17B12	Hydroxysteroid 17-Beta Dehydrogenase 12	7.52845
ANK1	Ankyrin 1	7.525622
TIA1	TIA1 Cytotoxic Granule Associated RNA Binding Protein	7.517891
NOTCH2	Notch Receptor 2	7.50958
TMEM259	Transmembrane Protein 259	7.503042
UBAC2	UBA Domain Containing 2	7.49613

ZFYVE27	Zinc Finger FYVE-Type Containing 27	7.493594
CDK5	Cyclin Dependent Kinase 5	7.4925
HAX1	HCLS1 Associated Protein X-1	7.477241
NOL3	Nucleolar Protein 3	7.47268
MICA	MHC Class I Polypeptide-Related Sequence A	7.470239
MR1	Major Histocompatibility Complex, Class I-Related	7.463374
CP	Ceruloplasmin	7.451293
HLA-C	Major Histocompatibility Complex, Class I, C	7.445433
PKM	Pyruvate Kinase M1/2	7.433251
STT3B	STT3 Oligosaccharyltransferase Complex Catalytic Subunit B	7.41882
RETREG1	Reticulophagy Regulator 1	7.414987
DPM1	Dolichyl-Phosphate Mannosyltransferase Subunit 1, Catalytic	7.407494
RNF183	Ring Finger Protein 183	7.402103
TRPA1	Transient Receptor Potential Cation Channel Subfamily A Member 1	7.40083
SRPRB	SRP Receptor Subunit Beta	7.398957
DNAJB14	DnaJ Heat Shock Protein Family (Hsp40) Member B14	7.397759
PPM1L	Protein Phosphatase, Mg2+/Mn2+ Dependent 1L	7.394979
IKBKG	Inhibitor Of Nuclear Factor Kappa B Kinase Regulatory Subunit Gamma	7.392099
DES	Desmin	7.392045
CTSB	Cathepsin B	7.389991
SSR2	Signal Sequence Receptor Subunit 2	7.355722
NGLY1	N-Glycanase 1	7.351737
CLCC1	Chloride Channel CLIC Like 1	7.349089
COPE	COPI Coat Complex Subunit Epsilon	7.337709
CREBBP	CREB Binding Protein	7.337461
PPP3CA	Protein Phosphatase 3 Catalytic Subunit Alpha	7.331773
NOS1	Nitric Oxide Synthase 1	7.327808
PML	PML Nuclear Body Scaffold	7.325724
MTHFR	Methylenetetrahydrofolate Reductase	7.323518
CRHR2	Corticotropin Releasing Hormone Receptor 2	7.322817
ICAM1	Intercellular Adhesion Molecule 1	7.321169
MAN1A1	Mannosidase Alpha Class 1A Member 1	7.317418
MAP2K3	Mitogen-Activated Protein Kinase Kinase 3	7.309654
ZFYVE1	Zinc Finger FYVE-Type Containing 1	7.309652
RNF5	Ring Finger Protein 5	7.304906
CYP1B1	Cytochrome P450 Family 1 Subfamily B Member 1	7.303506
PACS2	Phosphofurin Acidic Cluster Sorting Protein 2	7.303095
PIK3CG	Phosphatidylinositol-4,5-Bisphosphate 3-Kinase Catalytic Subunit Gamma	7.295561
FURIN	Furin, Paired Basic Amino Acid Cleaving Enzyme	7.291787
ECPAS	Ecm29 Proteasome Adaptor And Scaffold	7.28675
ELAVL1	ELAV Like RNA Binding Protein 1	7.284131
SYT2	Synaptotagmin 2	7.27916
ABCD1	ATP Binding Cassette Subfamily D Member 1	7.264944

EIF4E	Eukaryotic Translation Initiation Factor 4E	7.260617
EMC3	ER Membrane Protein Complex Subunit 3	7.258145
TRPM2	Transient Receptor Potential Cation Channel Subfamily M Member 2	7.253201
PITPNM1	Phosphatidylinositol Transfer Protein Membrane Associated 1	7.250624
LEP	Leptin	7.249093
CLGN	Calmegin	7.233139
SLC35B1	Solute Carrier Family 35 Member B1	7.214128
F5	Coagulation Factor V	7.207996
KRAS	KRAS Proto-Oncogene, GTPase	7.205581
RHBDD1	Rhomboid Domain Containing 1	7.199158
C1R	Complement C1r	7.193063
TRAPPC11	Trafficking Protein Particle Complex Subunit 11	7.18701
POMT1	Protein O-Mannosyltransferase 1	7.169538
SSR1	Signal Sequence Receptor Subunit 1	7.168171
DUSP19	Dual Specificity Phosphatase 19	7.167844
GHRL	Ghrelin And Obestatin Prepropeptide	7.157094
NLRP1	NLR Family Pyrin Domain Containing 1	7.155708
TRAPPC2	Trafficking Protein Particle Complex Subunit 2	7.153577
CYP3A4	Cytochrome P450 Family 3 Subfamily A Member 4	7.149347
SRP72	Signal Recognition Particle 72	7.144242
ZMPSTE24	Zinc Metallopeptidase STE24	7.138841
MYC	MYC Proto-Oncogene, BHLH Transcription Factor	7.129591
PPARGC1A	PPARG Coactivator 1 Alpha	7.128012
NPY	Neuropeptide Y	7.127833
CYP2C19	Cytochrome P450 Family 2 Subfamily C Member 19	7.123262
EMC4	ER Membrane Protein Complex Subunit 4	7.115891
GATA1	GATA Binding Protein 1	7.114007
MGAT2	Alpha-1,6-Mannosyl-Glycoprotein 2-Beta-N-Acetylglucosaminyltransferase	7.109999
KPNB1	Karyopherin Subunit Beta 1	7.095943
SEC31B	SEC31 Homolog B, COPII Coat Complex Component	7.087983
DNAJB2	DnaJ Heat Shock Protein Family (Hsp40) Member B2	7.087176
BTRC	Beta-Transducin Repeat Containing E3 Ubiquitin Protein Ligase	7.084516
CD74	CD74 Molecule	7.079303
BRCA2	BRCA2 DNA Repair Associated	7.078496
CLCN1	Chloride Voltage-Gated Channel 1	7.077934
PRDX5	Peroxiredoxin 5	7.075869
ADCYAP1	Adenylate Cyclase Activating Polypeptide 1	7.07373
EIF2B5	Eukaryotic Translation Initiation Factor 2B Subunit Epsilon	7.070683
FN1	Fibronectin 1	7.067998
CYB5A	Cytochrome B5 Type A	7.065848
DSPP	Dentin Sialophosphoprotein	7.062399
CD36	CD36 Molecule	7.054611
TANGO2	Transport And Golgi Organization 2 Homolog	7.052702

DST	Dystonin	7.049953
ATXN2	Ataxin 2	7.042354
STK25	Serine/Threonine Kinase 25	7.042146
MYDGF	Myeloid Derived Growth Factor	7.04205
PLOD3	Procollagen-Lysine,2-Oxoglutarate 5-Dioxygenase 3	7.038813
TNFRSF1A	TNF Receptor Superfamily Member 1A	7.036083
KNG1	Kininogen 1	7.031174
SCYL1	SCY1 Like Pseudokinase 1	7.027773
OXTR	Oxytocin Receptor	7.026897
ABCD4	ATP Binding Cassette Subfamily D Member 4	7.02663
TMEM117	Transmembrane Protein 117	7.020854
RTN2	Reticulon 2	7.017741
CDC42	Cell Division Cycle 42	7.012127
DNAJB1	DnaJ Heat Shock Protein Family (Hsp40) Member B1	7.008397
BCAP29	B Cell Receptor Associated Protein 29	7.004516
EMC2	ER Membrane Protein Complex Subunit 2	7.000575
PLA2G6	Phospholipase A2 Group VI	6.997512
S100A9	S100 Calcium Binding Protein A9	6.992437
GBA2	Glucosylceramidase Beta 2	6.982885
CALU	Calumenin	6.974298
ACTC1	Actin Alpha Cardiac Muscle 1	6.968117
ACTA1	Actin Alpha 1, Skeletal Muscle	6.965638
EMC6	ER Membrane Protein Complex Subunit 6	6.954175
GADD45A	Growth Arrest And DNA Damage Inducible Alpha	6.953204
JAGN1	Jagunal Homolog 1	6.947412
ATF2	Activating Transcription Factor 2	6.92938
MMGT1	Membrane Magnesium Transporter 1	6.925893
FANCD2	FA Complementation Group D2	6.922828
TMEM43	Transmembrane Protein 43	6.91492
COL4A1	Collagen Type IV Alpha 1 Chain	6.912389
CYB5R4	Cytochrome B5 Reductase 4	6.911352
PLOD2	Procollagen-Lysine,2-Oxoglutarate 5-Dioxygenase 2	6.908662
TERT	Telomerase Reverse Transcriptase	6.905632
SSR4	Signal Sequence Receptor Subunit 4	6.901724
VCPIP1	Valosin Containing Protein Interacting Protein 1	6.894663
SLC25A1	Solute Carrier Family 25 Member 1	6.886446
LMBRD1	LMBR1 Domain Containing 1	6.885598
USP19	Ubiquitin Specific Peptidase 19	6.881284
TRAPPC3	Trafficking Protein Particle Complex Subunit 3	6.881111
C1S	Complement C1s	6.875015
NOS1AP	Nitric Oxide Synthase 1 Adaptor Protein	6.870567
GOSR1	Golgi SNAP Receptor Complex Member 1	6.868047
HLA-DRA	Major Histocompatibility Complex, Class II, DR Alpha	6.863194

ELOVL4	ELOVL Fatty Acid Elongase 4	6.860619
SRP14	Signal Recognition Particle 14	6.850735
VAMP7	Vesicle Associated Membrane Protein 7	6.844652
NPLOC4	NPL4 Homolog, Ubiquitin Recognition Factor	6.836792
SAMD8	Sterile Alpha Motif Domain Containing 8	6.83396
RNF13	Ring Finger Protein 13	6.833418
SVIP	Small VCP Interacting Protein	6.829999
REEP5	Receptor Accessory Protein 5	6.820264
YWHAE	Tyrosine 3-Monooxygenase/Tryptophan 5-Monooxygenase Activation Protein Epsilon	6.817846
MARCHF6	Membrane Associated Ring-CH-Type Finger 6	6.816909
POP1	POP1 Homolog, Ribonuclease P/MRP Subunit	6.816401
TTN	Titin	6.814759
TTR	Transthyretin	6.812246
TYR	Tyrosinase	6.80928
SGPP2	Sphingosine-1-Phosphate Phosphatase 2	6.804559
TAPBPL	TAP Binding Protein Like	6.801363
KCNA2	Potassium Voltage-Gated Channel Subfamily A Member 2	6.801252
CIRBP	Cold Inducible RNA Binding Protein	6.796681
SLC39A7	Solute Carrier Family 39 Member 7	6.790615
FKBP10	FKBP Prolyl Isomerase 10	6.776898
EMC8	ER Membrane Protein Complex Subunit 8	6.774941
GRIN2A	Glutamate Ionotropic Receptor NMDA Type Subunit 2A	6.757884
ACSL4	Acyl-CoA Synthetase Long Chain Family Member 4	6.749694
HNRNPK	Heterogeneous Nuclear Ribonucleoprotein K	6.745708
CPQ	Carboxypeptidase Q	6.745594
COL2A1	Collagen Type II Alpha 1 Chain	6.731063
ADCYAP1R1	ADCYAP Receptor Type I	6.730136
RAB18	RAB18, Member RAS Oncogene Family	6.725442
SFTPC	Surfactant Protein C	6.724782
PIGT	Phosphatidylinositol Glycan Anchor Biosynthesis Class T	6.72053
SRI	Sorcin	6.714766
CES1	Carboxylesterase 1	6.700781
FKBP1A	FKBP Prolyl Isomerase 1A	6.694924
SGSH	N-Sulfoglucosamine Sulfohydrolase	6.686569
IL1RN	Interleukin 1 Receptor Antagonist	6.67561
DAPK1	Death Associated Protein Kinase 1	6.669888
LAMA2	Laminin Subunit Alpha 2	6.658468
MCL1	MCL1 Apoptosis Regulator, BCL2 Family Member	6.657077
SDHB	Succinate Dehydrogenase Complex Iron Sulfur Subunit B	6.65679
UGT1A9	UDP Glucuronosyltransferase Family 1 Member A9	6.647356
HMOX2	Heme Oxygenase 2	6.62949
PLA2G4C	Phospholipase A2 Group IVC	6.629183
RET	Ret Proto-Oncogene	6.620744

FUS	FUS RNA Binding Protein	6.617366
MAPK8IP1	Mitogen-Activated Protein Kinase 8 Interacting Protein 1	6.615273
NGF	Nerve Growth Factor	6.609324
RPS6KA3	Ribosomal Protein S6 Kinase A3	6.600644
ACE	Angiotensin I Converting Enzyme	6.591197
AKR1B1	Aldo-Keto Reductase Family 1 Member B	6.590053
HDAC6	Histone Deacetylase 6	6.589203
APOA1	Apolipoprotein A1	6.588792
SOD3	Superoxide Dismutase 3	6.588614
ATP7A	ATPase Copper Transporting Alpha	6.587573
VRK2	VRK Serine/Threonine Kinase 2	6.58393
RORA	RAR Related Orphan Receptor A	6.581261
DAXX	Death Domain Associated Protein	6.575673
F10	Coagulation Factor X	6.566853
MLEC	Malectin	6.560445
COL1A1	Collagen Type I Alpha 1 Chain	6.555603
GOSR2	Golgi SNAP Receptor Complex Member 2	6.554127
MMP9	Matrix Metallopeptidase 9	6.550004
NCK2	NCK Adaptor Protein 2	6.54836
CGRRF1	Cell Growth Regulator With Ring Finger Domain 1	6.547925
PLOD1	Procollagen-Lysine,2-Oxoglutarate 5-Dioxygenase 1	6.544441
BCHE	Butyrylcholinesterase	6.541703
CYP2B6	Cytochrome P450 Family 2 Subfamily B Member 6	6.541309
TXNRD1	Thioredoxin Reductase 1	6.540626
MAOB	Monoamine Oxidase B	6.538612
BGLAP	Bone Gamma-Carboxyglutamate Protein	6.535728
HRAS	HRas Proto-Oncogene, GTPase	6.533372
OPA1	OPA1 Mitochondrial Dynamin Like GTPase	6.530684
IGF2BP1	Insulin Like Growth Factor 2 MRNA Binding Protein 1	6.529144
AGTR1	Angiotensin II Receptor Type 1	6.528603
FGFR3	Fibroblast Growth Factor Receptor 3	6.525634
ALPP	Alkaline Phosphatase, Placental	6.525512
ROCK1	Rho Associated Coiled-Coil Containing Protein Kinase 1	6.517164
UCP2	Uncoupling Protein 2	6.51412
HACD3	3-Hydroxyacyl-CoA Dehydratase 3	6.511195
IL15RA	Interleukin 15 Receptor Subunit Alpha	6.507493
CDH1	Cadherin 1	6.499015
OMA1	OMA1 Zinc Metallopeptidase	6.498113
GRIN2B	Glutamate Ionotropic Receptor NMDA Type Subunit 2B	6.493698
FBN1	Fibrillin 1	6.492941
PIK3CA	Phosphatidylinositol-4,5-Bisphosphate 3-Kinase Catalytic Subunit Alpha	6.489425
USE1	Unconventional SNARE In The ER 1	6.487606
TRPM4	Transient Receptor Potential Cation Channel Subfamily M Member 4	6.486235

CAMLG	Calcium Modulating Ligand	6.474312
EMC9	ER Membrane Protein Complex Subunit 9	6.473402
EMD	Emerin	6.471211
ILVBL	IlvB Acetolactate Synthase Like	6.467979
ACER1	Alkaline Ceramidase 1	6.465769
EXT1	Exostosin Glycosyltransferase 1	6.463466
PTPN11	Protein Tyrosine Phosphatase Non-Receptor Type 11	6.453848
GDF15	Growth Differentiation Factor 15	6.450101
MTDH	Metadherin	6.446583
VDAC1	Voltage Dependent Anion Channel 1	6.446262
C2CD2L	C2CD2 Like	6.44539
MMP2	Matrix Metallopeptidase 2	6.445251
TBXAS1	Thromboxane A Synthase 1	6.442664
THBS4	Thrombospondin 4	6.431962
UBQLN2	Ubiquilin 2	6.430781
SCN1A	Sodium Voltage-Gated Channel Alpha Subunit 1	6.415355
RMRP	RNA Component Of Mitochondrial RNA Processing Endoribonuclease	6.403102
NOD2	Nucleotide Binding Oligomerization Domain Containing 2	6.395155
LRPAP1	LDL Receptor Related Protein Associated Protein 1	6.389328
RNF19B	Ring Finger Protein 19B	6.382182
UGT1A10	UDP Glucuronosyltransferase Family 1 Member A10	6.379853
GLUD1	Glutamate Dehydrogenase 1	6.372826
YWHAZ	Tyrosine 3-Monooxygenase/Tryptophan 5-Monooxygenase Activation Protein Zeta	6.370739
AGPAT1	1-Acylglycerol-3-Phosphate O-Acyltransferase 1	6.369559
RELA	RELA Proto-Oncogene, NF-KB Subunit	6.366933
ALG11	ALG11 Alpha-1,2-Mannosyltransferase	6.36377
LMAN1L	Lectin, Mannose Binding 1 Like	6.356157
FAAH	Fatty Acid Amide Hydrolase	6.349225
PRKCSH	Protein Kinase C Substrate 80K-H	6.344691
UFC1	Ubiquitin-Fold Modifier Conjugating Enzyme 1	6.340569
HUWE1	HECT, UBA And WWE Domain Containing E3 Ubiquitin Protein Ligase 1	6.340405
CBY1	Chibby Family Member 1, Beta Catenin Antagonist	6.339283
ENTPD5	Ectonucleoside Triphosphate Diphosphohydrolase 5 (Inactive)	6.335278
SORL1	Sortilin Related Receptor 1	6.334636
TRAPPC5	Trafficking Protein Particle Complex Subunit 5	6.333853
CREB3L4	CAMP Responsive Element Binding Protein 3 Like 4	6.317355
ATXN2L	Ataxin 2 Like	6.315902
EEF1B2	Eukaryotic Translation Elongation Factor 1 Beta 2	6.313234
DICER1	Dicer 1, Ribonuclease III	6.312777
DAB2IP	DAB2 Interacting Protein	6.31272
UFD1	Ubiquitin Recognition Factor In ER Associated Degradation 1	6.307011
GOLPH3	Golgi Phosphoprotein 3	6.30382
DYSF	Dysferlin	6.299904

PPP1CA	Protein Phosphatase 1 Catalytic Subunit Alpha	6.2995
SPTLC1	Serine Palmitoyltransferase Long Chain Base Subunit 1	6.295683
DCSTAMP	Dendrocyte Expressed Seven Transmembrane Protein	6.292465
TLR3	Toll Like Receptor 3	6.290827
TNFSF10	TNF Superfamily Member 10	6.289204
NR1H3	Nuclear Receptor Subfamily 1 Group H Member 3	6.288299
ADAMTSL1	ADAMTS Like 1	6.284753
ARFGAP2	ADP Ribosylation Factor GTPase Activating Protein 2	6.28356
SERPINE1	Serpin Family E Member 1	6.27388
AGRN	Agrin	6.273223
MAP3K7	Mitogen-Activated Protein Kinase Kinase Kinase 7	6.272805
LRP6	LDL Receptor Related Protein 6	6.270723
SERPINC1	Serpin Family C Member 1	6.26637
KPNA2	Karyopherin Subunit Alpha 2	6.26183
UBE2D3	Ubiquitin Conjugating Enzyme E2 D3	6.254593
ACP1	Acid Phosphatase 1	6.25178
B2M	Beta-2-Microglobulin	6.242842
ELOVL5	ELOVL Fatty Acid Elongase 5	6.241076
G6PC2	Glucose-6-Phosphatase Catalytic Subunit 2	6.240473
SNAP25	Synaptosome Associated Protein 25	6.233238
TEX2	Testis Expressed 2	6.227961
ARSA	Arylsulfatase A	6.22534
DHDDS	Dehydrodolichyl Diphosphate Synthase Subunit	6.223619
CHEK1	Checkpoint Kinase 1	6.220944
ATP13A2	ATPase Cation Transporting 13A2	6.220743
PXN	Paxillin	6.206064
LGALS1	Galectin 1	6.204163
BAG3	BAG Cochaperone 3	6.196301
CAPN2	Calpain 2	6.192875
NAPA	NSF Attachment Protein Alpha	6.191706
SSR3	Signal Sequence Receptor Subunit 3	6.190781
PDIA5	Protein Disulfide Isomerase Family A Member 5	6.18777
UGT1A8	UDP Glucuronosyltransferase Family 1 Member A8	6.179358
ATP5MK	ATP Synthase Membrane Subunit K	6.170422
CAMK2A	Calcium/Calmodulin Dependent Protein Kinase II Alpha	6.169222
GCH1	GTP Cyclohydrolase 1	6.164262
VMA21	Vacuolar ATPase Assembly Factor VMA21	6.154005
NPM1	Nucleophosmin 1	6.148796
GPAT3	Glycerol-3-Phosphate Acyltransferase 3	6.14737
MYH6	Myosin Heavy Chain 6	6.147057
PLD3	Phospholipase D Family Member 3	6.146048
BMP2	Bone Morphogenetic Protein 2	6.144058
BSG	Basigin (Ok Blood Group)	6.141879

ABCC8	ATP Binding Cassette Subfamily C Member 8	6.139851
PSMD2	Proteasome 26S Subunit Ubiquitin Receptor, Non-ATPase 2	6.133228
ATP2C1	ATPase Secretory Pathway Ca2+ Transporting 1	6.132174
F7	Coagulation Factor VII	6.127978
CRAT	Carnitine O-Acetyltransferase	6.12703
RPA1	Replication Protein A1	6.126224
PSENEN	Presenilin Enhancer, Gamma-Secretase Subunit	6.126016
RARA	Retinoic Acid Receptor Alpha	6.116398
NEPRO	Nucleolus And Neural Progenitor Protein	6.113357
KCNQ2	Potassium Voltage-Gated Channel Subfamily Q Member 2	6.11304
SIRT2	Sirtuin 2	6.105289
DPM3	Dolichyl-Phosphate Mannosyltransferase Subunit 3, Regulatory	6.093274
HLA-G	Major Histocompatibility Complex, Class I, G	6.08999
IL2	Interleukin 2	6.089747
PCNA	Proliferating Cell Nuclear Antigen	6.08961
EGR1	Early Growth Response 1	6.086165
GPAA1	Glycosylphosphatidylinositol Anchor Attachment 1	6.084425
ABCG1	ATP Binding Cassette Subfamily G Member 1	6.082954
CDIPT	CDP-Diacylglycerol--Inositol 3-Phosphatidyltransferase	6.082542
TRAPPC9	Trafficking Protein Particle Complex Subunit 9	6.069424
NPPB	Natriuretic Peptide B	6.064466
PLPP3	Phospholipid Phosphatase 3	6.055777
AFG3L2	AFG3 Like Matrix AAA Peptidase Subunit 2	6.055329
PDLIM1	PDZ And LIM Domain 1	6.049617
PRKD1	Protein Kinase D1	6.041307
HCRT	Hypocretin Neuropeptide Precursor	6.038782
PIGA	Phosphatidylinositol Glycan Anchor Biosynthesis Class A	6.032589
RPTOR	Regulatory Associated Protein Of MTOR Complex 1	6.03122
SIRT3	Sirtuin 3	6.030943
HSPG2	Heparan Sulfate Proteoglycan 2	6.030023
PIK3R2	Phosphoinositide-3-Kinase Regulatory Subunit 2	6.029199
HSPA6	Heat Shock Protein Family A (Hsp70) Member 6	6.027986
GRP	Gastrin Releasing Peptide	6.024777
VCL	Vinculin	6.018586
YIF1B	Yip1 Interacting Factor Homolog B, Membrane Trafficking Protein	6.017898
CAMK2D	Calcium/Calmodulin Dependent Protein Kinase II Delta	6.012105
PCK1	Phosphoenolpyruvate Carboxykinase 1	6.010427
DAD1	Defender Against Cell Death 1	6.001939
SLC6A1	Solute Carrier Family 6 Member 1	6.000302
DNAJA1	DnaJ Heat Shock Protein Family (Hsp40) Member A1	5.99088
STAT1	Signal Transducer And Activator Of Transcription 1	5.981032
COPG1	COPI Coat Complex Subunit Gamma 1	5.967433
ANKLE2	Ankyrin Repeat And LEM Domain Containing 2	5.958817

CYP17A1	Cytochrome P450 Family 17 Subfamily A Member 1	5.955261
SLC1A1	Solute Carrier Family 1 Member 1	5.954959
ITGB1	Integrin Subunit Beta 1	5.953186
TREM2	Triggering Receptor Expressed On Myeloid Cells 2	5.951803
ACTG1	Actin Gamma 1	5.949855
VPS33A	VPS33A Core Subunit Of CORVET And HOPS Complexes	5.945069
SLC37A1	Solute Carrier Family 37 Member 1	5.939048
KCNMA1	Potassium Calcium-Activated Channel Subfamily M Alpha 1	5.937607
FBXO6	F-Box Protein 6	5.932965
ABCA1	ATP Binding Cassette Subfamily A Member 1	5.92979
SELENOT	Selenoprotein T	5.92809
NOX5	NADPH Oxidase 5	5.926461
TLR2	Toll Like Receptor 2	5.926191
PARP16	Poly(ADP-Ribose) Polymerase Family Member 16	5.915741
COL13A1	Collagen Type XIII Alpha 1 Chain	5.913128
BID	BH3 Interacting Domain Death Agonist	5.90557
LAMP2	Lysosomal Associated Membrane Protein 2	5.901524
GPX8	Glutathione Peroxidase 8 (Putative)	5.89755
PLA2G4A	Phospholipase A2 Group IVA	5.890527
PPARA	Peroxisome Proliferator Activated Receptor Alpha	5.885859
LGI4	Leucine Rich Repeat LGI Family Member 4	5.881665
YBX1	Y-Box Binding Protein 1	5.880422
MSRA	Methionine Sulfoxide Reductase A	5.88028
UGT1A7	UDP Glucuronosyltransferase Family 1 Member A7	5.875714
CACNA1A	Calcium Voltage-Gated Channel Subunit Alpha1 A	5.87571
UQCRFS1	Ubiquinol-Cytochrome C Reductase, Rieske Iron-Sulfur Polypeptide 1	5.875269
FLT3	Fms Related Receptor Tyrosine Kinase 3	5.875015
RAP1GDS1	Rap1 GTPase-GDP Dissociation Stimulator 1	5.870473
WWOX	WW Domain Containing Oxidoreductase	5.86779
ARSH	Arylsulfatase Family Member H	5.865429
PIGC	Phosphatidylinositol Glycan Anchor Biosynthesis Class C	5.861403
ALDH3A2	Aldehyde Dehydrogenase 3 Family Member A2	5.859984
UGT1A4	UDP Glucuronosyltransferase Family 1 Member A4	5.857067
NACA	Nascent Polypeptide Associated Complex Subunit Alpha	5.855126
ALPL	Alkaline Phosphatase, Biomineralization Associated	5.854789
MSRB1	Methionine Sulfoxide Reductase B1	5.854691
COLGALT1	Collagen Beta(1-O)Galactosyltransferase 1	5.854337
SLC27A2	Solute Carrier Family 27 Member 2	5.843897
PIGB	Phosphatidylinositol Glycan Anchor Biosynthesis Class B	5.838052
CUL3	Cullin 3	5.824803
UGT1A3	UDP Glucuronosyltransferase Family 1 Member A3	5.821043
PMEL	Premelanosome Protein	5.818973
YTHDF2	YTH N6-Methyladenosine RNA Binding Protein 2	5.813521

TRAPPC4	Trafficking Protein Particle Complex Subunit 4	5.807836
TRA	T Cell Receptor Alpha Locus	5.805613
LPL	Lipoprotein Lipase	5.804234
KMT2B	Lysine Methyltransferase 2B	5.800021
SLC4A1	Solute Carrier Family 4 Member 1 (Diego Blood Group)	5.798791
UBQLN4	Ubiquilin 4	5.794048
ATP1A1	ATPase Na+/K+ Transporting Subunit Alpha 1	5.793483
TRPV1	Transient Receptor Potential Cation Channel Subfamily V Member 1	5.79318
SETD2	SET Domain Containing 2, Histone Lysine Methyltransferase	5.792985
PRKRA	Protein Activator Of Interferon Induced Protein Kinase EIF2AK2	5.788273
CYP19A1	Cytochrome P450 Family 19 Subfamily A Member 1	5.787447
LGALS3	Galectin 3	5.785028
POFUT1	Protein O-Fucosyltransferase 1	5.779183
YOD1	YOD1 Deubiquitinase	5.775467
TAOK3	TAO Kinase 3	5.774519
PTPRC	Protein Tyrosine Phosphatase Receptor Type C	5.768814
MIR34A	MicroRNA 34a	5.76838
DLAT	Dihydrolipoamide S-Acetyltransferase	5.758927
RPE65	Retinoid Isomerohydrolase RPE65	5.755983
GFAP	Glial Fibrillary Acidic Protein	5.755078
LRIT3	Leucine Rich Repeat, Ig-Like And Transmembrane Domains 3	5.751523
FAM120A	Family With Sequence Similarity 120A	5.74563
TRAF6	TNF Receptor Associated Factor 6	5.736246
EPHX1	Epoxide Hydrolase 1	5.728276
UCHL1	Ubiquitin C-Terminal Hydrolase L1	5.711159
UVRAG	UV Radiation Resistance Associated	5.708395
PLP1	Proteolipid Protein 1	5.704718
CDK2	Cyclin Dependent Kinase 2	5.703929
SI	Sucrase-Isomaltase	5.701946
PARG	Poly(ADP-Ribose) Glycohydrolase	5.698216
UBE2D2	Ubiquitin Conjugating Enzyme E2 D2	5.693721
CCDC88A	Coiled-Coil Domain Containing 88A	5.690186
SLC8A3	Solute Carrier Family 8 Member A3	5.688931
SCN10A	Sodium Voltage-Gated Channel Alpha Subunit 10	5.685342
EOGT	EGF Domain Specific O-Linked N-Acetylglucosamine Transferase	5.684913
EIF5A	Eukaryotic Translation Initiation Factor 5A	5.683418
NPC2	NPC Intracellular Cholesterol Transporter 2	5.683301
KCNN4	Potassium Calcium-Activated Channel Subfamily N Member 4	5.682719
AGT	Angiotensinogen	5.682107
ELOVL2	ELOVL Fatty Acid Elongase 2	5.679664
ANXA2	Annexin A2	5.677977
PIGH	Phosphatidylinositol Glycan Anchor Biosynthesis Class H	5.675057
NSFL1C	NSFL1 Cofactor	5.6745

TM7SF2	Transmembrane 7 Superfamily Member 2	5.671021
DPM2	Dolichyl-Phosphate Mannosyltransferase Subunit 2, Regulatory	5.670761
XIAP	X-Linked Inhibitor Of Apoptosis	5.665239
TH	Tyrosine Hydroxylase	5.6622
PIGS	Phosphatidylinositol Glycan Anchor Biosynthesis Class S	5.657073
BAD	BCL2 Associated Agonist Of Cell Death	5.644003
PSMA7	Proteasome 20S Subunit Alpha 7	5.641157
RACK1	Receptor For Activated C Kinase 1	5.639627
HTR1A	5-Hydroxytryptamine Receptor 1A	5.638844
USP13	Ubiquitin Specific Peptidase 13	5.638432
VAMP1	Vesicle Associated Membrane Protein 1	5.638091
PNKD	PNKD Metallo-Beta-Lactamase Domain Containing	5.636946
WDR83OS	WD Repeat Domain 83 Opposite Strand	5.634799
USP9X	Ubiquitin Specific Peptidase 9 X-Linked	5.634021
H2AX	H2A.X Variant Histone	5.6283
ADAMTS13	ADAM Metallopeptidase With Thrombospondin Type 1 Motif 13	5.62463
UGT1A	UDP Glucuronosyltransferase Family 1 Member A Complex Locus	5.623094
CASP1	Caspase 1	5.622622
FITM2	Fat Storage Inducing Transmembrane Protein 2	5.619159
MET	MET Proto-Oncogene, Receptor Tyrosine Kinase	5.617685
USP14	Ubiquitin Specific Peptidase 14	5.608085
ARF1	ADP Ribosylation Factor 1	5.600962
PPIA	Peptidylprolyl Isomerase A	5.598885
ASL	Argininosuccinate Lyase	5.598268
SEC22A	SEC22 Homolog A, Vesicle Trafficking Protein	5.597146
IL18	Interleukin 18	5.589247
PTPA	Protein Phosphatase 2 Phosphatase Activator	5.578484
RASGRF2	Ras Protein Specific Guanine Nucleotide Releasing Factor 2	5.570591
TGFA	Transforming Growth Factor Alpha	5.570003
PPIF	Peptidylprolyl Isomerase F	5.567487
TRAP1	TNF Receptor Associated Protein 1	5.564984
LRRC59	Leucine Rich Repeat Containing 59	5.561094
HSPA1L	Heat Shock Protein Family A (Hsp70) Member 1 Like	5.558681
KL	Klotho	5.54951
RAB3GAP1	RAB3 GTPase Activating Protein Catalytic Subunit 1	5.54733
NRG1	Neuregulin 1	5.546505
FASLG	Fas Ligand	5.546445
CYP2A6	Cytochrome P450 Family 2 Subfamily A Member 6	5.545689
TBL2	Transducin Beta Like 2	5.537586
PMAIP1	Phorbol-12-Myristate-13-Acetate-Induced Protein 1	5.535959
CYP2C9	Cytochrome P450 Family 2 Subfamily C Member 9	5.535381
CDKAL1	CDK5 Regulatory Subunit Associated Protein 1 Like 1	5.534067
HP	Haptoglobin	5.533607

PEMT	Phosphatidylethanolamine N-Methyltransferase	5.526129
VKORC1L1	Vitamin K Epoxide Reductase Complex Subunit 1 Like 1	5.522895
HTR3A	5-Hydroxytryptamine Receptor 3A	5.52095
MEF2C	Myocyte Enhancer Factor 2C	5.520911
PDHB	Pyruvate Dehydrogenase E1 Subunit Beta	5.520752
ELOVL7	ELOVL Fatty Acid Elongase 7	5.519201
NDUFS4	NADH:Ubiquinone Oxidoreductase Subunit S4	5.514392
HSPA2	Heat Shock Protein Family A (Hsp70) Member 2	5.514015
COPB2	COPI Coat Complex Subunit Beta 2	5.508985
CDKN2A	Cyclin Dependent Kinase Inhibitor 2A	5.5031
ATG9A	Autophagy Related 9A	5.501772
TFEB	Transcription Factor EB	5.501767
TRAPPC12	Trafficking Protein Particle Complex Subunit 12	5.495687
ACSL3	Acyl-CoA Synthetase Long Chain Family Member 3	5.49466
CSNK2A1	Casein Kinase 2 Alpha 1	5.493505
CHP1	Calcineurin Like EF-Hand Protein 1	5.492865
TRPM8	Transient Receptor Potential Cation Channel Subfamily M Member 8	5.483677
UGT1A5	UDP Glucuronosyltransferase Family 1 Member A5	5.477101
TTF2	Transcription Termination Factor 2	5.472316
IAPP	Islet Amyloid Polypeptide	5.471467
PNPLA6	Patatin Like Phospholipase Domain Containing 6	5.467666
PURA	Purine Rich Element Binding Protein A	5.466282
FLNB	Filamin B	5.463548
CERS6	Ceramide Synthase 6	5.463155
ARRB1	Arrestin Beta 1	5.459452
NF1	Neurofibromin 1	5.458903
TESPA1	Thymocyte Expressed, Positive Selection Associated 1	5.44838
HNRNPU	Heterogeneous Nuclear Ribonucleoprotein U	5.44686
HLA-DRB1	Major Histocompatibility Complex, Class II, DR Beta 1	5.445292
MAN2B1	Mannosidase Alpha Class 2B Member 1	5.444276
KCNB1	Potassium Voltage-Gated Channel Subfamily B Member 1	5.442124
LIPC	Lipase C, Hepatic Type	5.439047
RUVBL2	RuvB Like AAA ATPase 2	5.433352
CFLAR	CASP8 And FADD Like Apoptosis Regulator	5.431244
PALS1	Protein Associated With LIN7 1, MAGUK P55 Family Member	5.429113
SMPD2	Sphingomyelin Phosphodiesterase 2	5.427896
RPL10	Ribosomal Protein L10	5.426992
SPCS2	Signal Peptidase Complex Subunit 2	5.42576
SMN1	Survival Of Motor Neuron 1, Telomeric	5.421015
NCLN	Nicalin	5.415156
SERPINI1	Serpin Family I Member 1	5.413888
HADHB	Hydroxyacyl-CoA Dehydrogenase Trifunctional Multienzyme Complex Subunit Beta	5.410623
CDH2	Cadherin 2	5.408671

NIBAN1	Niban Apoptosis Regulator 1	5.406737
UBE2N	Ubiquitin Conjugating Enzyme E2 N	5.398006
AGPAT2	1-Acylglycerol-3-Phosphate O-Acyltransferase 2	5.397935
GFPT1	Glutamine--Fructose-6-Phosphate Transaminase 1	5.39397
MYRF	Myelin Regulatory Factor	5.393103
BRAF	B-Raf Proto-Oncogene, Serine/Threonine Kinase	5.392915
PRKDC	Protein Kinase, DNA-Activated, Catalytic Subunit	5.390024
ULBP1	UL16 Binding Protein 1	5.388706
PRKAB1	Protein Kinase AMP-Activated Non-Catalytic Subunit Beta 1	5.386183
LIN28A	Lin-28 Homolog A	5.383773
RIC3	RIC3 Acetylcholine Receptor Chaperone	5.382417
DRD1	Dopamine Receptor D1	5.381422
DUOXA1	Dual Oxidase Maturation Factor 1	5.381156
ANKS4B	Ankyrin Repeat And Sterile Alpha Motif Domain Containing 4B	5.380867
HSD11B2	Hydroxysteroid 11-Beta Dehydrogenase 2	5.379252
OLR1	Oxidized Low Density Lipoprotein Receptor 1	5.378887
KCNIP4	Potassium Voltage-Gated Channel Interacting Protein 4	5.375228
MEF2A	Myocyte Enhancer Factor 2A	5.370764
LMNB1	Lamin B1	5.370135
CASR	Calcium Sensing Receptor	5.359273
SEC22C	SEC22 Homolog C, Vesicle Trafficking Protein	5.356649
SERPINA2	Serpin Family A Member 2 (Gene/Pseudogene)	5.352215
SELP	Selectin P	5.343926
EIF2B1	Eukaryotic Translation Initiation Factor 2B Subunit Alpha	5.341355
PEX11B	Peroxisomal Biogenesis Factor 11 Beta	5.340438
AVP	Arginine Vasopressin	5.338967
PLCG1	Phospholipase C Gamma 1	5.338659
PDE5A	Phosphodiesterase 5A	5.338113
ACBD3	Acyl-CoA Binding Domain Containing 3	5.336448
QDPR	Quinoid Dihydropteridine Reductase	5.334661
CR1	Complement C3b/C4b Receptor 1 (Knops Blood Group)	5.328671
TIAL1	TIA1 Cytotoxic Granule Associated RNA Binding Protein Like 1	5.327433
ZDHHC4	Zinc Finger DHHC-Type Palmitoyltransferase 4	5.325793
EEF1D	Eukaryotic Translation Elongation Factor 1 Delta	5.316687
TPM1	Tropomyosin 1	5.310005
TMEM67	Transmembrane Protein 67	5.307762
CHRM3	Cholinergic Receptor Muscarinic 3	5.306836
LPCAT1	Lysophosphatidylcholine Acyltransferase 1	5.305692
ATF1	Activating Transcription Factor 1	5.304689
PTK2	Protein Tyrosine Kinase 2	5.301264
GOLGB1	Golgin B1	5.297478
CSTB	Cystatin B	5.295172
LDHA	Lactate Dehydrogenase A	5.280085

CASC3	CASC3 Exon Junction Complex Subunit	5.276394
ERBB2	Erb-B2 Receptor Tyrosine Kinase 2	5.261974
ATG7	Autophagy Related 7	5.261068
CLN5	CLN5 Intracellular Trafficking Protein	5.257618
NCSTN	Nicastrin	5.253432
SYNCRIP	Synaptotagmin Binding Cytoplasmic RNA Interacting Protein	5.252891
PLG	Plasminogen	5.252563
NUP210	Nucleoporin 210	5.251059
SGF29	SAGA Complex Associated Factor 29	5.250876
YY1	YY1 Transcription Factor	5.250799
ELOVL1	ELOVL Fatty Acid Elongase 1	5.246082
MUC5AC	Mucin 5AC, Oligomeric Mucus/Gel-Forming	5.240655
NRAS	NRAS Proto-Oncogene, GTPase	5.233254
SLC9A1	Solute Carrier Family 9 Member A1	5.22897
OPRM1	Opioid Receptor Mu 1	5.226197
HNF4A	Hepatocyte Nuclear Factor 4 Alpha	5.214128
NSDHL	NAD(P) Dependent Steroid Dehydrogenase-Like	5.209011
STEEP1	STING1 ER Exit Protein 1	5.205729
FGF2	Fibroblast Growth Factor 2	5.204324
TOR1AIP1	Torsin 1A Interacting Protein 1	5.204095
MIR199A1	MicroRNA 199a-1	5.202199
AIMP1	Aminoacyl TRNA Synthetase Complex Interacting Multifunctional Protein 1	5.200974
PEF1	Penta-EF-Hand Domain Containing 1	5.197813
STARD5	StAR Related Lipid Transfer Domain Containing 5	5.190176
TRPC1	Transient Receptor Potential Cation Channel Subfamily C Member 1	5.189981
DLG1	Discs Large MAGUK Scaffold Protein 1	5.18822
EZH2	Enhancer Of Zeste 2 Polycomb Repressive Complex 2 Subunit	5.186925
CDKN1B	Cyclin Dependent Kinase Inhibitor 1B	5.175326
CYP21A2	Cytochrome P450 Family 21 Subfamily A Member 2	5.172648
MYLK	Myosin Light Chain Kinase	5.167694
E2F1	E2F Transcription Factor 1	5.166516
SRL	Sarcalumenin	5.164198
KRT8	Keratin 8	5.16344
TLR7	Toll Like Receptor 7	5.162696
CTH	Cystathionine Gamma-Lyase	5.158203
BLZF1	Basic Leucine Zipper Nuclear Factor 1	5.155042
GH1	Growth Hormone 1	5.15438
POMP	Proteasome Maturation Protein	5.150156
RPLP0	Ribosomal Protein Lateral Stalk Subunit P0	5.145832
PTP4A1	Protein Tyrosine Phosphatase 4A1	5.145557
IGF1R	Insulin Like Growth Factor 1 Receptor	5.144852
STXBP1	Syntaxin Binding Protein 1	5.143266
OPRD1	Opioid Receptor Delta 1	5.137342

UBE2D1	Ubiquitin Conjugating Enzyme E2 D1	5.13348
TMEM199	Transmembrane Protein 199	5.13261
NR4A1	Nuclear Receptor Subfamily 4 Group A Member 1	5.13198
TJP1	Tight Junction Protein 1	5.131021
RPS6	Ribosomal Protein S6	5.128533
GABRA1	Gamma-Aminobutyric Acid Type A Receptor Subunit Alpha1	5.120553
EPAS1	Endothelial PAS Domain Protein 1	5.120019
TRAM2	Translocation Associated Membrane Protein 2	5.119758
NDUFS8	NADH:Ubiquinone Oxidoreductase Core Subunit S8	5.113416
DGAT2	Diacylglycerol O-Acyltransferase 2	5.112139
SPAG5	Sperm Associated Antigen 5	5.109059
TSC1	TSC Complex Subunit 1	5.107747
CCK	Cholecystokinin	5.105546
UBXN2B	UBX Domain Protein 2B	5.10526
NPPA	Natriuretic Peptide A	5.104582
SLC2A4	Solute Carrier Family 2 Member 4	5.099747
DEGS1	Delta 4-Desaturase, Sphingolipid 1	5.092605
DYNC1H1	Dynein Cytoplasmic 1 Heavy Chain 1	5.090978
GTF2I	General Transcription Factor IIi	5.088943
ELOVL3	ELOVL Fatty Acid Elongase 3	5.087617
PROS1	Protein S	5.084815
MATN3	Matrilin 3	5.07702
FGFR4	Fibroblast Growth Factor Receptor 4	5.074791
HLA-DPB1	Major Histocompatibility Complex, Class II, DP Beta 1	5.073803
ENPP1	Ectonucleotide Pyrophosphatase/Phosphodiesterase 1	5.069609
DYRK1A	Dual Specificity Tyrosine Phosphorylation Regulated Kinase 1A	5.069344
CCL4	C-C Motif Chemokine Ligand 4	5.068934
BIRC2	Baculoviral IAP Repeat Containing 2	5.066642
DHX36	DEAH-Box Helicase 36	5.066614
F3	Coagulation Factor III, Tissue Factor	5.058293
CD59	CD59 Molecule (CD59 Blood Group)	5.053978
DELE1	DAP3 Binding Cell Death Enhancer 1	5.051995
MECP2	Methyl-CpG Binding Protein 2	5.051294
STK39	Serine/Threonine Kinase 39	5.049914
LRP2	LDL Receptor Related Protein 2	5.048332
KRTCAP2	Keratinocyte Associated Protein 2	5.047668
ABCC6	ATP Binding Cassette Subfamily C Member 6	5.046158
RAF1	Raf-1 Proto-Oncogene, Serine/Threonine Kinase	5.044864
NAT8	N-Acetyltransferase 8 (Putative)	5.042976
PREP	Prolyl Endopeptidase	5.038263
C6orf120	Chromosome 6 Open Reading Frame 120	5.036865
DDHD1	DDHD Domain Containing 1	5.035842
GCLC	Glutamate-Cysteine Ligase Catalytic Subunit	5.031579

C3orf52	Chromosome 3 Open Reading Frame 52	5.031522
CYP51A1	Cytochrome P450 Family 51 Subfamily A Member 1	5.028956
HACE1	HECT Domain And Ankyrin Repeat Containing E3 Ubiquitin Protein Ligase 1	5.027467
MIR21	MicroRNA 21	5.025929
VDR	Vitamin D Receptor	5.025618
PRKCQ	Protein Kinase C Theta	5.020879
LSG1	Large 60S Subunit Nuclear Export GTPase 1	5.020027
CCN2	Cellular Communication Network Factor 2	5.018164
GPI	Glucose-6-Phosphate Isomerase	5.017212
PGRMC1	Progesterone Receptor Membrane Component 1	5.016309
LAMP1	Lysosomal Associated Membrane Protein 1	5.012759
SPP1	Secreted Phosphoprotein 1	5.010533
FGF21	Fibroblast Growth Factor 21	5.010385
ATXN3	Ataxin 3	5.009679
RPS3	Ribosomal Protein S3	5.007141
MYO9A	Myosin IXA	5.005795
TGFBR1	Transforming Growth Factor Beta Receptor 1	5.003658
CAST	Calpastatin	5.002892
ACSF3	Acyl-CoA Synthetase Family Member 3	5.002577
ACER3	Alkaline Ceramidase 3	5.000251
